# Phenotypic Test of Benzo[4,5]imidazo[1,2-c]pyrimidinone-Based Nucleoside and Non-Nucleoside Derivatives against DNA and RNA Viruses, Including Coronaviruses

**DOI:** 10.3390/ijms241914540

**Published:** 2023-09-26

**Authors:** Polina Kamzeeva, Ivan Petushkov, Ekaterina Knizhnik, Robert Snoeck, Yuri Khodarovich, Ekaterina Ryabukhina, Vera Alferova, Artur Eshtukov-Shcheglov, Evgeny Belyaev, Julia Svetlova, Tatiana Vedekhina, Andrey Kulbachinskiy, Anna Varizhuk, Graciela Andrei, Andrey Aralov

**Affiliations:** 1 Shemyakin-Ovchinnikov Institute of Bioorganic Chemistry, Russian Academy of Sciences, 117997 Moscow, Russia; polinabast@yandex.ru (P.K.); miss.katringet@yandex.ru (E.R.); alferovava@gmail.com (V.A.); nemtcev.94.nmr@gmail.com (A.E.-S.); 2 Institute of Molecular Genetics, National Research Centre ‘Kurchatov Institute’, 123182 Moscow, Russia; telomer1@rambler.ru (I.P.); avkulb@yandex.ru (A.K.); 3 Institute of Gene Biology, Russian Academy of Sciences, 119334 Moscow, Russia; 4 Lopukhin Federal Research and Clinical Center of Physical-Chemical Medicine of Federal Medical Biological Agency, 119435 Moscow, Russia; knizhnik.ek@gmail.com (E.K.); j.i.svetlova@gmail.com (J.S.); neglect1@yandex.ru (T.V.); annavarizhuk@niifhm.ru (A.V.); 5 Moscow Institute of Physics and Technology, 141701 Dolgoprudny, Russia; 6 Rega Institute for Medical Research, KU Leuven, 3000 Leuven, Belgium; robert.snoeck@kuleuven.be (R.S.); graciela.andrei@kuleuven.be (G.A.); 7 Frumkin Institute of Physical Chemistry and Electrochemistry, RAS, 119071 Moscow, Russia; cronse@yandex.ru

**Keywords:** antiviral activity, tricyclic nucleoside analogs, benzo[4,5]imidazo[1,2-c]pyrimidinone, SARS-CoV-2, human coronavirus, respiratory syncytial virus, varicella zoster virus, liquid–liquid phase separation, RdRp

## Abstract

Emerging and re-emerging viruses periodically cause outbreaks and epidemics around the world, which ultimately lead to global events such as the COVID-19 pandemic. Thus, the urgent need for new antiviral drugs is obvious. Over more than a century of antiviral development, nucleoside analogs have proven to be promising agents against diversified DNA and RNA viruses. Here, we present the synthesis and evaluation of the antiviral activity of nucleoside analogs and their deglycosylated derivatives based on a hydroxybenzo[4,5]imidazo[1,2-c]pyrimidin-1(2H)-one scaffold. The antiviral activity was evaluated against a panel of structurally and phylogenetically diverse RNA and DNA viruses. The leader compound showed micromolar activity against representatives of the family *Coronaviridae*, including SARS-CoV-2, as well as against respiratory syncytial virus in a submicromolar range without noticeable toxicity for the host cells. Surprisingly, methylation of the aromatic hydroxyl group of the leader compound resulted in micromolar activity against the varicella-zoster virus without any significant impact on cell viability. The leader compound was shown to be a weak inhibitor of the SARS-CoV-2 RNA-dependent RNA polymerase. It also inhibited biocondensate formation important for SARS-CoV-2 replication. The active compounds may be considered as a good starting point for further structure optimization and mechanistic and preclinical studies.

## 1. Introduction

Over the past two decades, humanity has been afflicted by an increasing number of viral epidemics, including H1N1 influenza, Ebola, and the COVID-19 outbreaks/pandemic [1,2,3,4]. Unfortunately, there is often a lack of effective treatment options for emerging or re-emerging viruses. Developing new antiviral drugs is a time-consuming task, so, when urgent action is required, modifying existing drug scaffolds and repurposing approved drugs is considered the best approach [5].

Nucleoside analogs (NAs) are considered a good choice for quick and efficient development of antiviral therapeutics. Most NAs inhibit viral replication through chain termination or lethal mutagenesis [6,7,8], but they can also affect the efficiency of the binding of a viral nucleic acid to polymerases and inhibit viral methyltransferases and a number of host cell enzymes that are essential for virus replication, such as those involved in nucleotide synthesis and RNA capping [9,10,11]. The efficacy and safety profiles of NAs have been extensively studied, and over 30 NAs have already been approved by the FDA [12,13]. On the other hand, recently discovered features on the mechanism of action and the proposed complex modifications of NAs reveal that, despite their long history, NAs are still prospective for investigation of novel antiviral drugs [14,15].

**Figure 1:**
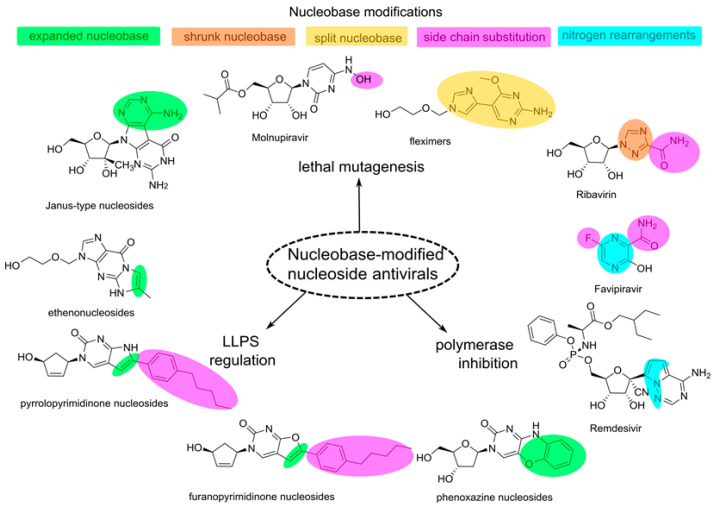
Examples of nucleobase modifications in various antivirals.

Nucleoside analogs are derivatives of natural nucleosides with a modified sugar residue and/or nucleobase. Sugar modification has been extensively used to obtain viral polymerase inhibitors [7], but the potential of nucleobase modifications has been less explored. Modifications of nucleobases can be classified as follows: (1) nitrogen substitution and rearrangements in the heterocycle (aza and deaza derivatives), (2) side-chain substitution, (3) heterocycle splitting, and (4) nucleobases expansion (Figure 1). Modifications of the first type can be used to increase the stability of the drug, as demonstrated in the case of remdesivir [16]. Many NAs with a modified nucleobase act through lethal mutagenesis, including ribavirin, favipiravir, molnupiravir, and Janus-type nucleosides, however, the latter are less promising due to their high cytotoxicity [6,17]. Fleximers carrying split nucleobases demonstrate mutagenic activity, as well as the ability to inhibit viral polymerases and methyltransferases [18,19]. Etheno derivatives with acyclic sugar residues have demonstrated antiviral activity against viruses from the family *Herpesviridae*, but they have been shown to be low-effective prodrugs of antivirals with natural nucleobases [20,21]. Phenoxazine derivatives attract attention due to a wide spectrum of activity against RNA and DNA viruses [22]. Their mechanism of action has not been studied, but the incorporation of phenoxazine nucleotides into the growing chain of the viral nucleic acid might lead to a suboptimal secondary structure [22]. Bicyclic NAs, such as furanopyrimidinone and pyrrolopyrimidinone-based derivatives, are a promising class of antivirals [23], including the most effective inhibitor of VZV known to date [24]. A series of furanopyrimidinone derivatives with exclusive activity against VZV can only be phosphorylated by VZV kinases, but their target and further metabolic fate remain unknown [25]. Pyrrolopyrimidinones are capable of inhibiting the activity of coronavirus polymerase without incorporation into the growing chain [26]. At the same time, both pyrrolo- and furanopyrimidinones can influence liquid–liquid phase separation (LLPS) of the viral nucleocapsid protein (N) and viral genomic RNA (vgRNA) [27]. LLPS affects viral packaging and viral genome replication through the recruitment of viral polymerases, thus representing a prospective process to be targeted by antivirals [28].

**Figure 2:**
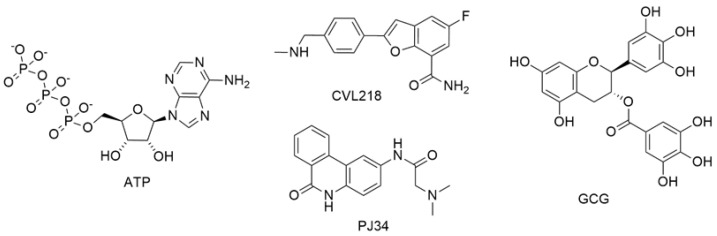
Examples of LLPS modulators.

LLPS has been recognized as a new target for antiviral development due to its important role in the viral life cycle [29,30]. LLPS provides the formation of non-membrane organelles, where enrichment in specific biomolecules is achieved to increase the efficiency of biological processes [29,31]. DNA damage foci, promyelocytic leukemia nuclear bodies (PML nuclear bodies), Cajal bodies, processing bodies (P-bodies), and stress granules are prominent examples of host cell biocondensates. Viruses also utilize LLPS to ensure viral genome replication and assembly, as was demonstrated for a number of viruses, including SARS-CoV-2 and HIV-1 [29,30,32,33]. Furthermore, viruses can evade antiviral immune response through LLPS regulation and biocondensate formation [29]. As LLPS is significant for viral replication, small-molecule LLPS modulators are being developed and assessed as antivirals (Figure 2) [33]. Among endogenous molecules, ATP was shown to modulate SARS-CoV-2 N protein condensation with vgRNA [34]. Gallocatechin-3-gallate (GCG) effectively binds to the N protein, disrupts biocondensate formation, and demonstrates moderate antiviral activity [35]. CVL218 and PJ34 decrease the density of SARS-CoV-2 N-protein:vgRNA biocondensates and facilitate access to viral replication machinery for remdesivir [36]. Nucleoside skeletons with modified nucleobases are also promising scaffolds for obtaining LLPS-modulating antivirals, as was demonstrated by pyrrolo- and furanopyrimidinones nucleoside derivatives [27].

Here, we have synthesized a set of nucleoside and non-nucleoside derivatives based on an aromatic system extended in comparison with the parent cytosine nucleobase and tested them for their ability to suppress viral replication in vitro of a large panel of DNA and RNA viruses. In addition, the ability of the leader compound to inhibit the activity of SARS-CoV-2 RNA-dependent RNA polymerase (RdRp) and modulate the formation of biomolecular condensates important for SARS-CoV-2 transcription and replication has been evaluated.

## 2. Results and Discussion

### 2.1. Chemistry

**Scheme 1:**
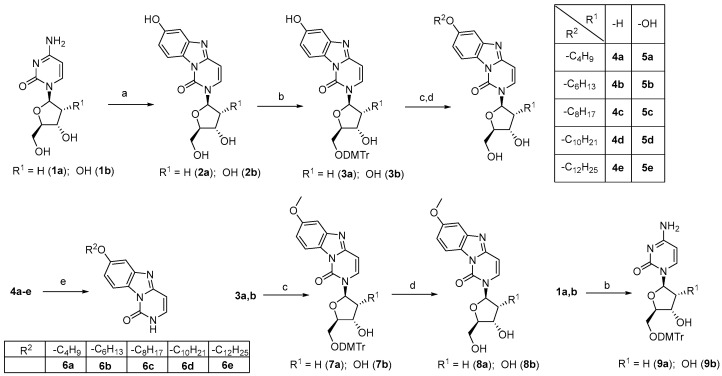
The preparation of the benzo[4,5]imidazo[1,2-c]pyrimidin-1(2H)-one derivatives. Reagents and conditions: (**a**) 1,4-benzoquinone, sodium acetate buffer (pH 4.5), 37 °C; (**b**) DMTr-Cl, Py, rt; (**c**) C_n_H_2n+1_OH (n = 1 (**7a**,**b**), 4 (**4a** and **5a**), 6 (**4b** and **5b**), 8 (**4c** and **5c**), 10 (**4d** and **5d**), 12 (**4e** and **5e**)), PPh_3_, DIAD, CH_2_Cl_2_, 0 °C→rt; (**d**) CH_3_COOH, H_2_O, 50 °C; and (**e**) 12 N HCl, CH_3_OH, 50 °C.

The synthesis began with the extension of the cytosine heteroaromatic system. To create the benzo[4,5]imidazo[1,2-c]pyrimidinonyl system, a reported method for 2′-deoxycytidine as a starting material was used [37]. 2′-Deoxycytidine **1a** or cytidine **1b** was treated with 1,4-benzoquinone in a sodium acetate buffer (pH 4.5), affording the corresponding tricyclic derivatives **2a** and **2b**, respectively (Scheme 1). After 5’-*O*-dimethoxytritylation (DMTr) of **2a**,**b**, the resulting compounds (**3a**,**b**) were subjected to Mitsunobu coupling with C_n_H_2n+1_OH (n = 4, 6, 8, 10 or 12), followed by DMTr removal in mild acidic conditions (aqueous CH_3_COOH, 50 °C), yielding the target candidate antivirals **4a**–**e** and **5a**–**e** of the 2′-deoxyribo- and ribonucleoside series, respectively. Finally, to synthesize the tricyclic non-nucleoside derivatives **6a**–**e**, we exploited the instability of the glycosidic bond of the 2′-deoxyribo series and treated **4a**–**e** with hydrochloric acid in CH_3_OH. Based on activity studies for the above derivatives, an additional set of compounds was prepared. Mitsunobu reaction was applied to condensate **3a**,**b** with CH_3_OH to prepare **7a**,**b**, and their 5′-O-deblocking provided **8a**,**b**. In addition, **1a**,**b** was also 5′-O-dimethoxytritylated for the preparation of **9a**,**b**, two control compounds for evaluating the role of the tricyclic moiety in the manifestation of antiviral activity.

### 2.2. Biological Evaluation

Compounds from the first synthetic round, namely **2a**,**b**, **3a**,**b**, **4a**–**e**, **5a**–**e**, and **6a**–**e**, were evaluated for activity against DNA viruses, including varicella zoster virus (VZV), cytomegalovirus (HCMV), and herpes simplex virus-1 (HSV-1), as well as against RNA viruses, including human coronavirus (HCoV), respiratory syncytial virus (RSV), yellow fever virus (YFV), influenza A virus (IAV), and influenza B virus (IBV) (see Table S1). Ganciclovir, cidofovir, acyclovir, brivudine, remdesivir, ribavirin, zanamivir, and dextran sulfate (molecular weight 10,000, DS.−10,000) were used as reference compounds. Antiviral activity was expressed as EC_50_, the effective concentration required to reduce virus plaque formation (VZV) by 50% or to reduce the virus-induced cytopathic effect by 50% (other viruses).

All compounds tested can be divided into three groups: (1) 2′-O-deoxyribonucleoside (**2a**, **3a**, **4a**–**e**), (2) ribonucleoside (**2b**, **3b**, **5a**–**e**), and (3) non-nucleoside (**6a**–**e**) series. Among them, only two 5′-O-DMTr-substituted compounds (**3a** and **3b**) exhibited activity against two HCoVs, namely 229E and OC43, with EC_50_ values in the micromolar and submicromolar range, respectively, without any signs of potency against another HCoV strain NL63 (Table 1). These compounds also showed an inhibitory effect on RSV, with **3a** having an order of magnitude lower EC_50_ than **3b**. However, the activity was accompanied by cytotoxicity for the Huh7 cell line. The in vitro efficiency of tritylated and 4,4′-dimethoxytritylated pyrimidine and purine nucleoside and non-nucleoside analogs against diverse viruses, including representatives of families *Flaviviridae* and *Herpesviridae*, was previously reported [22,38,39]. The results emphasize the importance of the DMTr group in both nucleoside series for their activity, regardless of the sugar moiety. Meanwhile, non-nucleoside derivatives from the third group did not show any noticeable potency. There was no significant activity against VZV, HCMV, HSV-1, YFV, IAV, and IBV.

Then, several analogs of the leader compounds **3a**,**b** were synthesized, namely derivatives **7a**,**b** bearing an OCH_3_ group at the seventh position and their 5′-O-deblocked congeners **8a**,**b**. To evaluate the contribution of the tricyclic system to antiviral activity, 5′-O-DMTr-2′-deoxycytidine **9a** and 5′-O-DMTr-cytidine **9b** were also synthesized. The compounds were tested against VZV (TK^+^ and TK^−^ strains) and HCMV (AD-169 and Davis strains) (Table 2). Surprisingly, the methylation of the 7-OH group, which was unsubstituted in the inactive compounds **3a**,**b**, led to anti-VZV activity, whereas there was no activity against HCMV. The inhibitory effect was noticeably greater against the thymidine kinase-deficient TK^−^ VZV strain, suggesting a kinase-independent mechanism of action. Importantly, the activity of **7a** was not accompanied by noticeable cytotoxicity, which provides a promising index of selectivity (SI = CC_50_/EC_50_) of more than 30 for the TK-deficient VZV strain. The lack of activity of **8a**,**b** and **9a**,**b** emphasizes the importance of the DMTr group and the substituted tricyclic heteroaromatic system.

Finally, the set of the second-round compounds was evaluated on a panel of various SARS-CoV-2 strains (Table 3). Only compound **3a** from the 2′-O-deoxyribose series had anti-SARS-CoV-2 activity, however, affecting cell morphology at ≥20 μM concentration.

### 2.3. Inhibition of RdRp Activity In Vitro

**Figure 3:**
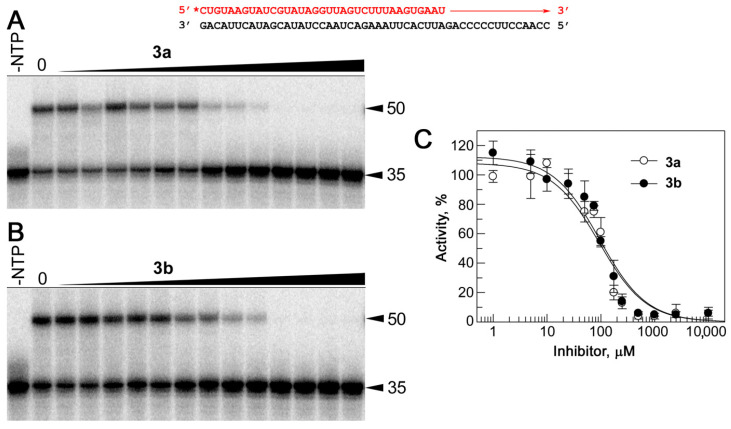
The effects of compounds **3a** and **3b** on the activity of RdRp in vitro. The RNA substrate, consisting of an RNA primer ^32^P-labeled at the 5′-end (red) and the template strand (black), is shown on the top. The direction of primer extension by RdRp is indicated by the red arrow. (**A**,**B**) Analysis of RNA products at various concentrations of **3a** (**A**) and **3b** (**B**). Positions of the RNA primer and the extended full-length RNA product are indicated by black arrows. The length of RNA is indicated. (**C**) Quantitative analysis of the inhibition of RdRp activity by **3a** and **3b**. Means and standard deviations from three independent measurements are shown. The data were fitted to a hyperbolic equation (see Section 3.3.2 for details).

Since the leader compound **3a** is a 5′-O-dimethoxytrityl nucleoside derivative with activity against various representatives of the family *Coronaviridae*, viral RNA-dependent RNA polymerase (RdRp) may be considered as its potential target. Indeed, RNA viruses, including coronaviruses, share conserved core sequences and structural features of RdRp [40]. 3′,5′-di-O-trityluridine was shown to inhibit DENV polymerase with IC_50_ values ranging from 3.1 to 6.9 μM by affecting RNA elongation rather than by the initiation of a DENV-specific “minigenomic” RNA template [38]. To verify this assumption, we analyzed the effects of the leader **3a** from the deoxyribose series and its close analog (**3b)**, which is inactive in the CPE reduction assay, on the activity of the SARS-CoV-2 transcription/replication complex in vitro. For this purpose, we purified the RdRp holoenzyme, consisting of the catalytic subunit nsp12 and two accessory subunits (nsp7 and nsp), and tested its activity with a double-stranded RNA substrate containing a radiolabeled primer and the template strand in the presence of various inhibitor concentrations. Previously, we showed that the system could be used as a good model for studying substrate specificity and testing RdRp inhibitors [26,41]. We examined the effect of the inhibitors on RdRp activity in concentrations ranging from 1 µM to 10 mM (Figure 3). To our surprise, the inhibition activity of compounds **3a** and **3b** was very similar. Both substances were transcriptional inhibitors with almost identical IC_50_ values, 94 ± 3 μM and 95 ± 4 μM for **3a** and **3b**, respectively.

These values significantly differ from the data on antiviral activity for compound **3a**, which has a much lower EC_50_ value than **3b** (by at least one order of magnitude) (Table 3). In contrast to our results, several previously tested nucleoside inhibitors had close values of EC_50_ and IC_50_, measured in almost the same in vitro system [26]. Thus, RdRp may not be the primary target for **3a**, suggesting the presence of other viral or cellular targets.

### 2.4. Inhibition of Biocondensate Formation In Vitro

In vivo transcription and replication of SARS-CoV-2 requires the assembly of biomacromolecular condensates on the vgRNA through LLPS [42]. This process is driven by transient interactions of the partly disordered N-protein, which binds stem-loop-rich vgRNA fragments and acts as a condensate “scaffold” [43]. RdRp enters the condensates as a “client” [42]. Its accumulation within the condensates ensures timely vgRNA replication. We assumed the observed anti-SARS-CoV-2 activity of **3a** may be associated with the disruption of these condensates.

**Figure 4:**
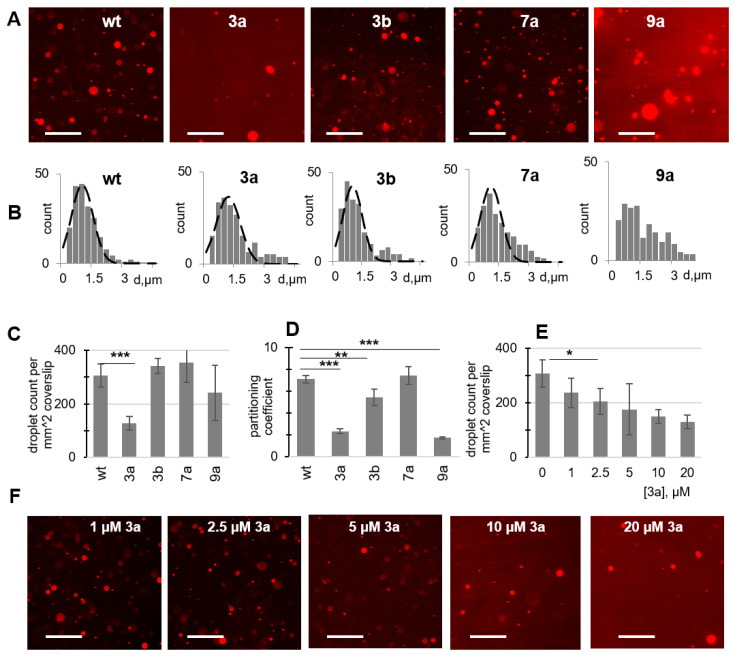
The effects of the antiviral compounds on biocondensate formation in vitro: cell-free LLPS assays. (**A**) Fluorescence microscopy images of the condensates assembled from the labeled SARS-CoV-2 N-protein (3 µM) and the gRNA fragment (6 µM) in the absence (wt) and in the presence of the compounds (20 µM). Scale bar: 10 µm. Additional representative large-field images are provided in Supplementary Information. (**B**) Size distribution of condensates obtained in the absence (wt) and in the presence of the compounds (20 µM). (**C**) Summary of the compounds’ effects on condensate formation estimated as the average number of droplets per slide (**D**) Antivirals’ effects on the partitioning coefficient of the N-protein. (**E**) Dose-dependence of the effects of compound **3a**. (**F**) Representative fluorescence microscopy images illustrating the dose-dependent effects of **3a**. Scale bar: 10 µm. Error bars in C-E represent the SD of six repeats. * *p* < 0.05; ** *p* < 0.001; *** *p* < 0.0001 (two-tailed Student’s *t*-test).

Compound **3a** is somewhat similar to previously reported condensate-modulating nucleoside analogs [27], so we verified its activity in LLPS assays. For that, we used model condensates assembled from fluorescently labeled SARS-CoV-2 N-protein and the stem-loop vgRNA fragment, as was described previously [27]. In parallel to the active compound **3a**, we tested three inactive derivatives as negative controls, namely **3b** with an additional 2′-OH, **7a** with an additional methylation of the 7-OH group, and 5′-O-DMTr 2′-deoxycytidine **9a**, a derivative without the tricyclic heteroaromatic system. In the absence of the compounds, the condensates were visualized by fluorescence microscopy as droplets of 0.9 ± 0.6 micrometer diameter (Figure 4A,B and Figure S1). They floated and coalesced, consistently with the liquid phase. They were morphologically analogous to previously observed condensates and distinct from reported gel-like structures or solid aggregates [27].

Unlike the negative controls **3b**, **7a,** and **9a**, compound **3a** showed significant (*p* < 0.0001) inhibition of condensate formation; the average number of droplets decreased by approximately 60% in the presence of 20 µM **3a** (Figure 4C). The partitioning coefficient (droplet/solution N-protein concentration ratio) also decreased by 60–70% in the presence of **3a** (*p* < 0.0001), while **3b** had a much smaller effect (Figure 4D). Surprisingly, **9a** caused a dramatic (approximately 4-fold) decrease in the partitioning coefficient; however, the droplets remained clearly visible, and many of them were enlarged, resulting in a broad and multimodal size distribution (Figure 4B). In the case of **3a** (median droplet diameter equal to 1.1 µm) and other compounds (median droplet diameter equal to 0.9 µm), size distribution changes were minor to moderate. To summarize, **9a** reduced droplet density but did not prevent LLPS, **7a** caused no changes, the effect of **3b** was minor, and **3a** was the only robust LLPS inhibitor. The effect of **3a** was dose-dependent (Figure 4E,F) with EC_50_ ≤ 10 µM. We conclude that the pronounced antiviral activity of **3a** in Vero cells may result, in part, from a combination of LLPS inhibition and direct RdRp inhibition. Importantly, proper orientation of the polar OH groups on the heteroaromatic backbone is essential for LLPS inhibition.

## 3. Materials and Methods

### 3.1. Chemistry

#### 3.1.1. General

All reagents were purchased from commercial sources and were used without further purification. Anhydrous CH_2_Cl_2_ and pyridine were obtained by distillation over calcium hydride. NMR spectra were recorded on a Bruker Avance III 600 spectrometer (Bruker, Rheinstetten, Germany) at 600 and 150 MHz for ^1^H and ^13^C spectra, respectively, or on a Bruker Fourier 300 spectrometer (Bruker, Rheinstetten, Germany) at 300 and 75 MHz for ^1^H and ^13^C spectra, respectively. Chemical shifts were reported relative to the residue peaks of DMSO-d_6_ (2.50 ppm for ^1^H and 39.5 ppm for ^13^C) and CDCl_3_ (7.26 ppm for ^1^H and 77.0 ppm for ^13^C). Thin layer chromatography (TLC) was performed on Merck TLC Silica gel 60F_254_ plates with spot visualization with a UV lamp at 254 nm. Silica gel (silica gel 60, 0.040–0.063 mm) from Merck (Darmstadt, Germany) was used for column chromatography. ESI HR mass spectra were acquired on an LTQ FT Ultra (Thermo Electron Corp., Bremen, Germany) mass spectrometer in negative ion mode.

#### 3.1.2. Synthesis and Characterization of Compounds

**General procedure for the preparation of nucleoside derivatives with a tricyclic aromatic system**

To a solution of nucleoside 1 (9.50 mmol) in 0.1 M sodium acetate buffer (250 mL, pH 4.5), 1,4-benzoquinone (4.31 g, 40.0 mmol, 4.2 equiv) was added and the reaction mixture was stirred at 37 °C for 24 h. The solvent was removed under reduced pressure and the residue was purified using column chromatography on silica gel (0–13% CH_3_OH in CH_2_Cl_2_), yielding the product as a beige amorphous solid.

**7-hydroxy-2-((2R,4S,5R)-4-hydroxy-5-(hydroxymethyl)tetrahydrofuran-2-yl)benzo[4,5]imidazo[1,2-c]pyrimidin-1(2H)-one 2a**

Yield 59%. ^1^H NMR (600 MHz, DMSO-d_6_): δ 9.59 (s, 1H), 7.81 (d, J = 7.9 Hz, 1H), 7.75 (s, 1H), 7.53 (d, J = 8.5 Hz, 1H), 6.93 (d, J = 8.3 Hz, 1H), 6.66 (d, J = 7.9 Hz, 1H), 6.46 (t, J = 6.5 Hz, 1H), 5.30 (d, J = 3.3 Hz, 1H), 5.06 (t, J = 4.7 Hz, 1H), 4.34–4.29 (m, 1H), 3.89–3.84 (m, 1H), 3.67–3.58 (m, 2H), 2.26–2.19 (m, 2H). ^13^C NMR (150 MHz, DMSO-d_6_): δ 153.97, 146.79, 146.74, 137.18, 130.90, 130.46, 119.03, 114.51, 100.47, 98.12, 87.56, 84.70, 70.35, 61.22, 39.96. HRMS (ESI) *m*/*z*: calcd for C_15_H_14_N_3_O_5_^−^ [M-H]^−^: 316.0939; found 316.0901.

**2-((2R,3R,4S,5R)-3,4-dihydroxy-5-(hydroxymethyl)tetrahydrofuran-2-yl)-7-hydroxybenzo[4,5]imidazo[1,2-c]pyrimidin-1(2H)-one 2b**

Yield 57%. ^1^H NMR (600 MHz, DMSO-d_6_): δ 9.61 (s, 1H), 7.84 (d, J = 8.1 Hz, 1H), 7.76 (d, J = 2.4 Hz, 1H), 7.54 (d, J = 8.7 Hz, 1H), 6.94 (dd, J = 8.7 Hz, J = 2.4 Hz, 1H), 6.68 (d, J = 8.1 Hz, 1H), 6.10 (d, J = 5.5 Hz, 1H), 5.43 (d, J = 5.6 Hz, 1H), 5.16 (t, J = 5.1 Hz, 1H), 5.13 (d, J = 5.0 Hz, 1H), 4.18–4.15 (m, 1H), 4.07–4.04 (m, 1H), 3.95–3.93 (m, 1H), 3.71–3.67 (m, 1H), 3.64–3.60 (m, 1H). ^13^C NMR (150 MHz, DMSO-d_6_): δ 154.03, 147.06, 146.72, 137.15, 131.07, 130.48, 119.08, 114.54, 100.46, 98.12, 88.19, 85.09, 73.95, 69.93, 60.83. HRMS (ESI) *m*/*z*: calcd for C_15_H_14_N_3_O_6_^−^ [M-H]^−^: 332.0888; found 332.0853.

**General procedure for introducing the 5′-O-DMTr group**

Compound **1** or **2** (3.00 mmol) was co-evaporated with anhydrous pyridine (20 mL), then dissolved in anhydrous pyridine (80 mL), and 4,4′-dimethoxytrityl chloride (1.22 g, 3.60 mmol, 1.2 equiv) was added. After 5 h at RT, the reaction was stopped by the addition of an aqueous saturated NaHCO_3_ solution (50 mL), and the organics were extracted with CH_2_Cl_2_ (2 × 50 mL). The combined organic layers were concentrated and co-evaporated with toluene (3 × 10 mL). Purification was performed using column chromatography on silica gel (0–4% CH_3_OH in CH_2_Cl_2_ with 0.1% TEA), yielding **3a**,**b** and **9a**,**b** as yellowish and off-white amorphous solids, respectively.

**2-((2R,4S,5R)-5-((bis(4-methoxyphenyl)(phenyl)methoxy)methyl)-4-hydroxytetrahydrofuran-2-yl)-7-hydroxybenzo[4,5]imidazo[1,2-c]pyrimidin-1(2H)-one 3a**

Yield 80%. ^1^H NMR (300 MHz, DMSO-d_6_): δ 9.54 (s, 1H), 7.76 (d, J = 2.4 Hz, 1H), 7.58 (d, J = 8.1 Hz, 1H), 7.53 (d, J = 8.7 Hz, 1H), 7.43–7.38 (m, 2H), 7.35–7.19 (m, 7H), 6.94 (dd, J = 8.7 Hz, J = 2.5 Hz, 1H), 6.92–6.85 (m, 4H), 6.45 (t, J = 6.4 Hz, 1H), 6.39 (d, J = 8.1 Hz, 1H), 5.34 (d, J = 4.7 Hz, 1H), 4.42–4.34 (m, 1H), 4.00–3.94 (m, 1H), 3.73 (s, 3H), 3.73 (s, 3H), 3.32–3.26 (m, 2H), 2.37–2.30 (m, 2H). ^13^C NMR (75 MHz, DMSO-d_6_): δ 157.99 (2C), 154.01, 146.61 (2C), 144.56, 137.15, 135.35, 135.16, 130.74, 130.45, 129.63 (4C), 127.73 (2C), 127.63 (2C), 126.61, 119.03, 114.53, 113.10 (4C), 100.45, 97.86, 85.72, 85.47, 84.73, 69.84, 63.27, 54.89 (2C), 39.43 (overlaps with DMSO). HRMS (ESI) *m*/*z*: calcd for C_36_H_32_N_3_O_7_^−^ [M-H]^−^: 618.2246; found 618.2161.

**2-((2R,3R,4S,5R)-5-((bis(4-methoxyphenyl)(phenyl)methoxy)methyl)-3,4-dihydroxytetrahydrofuran-2-yl)-7-hydroxybenzo[4,5]imidazo[1,2-c]pyrimidin-1(2H)-one 3b**

Yield 53%. ^1^H NMR (300 MHz, DMSO-d_6_): δ 9.58 (s, 1H), 7.77 (d, J = 2.2 Hz, 1H), 7.66 (d, J = 8.1 Hz, 1H), 7.54 (d, J = 8.7 Hz, 1H), 7.45–7.39 (m, 2H), 7.37–7.21 (m, 7H), 6.98–6.87 (m, 5H), 6.34 (d, J = 8.0 Hz, 1H), 6.07 (d, J = 3.5 Hz, 1H), 5.51 (d, J = 4.8 Hz, 1H), 5.15 (d, J = 5.6 Hz, 1H), 4.28–4.17 (m, 2H), 4.09–4.02 (m, 1H), 3.74 (s, 6H), 3.36–3.31 (m, 2H). ^13^C NMR (75 MHz, DMSO-d_6_): δ 158.08 (2C), 154.12, 146.83, 146.66, 144.63, 137.16, 135.35, 135.12, 131.00, 130.52, 129.75 (4C), 127.88 (2C), 127.69 (2C), 126.75, 119.14, 114.62, 113.21 (4C), 100.50, 97.81, 89.43, 85.86, 82.60, 73.84, 69.53, 62.84, 54.97, 54.85. HRMS (ESI) *m*/*z*: calcd for C_36_H_32_N_3_O_8_^−^ [M-H]^−^: 634.2195; found 634.2121.

**4-amino-1-((2R,4S,5R)-5-((bis(4-methoxyphenyl)(phenyl)methoxy)methyl)-4-hydroxytetrahydrofuran-2-yl)pyrimidin-2(1H)-one 9a**

Yield 89%. ^1^H NMR (600 MHz, DMSO-d_6_): δ 7.69 (d, J = 7.5 Hz, 1H), 7.53 (br s, 2H), 7.39–7.36 (m, 2H), 7.33–7.30 (m, 2H), 7.27–7.22 (m, 5H), 6.91–6.88 (m, 4H), 6.15 (t, J = 6.3 Hz, 1H), 5.62 (d, J = 7.5 Hz, 1H), 5.30 (d, J = 4.3 Hz, 1H), 4.27–4.23 (m, 1H), 3.90–3.86 (m, 1H), 3.74 (s, 6H), 3.22 (dd, J = 10.6 Hz, J = 5.0 Hz, 1H), 3.19 (dd, J = 10.6 Hz, J = 3.4 Hz, 1H). ^13^C NMR (150 MHz, DMSO-d_6_): δ 164.50, 158.00 (2C), 153.70, 144.57, 140.93, 135.32, 135.21, 129.60 (4C), 127.74 (2C), 127.59 (2C), 126.64, 113.11 (4C), 93.70, 85.64, 85.13, 84.67, 69.80, 63.23, 54.94 (2C), 40.27. HRMS (ESI) *m*/*z*: calcd for C_30_H_30_N_3_O_6_^−^ [M-H]^−^: 528.2140; found 528.2098.

**4-amino-1-((2R,3R,4S,5R)-5-((bis(4-methoxyphenyl)(phenyl)methoxy)methyl)-3,4-dihydroxytetrahydrofuran-2-yl)pyrimidin-2(1H)-one 9b**

Yield 79%. ^1^H NMR (600 MHz, DMSO-d_6_): δ 7.81 (d, J = 7.5 Hz, 1H), 7.64 (br s, 2H), 7.40–7.37 (m, 2H), 7.34–7.30 (m, 2H), 7.28–7.23 (m, 5H), 6.91–6.88 (m, 4H), 5.77 (d, J = 2.9 Hz, 1H), 5.58 (d, J = 7.5 Hz, 1H), 5.44 (d, J = 3.9 Hz, 1H), 5.06 (d, J = 6.0 Hz, 1H), 4.11–4.06 (m, 1H), 4.00–3.94 (m, 2H), 3.75 (s, 6H), 3.28–3.23 (m, 2H). ^13^C NMR (150 MHz, DMSO-d_6_): δ 164.31, 158.01 (2C), 153.56, 144.55, 141.31, 135.33, 135.16, 129.60 (4C), 127.76 (2C), 127.60 (2C), 126.66, 113.13 (4C), 93.59, 89.84, 85.68, 81.66, 73.92, 69.08, 62.54, 54.93 (2C). HRMS (ESI) *m*/*z*: calcd for C_30_H_30_N_3_O_7_^−^ [M-H]^−^: 544.2089; found 544.2063.

**General procedure for the preparation of 7-O-alkylated nucleoside derivatives**

To a stirred mixture of compound **3** (0.11 mmol), PPh_3_ (58 mg, 0.22 mmol, 2 equiv) and the corresponding C_n_H_2n+1_OH alcohol (n = 4, 6, 8, 10, 12) (0.15 mmol, 1.3 equiv) in dry CH_2_Cl_2_ (5 mL) DIAD (43 μL, 0.22 mmol, 2 equiv) was added at 0 °C. The reaction mixture was stirred at RT overnight and concentrated under reduced pressure. The residue was dissolved in a mixture of CH_3_COOH/H_2_O (2 mL, 1:9, *v*:*v*) and kept at 50 °C for 8 h. After concentration and co-evaporation with toluene (3 × 10 mL), the residue was purified using column chromatography on silica gel (0–6% CH_3_OH in CH_2_Cl_2_), yielding **4a**–**e** and **5a**–**e** as a yellowish amorphous solid.

**2-((2R,4S,5R)-5-((bis(4-methoxyphenyl)(phenyl)methoxy)methyl)-4-hydroxytetrahydrofuran-2-yl)-7-butoxybenzo[4,5]imidazo[1,2-c]pyrimidin-1(2H)-one 4a**

Yield 82%. ^1^H NMR (300 MHz, DMSO-d_6_): δ 7.87(d, J = 8.0 Hz, 1H), 7.85 (dd, J = 2.6 Hz, 1H), 7.62 (d, J = 8.8 Hz, 1H), 7.09 (dd, J = 8.8 Hz, J = 2.6 Hz, 1H), 6.68 (d, J = 8.0 Hz, 1H), 6.46 (t, J = 6.7 Hz, 1H), 4.35–4.30 (m, 1H), 4.05 (t, J = 6.5 Hz, 2H), 3.91–3.86 (m, 1H), 3.67 (dd, J = 11.9 Hz, J = 3.6 3.62 (dd, J = 11.9 Hz, J = 4.0 2.29–2.21 (m, 2H), 1.81–1.69 (m, 2H), 1.55–1.41 (m, 2H), 0.96 (t, J = 7.4 Hz, 3H). ^13^C NMR (75 MHz, DMSO-d_6_): δ 155.25, 147.36, 146.65, 137.96, 131.33, 130.31, 119.05, 114.59, 99.12, 97.92, 87.62, 84.87, 70.20, 67.77, 61.12, 40.10, 30.65, 18.61, 13.56. HRMS (ESI) *m*/*z*: calcd for C_40_H_40_N_3_O_7_^−^ [M-H]^−^: 674.2872; found 674.2856.

**2-((2R,4S,5R)-5-((bis(4-methoxyphenyl)(phenyl)methoxy)methyl)-4-hydroxytetrahydrofuran-2-yl)-7-(hexyloxy)benzo[4,5]imidazo[1,2-c]pyrimidin-1(2H)-one 4b**

Yield 82%. ^1^H NMR (600 MHz, DMSO-d_6_): δ 7.87(d, J = 8.1 Hz, 1H), 7.84 (d, J = 2.5 Hz, 1H), 7.62 (d, J = 8.8 Hz, 1H), 7.08 (dd, J = 8.8 Hz, J = 2.5 Hz, 1H), 6.69 (d, J = 8.1 Hz, 1H), 6.46 (t, J = 6.7 Hz, 1H), 5.30 (br s, 1H), 5.07 (br s, 1H), 4.34–4.30 (m, 1H), 4.03 (t, J = 6.4 Hz, 2H), 3.90–3.86 (m, 1H), 3.66 (dd, J = 11.8 Hz, J = 3.6 3.62 (dd, J = 11.8 Hz, J = 3.8 2.28–2.20 (m, 2H), 1.78–1.72 (m, 2H), 1.48–1.41 (m, 2H), 1.35–1.28 (m, 4H), 0.88 (t, J = 7.0 Hz, 3H). ^13^C NMR (150 MHz, DMSO-d_6_): δ 155.25, 147.37, 146.68, 138.05, 131.29, 130.33, 119.09, 114.58, 99.09, 97.98, 87.62, 84.86, 70.22, 68.07, 61.13, 40.11, 30.89, 28.56, 25.07, 21.94, 13.77. HRMS (ESI) *m*/*z*: calcd for C_42_H_44_N_3_O_7_^−^ [M-H]^−^: 702.3185; found 702.3150.

**2-((2R,4S,5R)-5-((bis(4-methoxyphenyl)(phenyl)methoxy)methyl)-4-hydroxytetrahydrofuran-2-yl)-7-(octyloxy)benzo[4,5]imidazo[1,2-c]pyrimidin-1(2H)-one 4c**

Yield 73%. ^1^H NMR (600 MHz, DMSO-d_6_): δ 7.87(d, J = 8.1 Hz, 1H), 7.84 (d, J = 2.4 Hz, 1H), 7.62 (d, J = 8.8 Hz, 1H), 7.07 (dd, J = 8.8 Hz, J = 2.4 Hz, 1H), 6.69 (d, J = 8.1 Hz, 1H), 6.46 (t, J = 6.7 Hz, 1H), 5.31 (br s, 1H), 5.08 (br s, 1H), 4.33–4.30 (m, 1H), 4.03 (t, J = 6.4 Hz, 2H), 3.89–3.87 (m, 1H), 3.66 (dd, J = 11.8 Hz, J = 3.6 3.62 (dd, J = 11.8 Hz, J = 3.7 2.28–2.19 (m, 2H), 1.78–1.71 (m, 2H), 1.47–1.40 (m, 2H), 1.36–1.21 (m, 8H), 0.86 (t, J = 6.9 Hz, 3H). ^13^C NMR (150 MHz, DMSO-d_6_): δ 155.24, 147.37, 146.69, 138.09, 131.26, 130.34, 119.11, 114.57, 99.10, 98.00, 87.62, 84.86, 70.23, 68.06, 61.14, 40.11, 31.10, 28.63, 28.59, 28.53, 25.40, 21.95, 13.80. HRMS (ESI) *m*/*z*: calcd for C_44_H_48_N_3_O_7_^−^ [M-H]^−^: 730.3498; found 730.3487.

**2-((2R,4S,5R)-5-((bis(4-methoxyphenyl)(phenyl)methoxy)methyl)-4-hydroxytetrahydrofuran-2-yl)-7-(decyloxy)benzo[4,5]imidazo[1,2-c]pyrimidin-1(2H)-one 4d**

Yield 79%. ^1^H NMR (300 MHz, DMSO-d_6_): δ 7.86 (d, J = 8.1 Hz, 1H), 7.84 (dd, J = 2.5 Hz, 1H), 7.61 (d, J = 8.8 Hz, 1H), 7.08 (dd, J = 8.8 Hz, J = 2.5 Hz, 1H), 6.68 (d, J = 8.1 Hz, 1H), 6.46 (t, J = 6.7 Hz, 1H), 4.36–4.29 (m, 1H), 4.04 (t, J = 6.5 Hz, 2H), 3.91–3.86 (m, 1H), 3.67 (dd, J = 11.9 Hz, J = 3.8 3.62 (dd, J = 11.9 Hz, J = 4.0 2.30–2.19 (m, 2H), 1.81–1.69 (m, 2H), 1.50–1.37 (m, 2H), 1.37–1.18 (m, 12H), 0.85 (t, J = 6.7 Hz, 3H). ^13^C NMR (75 MHz, DMSO-d_6_): δ 155.24, 147.36, 146.67, 138.06, 131.27, 130.33, 119.08, 114.56, 99.11, 97.98, 87.62, 84.86, 70.22, 68.05, 61.13, 40.11, 31.15, 28.86, 28.81, 28.64, 28.56 (2C), 25.37, 21.94, 13.79. HRMS (ESI) *m*/*z*: calcd for C_46_H_52_N_3_O_7_^−^ [M-H]^−^: 758.3811; found 758.3777.

**2-((2R,4S,5R)-5-((bis(4-methoxyphenyl)(phenyl)methoxy)methyl)-4-hydroxytetrahydrofuran-2-yl)-7-(dodecyloxy)benzo[4,5]imidazo[1,2-c]pyrimidin-1(2H)-one 4e**

Yield 88%. ^1^H NMR (600 MHz, DMSO-d_6_): δ 7.87 (d, J = 8.0 Hz, 1H), 7.84 (d, J = 2.0 Hz, 1H), 7.62 (d, J = 8.7 Hz, 1H), 7.07 (dd, J = 8.7 Hz, J = 2.0 Hz, 1H), 6.69 (d, J = 8.0 Hz, 1H), 6.46 (t, J = 6.5 Hz, 1H), 5.31 (br s, 1H), 5.08 (br s, 1H), 4.35–4.29 (m, 1H), 4.03 (t, J = 5.9 Hz, 2H), 3.91–3.86 (m, 1H), 3.65 (dd, J = 11.1 Hz, J = 2.9 3.61 (dd, J = 11.9 Hz, J = 3.1 2.29–2.19 (m, 2H), 1.80–1.70 (m, 2H), 1.48–1.39 (m, 2H), 1.37–1.17 (m, 16H), 0.84 (t, J = 6.6 Hz, 3H). ^13^C NMR (150 MHz, DMSO-d_6_): δ 155.24, 147.37, 146.69, 138.12, 131.25, 130.35, 119.12, 114.57, 99.11, 98.02, 87.62, 84.85, 70.23, 68.05, 61.14, 40.12, 31.16, 28.90, 28.87, 28.84 (2C), 28.62, 28.57 (2C), 25.37, 21.96, 13.80. HRMS (ESI) *m*/*z*: calcd for C_48_H_56_N_3_O_7_^−^ [M-H]^−^: 786.4124; found 786.4194.

**2-((2R,3R,4S,5R)-5-((bis(4-methoxyphenyl)(phenyl)methoxy)methyl)-3,4-dihydroxytetrahydrofuran-2-yl)-7-butoxybenzo[4,5]imidazo[1,2-c]pyrimidin-1(2H)-one 5a**

Yield 69%. ^1^H NMR (300 MHz, DMSO-d_6_): δ 7.92 (d, J = 8.1 Hz, 1H), 7.87 (d, J = 2.5 Hz, 1H), 7.64 (d, J = 8.8 Hz, 1H), 7.10 (dd, J = 8.8 Hz, J = 2.5 Hz, 1H), 6.71 (d, J = 8.1 Hz, 1H), 6.10 (d, J = 5.1 Hz, 1H), 4.18 (t, J = 5.2 Hz, 1H), 4.10–4.03 (m, 3H), 3.98–3.94 (m, 1H), 3.72 (dd, J = 12.0 Hz, J = 3.0 3.64 (dd, J = 12.0 Hz, J = 3.3 1.81–1.70 (m, 2H), 1.55–1.42 (m, 2H), 0.96 (t, J = 7.4 Hz, 3H). ^13^C NMR (75 MHz, DMSO-d_6_): δ 155.39, 147.36, 146.98, 137.63, 131.78, 130.33, 119.07, 114.78, 99.06, 97.84, 88.55, 85.05, 74.20, 69.74, 67.78, 60.65, 30.71, 18.70, 13.67. HRMS (ESI) *m*/*z*: calcd for C_40_H_40_N_3_O_8_^−^ [M-H]^−^: 690.2821; found 690.2790.

**2-((2R,3R,4S,5R)-5-((bis(4-methoxyphenyl)(phenyl)methoxy)methyl)-3,4-dihydroxytetrahydrofuran-2-yl)-7-(hexyloxy)benzo[4,5]imidazo[1,2-c]pyrimidin-1(2H)-one 5b**

Yield 71%. ^1^H NMR (300 MHz, DMSO-d_6_): δ 7.91 (d, J = 8.1 Hz, 1H), 7.86 (d, J = 2.4 Hz, 1H), 7.63 (d, J = 8.8 Hz, 1H), 7.09 (dd, J = 8.8 Hz, J = 2.4 Hz, 1H), 6.71 (d, J = 8.1 Hz, 1H), 6.10 (d, J = 5.1 Hz, 1H), 4.17 (t, J = 5.0 Hz, 1H), 4.09–4.01 (m, 3H), 3.97–3.93 (m, 1H), 3.72 (dd, J = 12.2 Hz, J = 3.1 3.63 (dd, J = 12.2 Hz, J = 3.1 1.82–1.70 (m, 2H), 1.51–1.40 (m, 2H), 1.39–1.27 (m, 4H), 0.89 (t, J = 7.0 Hz, 3H). ^13^C NMR (75 MHz, DMSO-d_6_): δ 155.33, 147.38, 147.06, 138.10, 131.49, 130.41, 119.24, 114.68, 99.04, 98.06, 88.46, 85.07, 74.17, 69.80, 68.07, 60.70, 30.96, 28.61, 25.14, 22.02, 13.86. HRMS (ESI) *m*/*z*: calcd for C_42_H_44_N_3_O_8_^−^ [M-H]^−^: 718.3134; found 718.3129.

**2-((2R,3R,4S,5R)-5-((bis(4-methoxyphenyl)(phenyl)methoxy)methyl)-3,4-dihydroxytetrahydrofuran-2-yl)-7-(octyloxy)benzo[4,5]imidazo[1,2-c]pyrimidin-1(2H)-one 5c**

Yield 78%. ^1^H NMR (300 MHz, DMSO-d_6_): δ 7.93 (d, J = 8.1 Hz, 1H), 7.87 (d, J = 2.1 Hz, 1H), 7.64 (d, J = 8.9 Hz, 1H), 7.10 (dd, J = 8.9 Hz, J = 2.1 Hz, 1H), 6.72 (d, J = 8.1 Hz, 1H), 6.10 (d, J = 5.1 Hz, 1H), 4.18 (t, J = 5.2 Hz, 1H), 4.10–4.01 (m, 3H), 3.99–3.93 (m, 1H), 3.72 (dd, J = 11.9 Hz, J = 2.7 3.64 (dd, J = 11.9 Hz, J = 3.0 1.82–1.70 (m, 2H), 1.52–1.38 (m, 2H), 1.37–1.16 (m, 8H), 0.87 (t, J = 6.5 Hz, 3H). ^13^C NMR (75 MHz, DMSO-d_6_): δ 155.41, 147.35, 146.95, 137.49, 131.87, 130.30, 119.02, 114.79, 99.07, 97.77, 88.57, 85.06, 74.20, 69.73, 68.08, 60.64, 31.17, 28.70, 28.61 (2C), 25.47, 22.02, 13.90. HRMS (ESI) *m*/*z*: calcd for C_44_H_48_N_3_O_8_^−^ [M-H]^−^: 746.3447; found 746.3387.

**2-((2R,3R,4S,5R)-5-((bis(4-methoxyphenyl)(phenyl)methoxy)methyl)-3,4-dihydroxytetrahydrofuran-2-yl)-7-(decyloxy)benzo[4,5]imidazo[1,2-c]pyrimidin-1(2H)-one 5d**

Yield 75%. ^1^H NMR (300 MHz, DMSO-d_6_): δ 7.92 (d, J = 8.1 Hz, 1H), 7.86 (d, J = 2.5 Hz, 1H), 7.63 (d, J = 8.9 Hz, 1H), 7.09 (dd, J = 8.9 Hz, J = 2.5 Hz, 1H), 6.71 (d, J = 8.1 Hz, 1H), 6.10 (d, J = 5.1 Hz, 1H), 4.18 (t, J = 5.0 Hz, 1H), 4.10–4.02 (m, 3H), 3.98–3.94 (m, 1H), 3.72 (dd, J = 12.0 Hz, J = 3.0 3.63 (dd, J = 12.0 Hz, J = 3.2 1.81–1.71 (m, 2H), 1.51–1.39 (m, 2H), 1.34–1.21 (m, 12H), 0.85 (t, J = 6.8 Hz, 3H). ^13^C NMR (75 MHz, DMSO-d_6_): δ 155.39, 147.35, 146.97, 137.64, 131.77, 130.33, 119.07, 114.75, 99.07, 97.84, 88.54, 85.05, 74.19, 69.74, 68.07, 60.65, 31.22, 28.94, 28.89, 28.72, 28.63 (2C), 25.45, 22.02, 13.88. HRMS (ESI) *m*/*z*: calcd for C_46_H_52_N_3_O_8_^−^ [M-H]^−^: 774.3760; found 774.3751.

**2-((2R,3R,4S,5R)-5-((bis(4-methoxyphenyl)(phenyl)methoxy)methyl)-3,4-dihydroxytetrahydrofuran-2-yl)-7-(dodecyloxy)benzo[4,5]imidazo[1,2-c]pyrimidin-1(2H)-one 5e**

Yield 76%. ^1^H NMR (300 MHz, DMSO-d_6_): δ 7.90 (d, J = 8.0 Hz, 1H), 7.86 (d, J = 2.4 Hz, 1H), 7.63 (d, J = 9.0 Hz, 1H), 7.09 (dd, J = 8.9 Hz, J = 2.4 Hz, 1H), 6.70 (d, J = 8.0 Hz, 1H), 6.10 (d, J = 4.9 Hz, 1H), 4.17 (t, J = 5.1 Hz, 1H), 4.11–4.00 (m, 3H), 3.98–3.93 (m, 1H), 3.72 (dd, J = 12.0 Hz, J = 3.2 3.63 (dd, J = 12.0 Hz, J = 3.2 1.82–1.69 (m, 2H), 1.52–1.36 (m, 2H), 1.38–1.13 (m, 16H), 0.85 (t, J = 6.5 Hz, 3H). ^13^C NMR (75 MHz, DMSO-d_6_): δ 155.36, 147.37, 147.01, 137.85, 131.65, 130.37, 119.15, 114.72, 99.07, 97.95, 88.50, 85.06, 74.18, 69.76, 68.06, 60.67, 31.22, 28.92 (4C), 28.69, 28.63 (2C), 25.43, 22.02, 13.88. HRMS (ESI) *m*/*z*: calcd for C_48_H_56_N_3_O_8_^−^ [M-H]^−^: 802.4073; found 802.4049.

**General procedure for the preparation of 7-O-alkylated non-nucleoside derivatives**

Compound **4** (0.07 mmol) was dissolved in a mixture of CH_3_OH/12 N HCl (5 mL, 1:20, *v*:*v*) and kept at 50 °C for 72 h. The reaction mixture was concentrated, and the residue was purified using column chromatography on silica gel (0–5% CH_3_OH in CH_2_Cl_2_), yielding **6a**–**e** as a beige amorphous solid.

**7-butoxybenzo[4,5]imidazo[1,2-c]pyrimidin-1(2H)-one 6a**

Yield 89%. ^1^H NMR (300 MHz, DMSO-d_6_): δ 12.59 (br s, 1H), 7.90 (d, J = 2.4 Hz, 1H), 7.80 (d, J = 7.4 Hz, 1H), 7.72 (d, J = 8.9 Hz, 1H), 7.24 (dd, J = 8.9 Hz, J = 2.4 Hz, 1H), 6.79 (d, J = 7.4 Hz, 1H), 4.08 (t, J = 6.4 Hz, 1H), 1.81–1.70 (m, 2H), 1.55–1.41 (m, 2H), 0.95 (t, J = 7.4 Hz, 3H). ^13^C NMR (75 MHz, DMSO-d_6_): δ 156.14, 148.07, 146.03, 138.55, 129.26, 128.75, 116.06, 115.88, 99.53, 93.15, 68.00, 30.50, 18.52, 13.48. HRMS (ESI) *m*/*z*: calcd for C_14_H_14_N_3_O_2_^−^ [M-H]^−^: 256.1092; found 256.1075.

**7-(hexyloxy)benzo[4,5]imidazo[1,2-c]pyrimidin-1(2H)-one 6b**

Yield 86%. ^1^H NMR (300 MHz, DMSO-d_6_): δ 12.55 (br s, 1H), 7.90 (d, J = 2.4 Hz, 1H), 7.78 (d, J = 7.4 Hz, 1H), 7.72 (d, J = 8.9 Hz, 1H), 7.23 (dd, J = 8.9 Hz, J = 2.4 Hz, 1H), 6.78 (d, J = 7.4 Hz, 1H), 4.07 (t, J = 6.4 Hz, 1H), 1.82–1.71 (m, 2H), 1.51–1.40 (m, 2H), 1.40–1.27 (m, 4H), 0.89 (t, J = 6.9 Hz, 3H). ^13^C NMR (75 MHz, DMSO-d_6_): δ 156.13, 148.09, 146.06, 138.49, 129.36, 128.77, 116.05, 115.92, 99.52, 93.20, 68.30, 30.79, 28.39, 24.94, 21.84, 13.68. HRMS (ESI) *m*/*z*: calcd for C_16_H_18_N_3_O_2_^−^ [M-H]^−^: 284.1405; found 284.1388.

**7-(octyloxy)benzo[4,5]imidazo[1,2-c]pyrimidin-1(2H)-one 6c**

Yield 94%. ^1^H NMR (600 MHz, DMSO-d_6_): δ 12.52 (br s, 1H), 7.89 (d, J = 2.4 Hz, 1H), 7.78 (d, J = 7.4 Hz, 1H), 7.72 (d, J = 8.9 Hz, 1H), 7.23 (dd, J = 8.9 Hz, J = 2.4 Hz, 1H), 6.78 (d, J = 7.4 Hz, 1H), 4.06 (t, J = 6.4 Hz, 1H), 1.79–1.73 (m, 2H), 1.48–1.41 (m, 2H), 1.35–1.23 (m, 8H), 0.86 (t, J = 6.9 Hz, 3H). ^13^C NMR (150 MHz, DMSO-d_6_): δ 156.11, 148.15, 146.19, 138.30, 129.73, 128.86, 116.11, 116.03, 99.46, 93.42, 68.28, 31.09, 28.60, 28.51, 28.48, 25.35, 21.93, 13.80. HRMS (ESI) *m*/*z*: calcd for C_18_H_22_N_3_O_2_^−^ [M-H]^−^: 312.1718; found 312.1701.

**7-(decyloxy)benzo[4,5]imidazo[1,2-c]pyrimidin-1(2H)-one 6d**

Yield 94%. ^1^H NMR (300 MHz, DMSO-d_6_): δ 12.18 (br s, 1H), 7.89 (d, J = 2.4 Hz, 1H), 7.69 (d, J = 8.9 Hz, 1H), 7.66 (d, J = 7.6 Hz, 1H), 7.19 (dd, J = 8.9 Hz, J = 2.4 Hz, 1H), 6.72 (d, J = 7.6 Hz, 1H), 4.07 (t, J = 6.5 Hz, 1H), 1.82–1.71 (m, 2H), 1.51–1.39 (m, 2H), 1.37–1.21 (m, 12H), 0.86 (t, J = 6.8 Hz, 3H). ^13^C NMR (75 MHz, DMSO-d_6_): δ 156.04, 148.19, 146.28, 137.81, 130.45, 128.96, 116.35, 115.90, 99.49, 93.71, 68.29, 31.09, 28.79, 28.74, 28.56, 28.48, 28.45, 25.29, 21.89, 13.74. HRMS (ESI) *m*/*z*: calcd for C_20_H_26_N_3_O_2_^−^ [M-H]^−^: 340.2031; found 340.1987.

**7-(dodecyloxy)benzo[4,5]imidazo[1,2-c]pyrimidin-1(2H)-one 6e**

Yield 91%. ^1^H NMR (300 MHz, DMSO-d_6_): δ 12.58 (br s, 1H), 7.90 (d, J = 1.7 Hz, 1H), 7.82 (d, J = 7.2 Hz, 1H), 7.73 (d, J = 8.9 Hz, 1H), 7.24 (dd, J = 8.9 Hz, J = 1.7 Hz, 1H), 6.81 (d, J = 7.2 Hz, 1H), 4.07 (t, J = 6.3 Hz, 1H), 1.79–1.73 (m, 2H), 1.47–1.41 (m, 2H), 1.35–1.20 (m, 4H), 0.85 (t, J = 6.8 Hz, 3H). ^13^C NMR (75 MHz, DMSO-d_6_): δ 219.47, 156.29, 148.15, 146.09, 139.04, 139.00, 128.77, 116.27, 115.85, 99.56, 93.08, 68.34, 31.17, 28.91, 28.88, 28.85, 28.63, 28.58, 28.47, 25.34, 21.97, 13.83. HRMS (ESI) *m*/*z*: calcd for C_22_H_30_N_3_O_2_^−^ [M-H]^−^: 368.2344; found 368.2339.

**General procedure for the preparation of 7-O-methyl 5′-O-DMTr nucleoside derivatives**

To a stirred mixture of compound **3** (0.11 mmol), PPh_3_ (58 mg, 0.22 mmol, 2 equiv) and CH_3_OH (6 μL, 0.15 mmol, 1.3 equiv) in dry CH_2_Cl_2_ (5 mL) DIAD (43 μL, 0.22 mmol, 2 equiv) was added at 0 °C. The reaction mixture was stirred at RT overnight and concentrated under reduced pressure. The residue was purified using column chromatography on silica gel (0–1% CH_3_OH in CH_2_Cl_2_ with 0.1% TEA), yielding **7a**,**b** as a yellowish amorphous solid.

**2-((2R,4S,5R)-5-((bis(4-methoxyphenyl)(phenyl)methoxy)methyl)-4-hydroxytetrahydrofuran-2-yl)-7-methoxybenzo[4,5]imidazo[1,2-c]pyrimidin-1(2H)-one 7a**

Yield 83%. ^1^H NMR (600 MHz, DMSO-d_6_): δ 7.86 (d, J = 2.5 Hz, 1H), 7.65 (d, J = 8.0 Hz, 1H), 7.64 (d, J = 8.8 Hz, 1H), 7.42–7.39 (m, 2H), 7.33–7.21 (m, 7H), 7.10 (dd, J = 8.8 Hz, J = 2.5 Hz, 1H), 6.91–6.87 (m, 4H), 6.45 (t, J = 6.4 Hz, 1H), 6.42 (d, J = 8.0 Hz, 1H), 5.43 (br s, 1H), 4.41–4.36 (m, 1H), 3.99–3.96 (m, 1H), 3.85 (s, 3H), 3.72 (s, 3H), 3.72 (s, 3H), 3.31 (dd, J = 10.4 Hz, J = 4.9 Hz, 1H), 3.27 (dd, J = 10.4 Hz, J = 3.0 Hz, 1H), 2.39–2.30 (m 2H). ^13^C NMR (150 MHz, DMSO-d_6_): δ 158.00 (2C), 155.90, 147.27, 146.58, 144.57, 138.20, 135.34, 135.15, 131.16, 130.38, 129.65 (4C), 127.75 (2C), 127.63 (2C), 126.63, 119.19, 114.16, 113.11 (4C), 98.36, 97.79, 85.74, 85.54, 84.98, 69.74, 63.21, 55.54, 54.90 (2C), 39.94. HRMS (ESI) *m*/*z*: calcd for C_37_H_34_N_3_O_7_^−^ [M-H]^−^: 632.2402; found 632.2157.

**2-((2R,3R,4S,5R)-5-((bis(4-methoxyphenyl)(phenyl)methoxy)methyl)-3,4-dihydroxytetrahydrofuran-2-yl)-7-methoxybenzo[4,5]imidazo[1,2-c]pyrimidin-1(2H)-one 7b**

Yield 80%. ^1^H NMR (600 MHz, DMSO-d_6_): δ 7.87 (d, J = 2.5 Hz, 1H), 7.74 (d, J = 8.0 Hz, 1H), 7.65 (d, J = 8.8 Hz, 1H), 7.44–7.41 (m, 2H), 7.35–7.23 (m, 7H), 7.11 (dd, J = 8.8 Hz, J = 2.5 Hz, 1H), 6.93–6.89 (m, 4H), 6.34 (d, J = 8.0 Hz, 1H), 6.07 (d, J = 3.5 Hz, 1H), 5.60 (d, J = 4.6 Hz, 1H), 5.22 (d, J = 5.7 Hz, 1H), 4.27–4.23 (m, 1H), 4.24–4.20 (m, 1H), 4.09–4.06 (m, 1H), 3.85 (s, 3H), 3.74 (s, 6H), 3.37–3.31 (m, 2H). ^13^C NMR (150 MHz, DMSO-d_6_): δ 158.05 (2C), 155.94, 147.23, 146.73, 144.56, 138.17, 135.31, 135.08, 132.96, 132.28, 129.69 (4C), 127.80 (2C), 127.65 (2C), 126.69, 119.22, 114.20, 113.16 (4C), 98.35, 97.65, 89.72, 85.84, 82.53, 73.89, 69.38, 62.69, 55.53, 54.93, 54.76. HRMS (ESI) *m*/*z*: calcd for C_37_H_34_N_3_O_8_^−^ [M-H]^−^: 648.2351; found 648.2302.

**General procedure for the preparation of 7-O-methyl nucleoside derivatives**

Compound 7 (0.6 mmol) was dissolved in a mixture of CH_3_COOH/H_2_O (2 mL, 1:9, *v*:*v*) and kept at 50 °C for 8 h. After concentration and co-evaporation with toluene (3 × 10 mL), the residue was purified using column chromatography on silica gel (0–6% CH_3_OH in CH_2_Cl_2_), yielding **8a**,**b** as a beige amorphous solid.

**2-((2R,4S,5R)-4-hydroxy-5-(hydroxymethyl)tetrahydrofuran-2-yl)-7-methoxybenzo[4,5]imidazo[1,2-c]pyrimidin-1(2H)-one 8a**

Yield 91%. ^1^H NMR (300 MHz, DMSO-d_6_): δ 7.88 (s, 1H), 7.85 (d, J = 2.4 Hz, 1H), 7.63 (d, J = 8.8 Hz, 1H), 7.08 (dd, J = 8.8 Hz, J = 2.4 Hz, 1H), 6.70 (d, J = 8.0 Hz, 1H), 6.46 (t, J = 6.4 Hz, 1H), 5.35 (d, J = 3.6 Hz, 1H), 5.13 (t, J = 4.6 Hz, 1H), 4.36–4.27 (m, 1H), 3.91–3.83 (m, 1H), 3.84 (s, 3H), 3.70–3.57 (m, 2H), 2.30–2.18 (m, 2H). ^13^C NMR (75 MHz, DMSO-d_6_): δ 155.90, 147.49, 146.78, 138.24, 131.35, 130.43, 119.26, 114.23, 98.35, 98.13, 87.68, 84.92, 70.29, 61.19, 55.58, 40.26. HRMS (ESI) *m*/*z*: calcd for C_16_H_16_N_3_O_5_^−^ [M-H]^−^: 330.1095; found 330.1074.

**2-((2R,3R,4S,5R)-3,4-dihydroxy-5-(hydroxymethyl)tetrahydrofuran-2-yl)-7-methoxybenzo[4,5]imidazo[1,2-c]pyrimidin-1(2H)-one 8b**

Yield 92%. ^1^H NMR (300 MHz, DMSO-d_6_): δ 7.93 (d, J = 7.9 Hz, 1H), 7.86 (s, 1H), 7.65 (d, J = 7.9 Hz, 1H), 7.10 (d, J = 7.9 Hz, 1H), 6.72 (d, J = 7.9 Hz, 1H), 6.10 (d, J = 4.1 Hz, 1H), 5.50 (d, J = 4.6 Hz, 1H), 5.23 (t, J = 4.2 Hz, 1H), 5.19 (d, J = 4.0 Hz, 1H), 4.22–4.10 (m, 1H), 4.11–4.06 (m, 1H), 3.99–3.89 (m, 1H), 3.85 (s, 3H), 3.76–3.57 (m, 2H). ^13^C NMR (75 MHz, DMSO-d_6_): δ 155.94, 147.44, 147.08, 138.22, 131.50, 130.45, 119.30, 114.25, 98.33, 98.10, 88.50, 85.08, 74.22, 69.80, 60.70, 55.57. HRMS (ESI) *m*/*z*: calcd for C_16_H_16_N_3_O_6_^−^ [M-H]^−^: 346.1045; found 346.1012.

### 3.2. Biology

#### 3.2.1. Cells and Viruses

Human embryonic lung fibroblasts (HEL299), human hepatoma (Huh7), green monkey kidney (Vero), and Madin-Darby canine kidney (MDCK) cell lines were obtained from the American Type Culture Collection (ATCC). Viruses and strains used in the present work, with the exception of SARS-CoV-2 strains, as well as the specific cell type for each virus, are specified in Table S1.

A variant of SARS-CoV-2, designated UC-1074, was isolated in Vero cells in 2020 from a nasopharyngeal swab of a COVID-19 patient who had a Ct of 19 from the detection of SARS-CoV-2 E protein using RT-qPCR. UC-1074 has a genome sequence matching the early A lineage (Wuhan/WH04/2020). The following four strains were kindly provided by Piet Maes (Laboratory of Clinical and Epidemiological Virology, Rega Institute, KU Leuven, Belgium): NVDBB-2220 (Alpha variant), RG-2674 (Beta variant), 860-J1 (Delta variant), and B1.1.529 BA.1 (Omicron). All studies on SARS-CoV-2 were conducted in the BSL3 facilities of the KU Leuven Rega Institute (3CAPS) under licenses AMV 30112018 SBB 219 2018 0892 and AMV 23102017 SBB 219 2017 0589 according to institutional guidelines.

#### 3.2.2. Methods

##### Cell Toxicity Evaluation in HEL299, Vero, Huh7 and MDCK Cells

Cells were seeded at a rate of 5 × 10^3^ cells/well into 96-well plates and allowed to proliferate for 24 h. Then, a medium containing different concentrations of the test compounds, starting at 100 mM, was added. After 3 days of incubation at 37 °C, the cell count was determined with a Beckman Coulter counter or, alternatively, cell viability was determined with the colorimetric formazan-based MTS assay. The compound concentration required to reduce cell growth by 50% (CC_50_) was estimated from graphical plots of the number of cells (percentage of control) as a function of the concentration of the test compounds. The cytotoxic effects of the compounds were also assessed by evaluating the MCC (minimum cytotoxic concentration that causes a microscopically detectable alteration of cell morphology).

##### Cytopathicity or Plaque Reduction Test

Confluent cell cultures in microtiter 96-well plates were inoculated with 100 CCID_50_ of virus (1 CCID_50_ being the virus dose to infect 50% of the cell cultures) or with 20 or 100 plaque-forming units (PFU) for VZV and HCMV, respectively. Following a 2 h adsorption period, viral inoculum was removed and the cell cultures were incubated in the presence of varying concentrations of the test compounds. Viral cytopathicity, or plaque formation, was recorded as soon as it reached completion in the control virus-infected cell cultures that were not treated with the test compounds. Antiviral activity was expressed as EC_50_ (the compound concentration required to reduce virus-induced cytopathicity or viral plaque formation by 50%).

### 3.3. Biochemical Assays

#### 3.3.1. RdRp and Reagents

SARS-CoV-2 RdRp containing the catalytic subunit nsp12 and fused nsp7-nsp8 subunits (separated by a His6-tag) was expressed in Escherichia coli BL-21(DE3) and purified using Ni-affinity and HiTrap Q HP columns as described previously [41]. RNA oligonucleotides for in vitro reactions were synthesized and purified by DNA Synthesis (Moscow, Russia). All reagents for protein purification and in vitro experiments were from Sigma-Aldrich (St. Louis, MO, USA) unless otherwise specified. The inhibitors were diluted in DMSO (VWR International LLC, Radnor, PA, USA) and stored at −20 °C.

#### 3.3.2. In Vitro Transcription Assay

The primer RNA oligonucleotide was 5′-labeled with γ-[^32^P]-ATP by T4 polynucleotide kinase (NEB, Ipswich, MA, USA). The RNA substrate was prepared by annealing the labeled primer with the template RNA oligonucleotide as described previously [41]. To test the effects of the studied compounds on RdRp activity, RdRp (1 µM) was incubated with increasing concentrations of the inhibitors (1–10,000 μM, or the same volume of DMSO in control samples) in a reaction buffer (10 mM Tris-HCl, pH 7.9, 10 mM KCl, 2 mM MgCl_2_, and 1 mM DTT) for 10 min at 30 °C. Then, the RNA substrate (25 nM) was added and the samples were incubated for 10 min at 30 °C. RNA synthesis was initiated by adding 100 µM of all 4 NTPs (Thermo Fisher Scientific, Waltham, MA, USA) and was quenched after 10 min with a 1.2 volume of the stop solution containing 95% formamide, 10 mM EDTA, heparin 100 μg/mL. The samples were heated at 98 °C for 3 min. The reaction products were separated by 15% denaturing PAGE (acrylamide:bisacrylaimde 19:1, 8 M urea) and visualized by phosphor imaging using a Typhoon 9500 scanner (GE Healthcare, Chicago, IL, USA). The efficiency of primer extension was calculated in every sample and normalized to activity in the absence of the inhibitors. The data were fitted to the hyperbolic equation A = A_max_ × (1 − [Inhibitor]/(IC_50_ + [Inhibitor])), where A is the efficiency of the RNA extension at a given concentration of the inhibitor and A_max_ is the maximal activity, using GraFit software version 4 (Erithacus). All experiments were independently reproduced 3 times.

#### 3.3.3. LLPS Assays

Recombinant SARS-CoV-2 N-protein with His-tag, obtained as described previously [27] (purity: ≥90%), was a kind gift of the group of Prof. V. N. Lazarev. The protein was labeled with a far-red-emitting RED dye using the RED-NHS 2nd Generation Protein Labeling Kit (Nanotemper, Munich, Germany). The stem-loop fragment of SARS-CoV-2 gRNA (SL5 in [43]) was obtained by in vitro transcription from the T7-promoter-containing dsDNA template according to the published procedure [27].

Template sense strand:
TAATACGACTCACTATAGGGAGAACTAATTACTGTCGTTGACAGGACACGAGTAACTCGTCTATCTTCTGCAGGCTGCTTACGGTTTCGTCCGTGTTGCAGCCGATCATCAGCACATCTAGGTTTCGTCCGGGTGTGACCGAAAGGTAAGATGGAGAGCCTTGTCCCTGGTTTCAACGACAGTAATTAGT

The labeled N-protein (3 µM final concentration) was mixed with RNA (6 µM final concentration) in the RNAse-free 20 mM sodium phosphate buffer (pH 6) supplemented with 150 mM NaCl. Compound **3a**/**3b**/**7a**/**9a** was added to a final concentration of 20 µM. The mixture was incubated at 37 °C for 2 h. Then, 3 μL of the mixture was placed between glass slides and analyzed using a Nikon Eclipse Ti2 microscope (Nikon, Tokyo, Japan) in the red channel. All experiments were performed in 6 repeats. Droplets were counted in ImageJ software, version 1.53a, and the partitioning coefficient (PC = [N-protein]_droplet_/[N-protein]_solution_) was calculated based on the average fluorescence intensity values in droplets and bulk solution.

## 4. Conclusions

Using the reaction between cytosine and 1,4-benzoquinone to assemble a tricyclic heteroaromatic backbone, a set of nucleoside and non-nucleoside derivatives substituted at the 7-hydroxyl position with alkyl groups of varying length was obtained and then tested for their ability to inhibit the replication of a wide range of DNA and RNA viruses. Among the synthesized compounds, only 5′-O-DMTr-substituted intermediates (**3a** and **3b)** showed micromolar to submicromolar activity against human coronavirus and respiratory syncytial virus without any noticeable cytotoxicity for the host cells (HEL299) (Table 1). Then, a few analogs of **3a**,**b** were synthesized, among which derivatives **7a**,**b** which additionally contained a methyl group at the seventh position of the heteroaromatic scaffold exhibited micromolar anti-VZV activity, with SI being more than 30 for **7a** (Table 2). Finally, **3a**,**b** and their analogs were tested against various SARS-CoV-2 strains, with **3a** showing an EC_50_ of about 10 μM against all strains, however, with a moderate effect on Vero cell morphology. Since **3a** belongs to nucleoside analogs, which often exhibit their activity by the inhibition of viral polymerases, we tested its ability to inhibit SARS-CoV-2 RdRp in vitro. The compound was a transcription inhibitor with an IC_50_ value of 94 ± 3 μM, which, however, was one order of magnitude higher than the EC_50_ value for the same compound, suggesting RdRp may not be the primary target. One possible additional pathway, namely interference with the replication-promoting biocondensate assembly, was verified for **3a** and its structurally related inactive analogs in LLPS assays. Of all tested compounds, only **3a** decreased condensate formation in a dose-dependent manner with EC_50_ ≤ 10 µM. The results confirm the promise and pave the way for further development of LLPS inhibitors as antiviral compounds.

## Figures and Tables

**Table 1:** Activity and cytotoxicity of derivatives from the first synthetic round against HCoV (229E, OC43, and NL63) and RSV (Long) in HEL299/Huh7 cells.

Cmpd #	Antiviral Activity (EC_50_, μM) ^a^				Cytotoxicity (CC_50_, μM) ^b^	
	HCoV			RSV	HEL299	Huh7
	229E	OC43	NL63	Long		
**2a**	>100	>100	>100	>100	>100	>100
**2b**	>100	>100	>100	>100	>100	>100
**3a**	8.5 ± 0.36	<0.8	>100	0.67 ± 0.23	>100	<0.8
**3b**	4.1 ± 2.0	<0.8	>100	5.7 ± 0.8	>20	<0.8
**4a**	>100	>100	>100	>100	>40	>40
**4b**	>100	>100	>100	>100	>100	>100
**4c**	>100	>100	>100	>100	>40	>100
**4d**	>100	>100	>100	>100	>40	>40
**4e**	>100	>100	>100	>100	>100	>100
**5a**	>100	>100	>100	>100	>100	>100
**5b**	>100	>50	>100	>100	>100	>10
**5c**	>100	>100	>100	>100	>100	>10
**5d**	>100	>100	>100	>100	>100	>100
**5e**	>100	>100	>100	>100	>100	>40
**6a**	>100	>100	>100	>100	>100	>100
**6b**	>100	>100	>100	>100	>100	>100
**6c**	>100	>100	>100	>100	>100	>100
**6d**	>100	>100	>100	>100	>100	>100
**6e**	>100	>100	>100	>100	>100	>100
Remdesivir	0.03	0.03	0.03	0.02	>10 ^c^	>10 ^c^

^a^ Effective concentration required to reduce the virus-induced cytopathic effect by 50%. Virus input was 100 TCID_50_ (median tissue culture infectious dose). ^b^ Cytotoxic concentration required to reduce cell growth by 50%, as determined by measuring cell viability with the colorimetric formazan-based MTS assay. ^c^ Remdesivir was not toxic up to the highest tested concentration (10 μM).

**Table 2:** Activity and cytotoxicity of derivatives from the second synthetic round against VZV in HEL299 cells.

Cmpd #	Antiviral Activity (EC_50_, μM) ^a^		Cytotoxicity	
	TK^+^ VZV Strain	TK^−^ VZV Strain	Cell Morphology	Cell Growth
	OKA	07-1	(MCC, μM) ^b^	(CC_50_, μM) ^c^
**3a**	>4	>4	20	ND ^d^
**3b**	>4	>4	20	ND
**7a**	8.15 ± 3.59	3.28 ± 2.23	100	>100
**7b**	30.7 ± 4.68	7.13 ± 0.94	≥100	10.3 ± 5.63
**8a**	>20	>20	100	ND
**8b**	>20	>20	100	ND
**9a**	>20	>20	100	ND
**9b**	>4	>20	≥20	ND
Aciclovir	6.97 ± 4.74	29.9 ± 13.4	>100	>100
Brivudine	0.10 ± 0.07	0.14 ± 0.03	>100	>100

^a^ Effective concentration required to reduce virus plaque formation by 50%. Virus input was 20 plaque-forming units (PFU). ^b^ Minimum cytotoxic concentration that causes a microscopically detectable alteration of cell morphology. ^c^ Cytotoxic concentration required to reduce cell growth by 50%, determined after 3 days of cell growth using a Coulter Counter. ^d^ Not determined.

**Table 3:** Activity and cytotoxicity of derivatives from the second synthetic round against SARS-CoV-2 in Vero cells.

Cmpd #	Antiviral Activity (EC_50_, μM) ^a^					Cytotoxicity	
	UC-1074 (Wuhan)	RG-2675 (South Africa)	NVDBB-2220 (UK)	860-J1 (Delta)	B1.1 529 BA.1 (Omicron)	Cell Morphology (MCC, μM) ^b^	Cell Growth (CC_50_, μM) ^c^
**3a**	8.82 ± 0.21	9.10 ± 0.62	8.92 ± 6.38	11.3 ± 3.5	10.6 ± 0.7	≥20	>100
**3b**	>20	ND ^d^	ND	ND	>20	20	ND
**7a**	>20	ND	ND	ND	>100	>100	ND
**7b**	>20	ND	ND	ND	>20	>100	ND
**8a**	>20	ND	ND	ND	>20	100	ND
**8b**	>20	ND	ND	ND	>20	100	ND
**9a**	>100	ND	ND	ND	>100	>100	ND
**9b**	>20	ND	ND	ND	>20	100	ND
Remdesivir	1.96 ± 1.00	1.31 ± 0.68	0.72 ± 0.15	3.23 ± 0.61	0.91 ± 0.17	≥40	≥40
Molnupiravir	5.52 ± 0.28	5.18 ± 0.26	3.08 ± 2.12	5.78 ± 0.29	5.20 ± 0.23	>100	>100

^a^ Effective concentration required to reduce virus-induced cytopathic effect by 50%. Virus input was 100 TCID_50_ (median tissue culture infectious dose). ^b^ Minimum cytotoxic concentration that causes a microscopically detectable alteration of cell morphology. ^c^ Cytotoxic concentration required to reduce cell growth by 50%, as determined by counting the cells using a Coulter Counter. ^d^ Not determined.

## Data Availability

The data presented in this study are available in the article and Supplementary Materials.

## Supplementary Materials

The following supporting information can be downloaded at: https://www.mdpi.com/article/10.3390/ijms241914540/s1.
