# Supplementary material for: Phenotypic Test of Benzo[4,5]imidazo[1,2-c]pyrimidinone-Based Nucleoside and Non-Nucleoside Derivatives against DNA and RNA Viruses, Including Coronaviruses
Source: Int J Mol Sci. 2023 Sep 26;24(19):14540. doi: 10.3390/ijms241914540 (PMC10572855; doi:10.3390/ijms241914540)

Phenotypic Test of Benzo[4,5]imidazo[1,2-c]pyrimidinone- Based Nucleoside and Non-Nucleoside Derivatives against DNA and RNA Viruses, including Coronaviruses

Polina Kamzeeva [1], Ivan Petushkov [2,3], Ekaterina Knizhnik [4,5], Robert Snoeck [6], Yuri Khodarovich [1], Ekaterina Ryabukhina [1], Vera Alferova [1], Artur Eshtukov-Shcheglov [1], Evgeny Belyaev [7], Julia Svetlova [4], Tatiana Vedekhina [4], Andrey Kulbachinskiy [2,3], Anna Varizhuk [4,5], Graciela Andrei [6] and Andrey Aralov [1,*]

Table S1. Viruses used in the present study for phenotypic test

Acronym/cells used	Virus (common name)	Species	Genus	Family	Strain(s)	Reference
dsDNA viruses						
HSV-1 HEL299	Herpes simplex virus 1	*Human alphaherpesvirus 1*	*Simplexvirus*	*Herpesviridae*	KOS	ATCC VR-1493
VZV HEL299	Varicella Zoster virus	*Human alphaherpesvirus 3*	*Varicellovirus*		TK$^+$ OKA and TK$^-$ 07-1	ATCC VR-795 and kindly provided by Shiro Shigeta, Fukushima Medical Center, Japan
HCMV HEL299	Cytomegalovirus	*Human betaherpesvirus 5*	*Cytomegalovirus*		AD-169 and Davis	ATCC VR-538 and VR-807
+ ssRNA viruses						
HCoV HEL299	*Human coronavirus*	*229E*	*Alphacoronavirus*	*Coronaviridae*	229E	ATCC VR-740
		NL63	*Alphacoronavirus*		NL63	ATCC-VR-3263SD
		OC43	*Betacoronavirus*		OC43	ATCC-VR-1558
SARS-CoV-2 Vero	Severe acute respiratory syndrome-related coronavirus 2	*Severe acute respiratory syndrome-related coronavirus*	*Betacoronavius*		UC-1074 (Wuhan) RG-2675 (South Africa) NVDBB-2220 (UK) 860-J1 (Delta) B1.1 529 BA.1 (Omicron)	See paragraph 4.2.1.
YFV Huh7	*Yellow fever virus*		*Flavivirus*	*Flaviviridae*	17D-204	Vaccine strain
-ssRNA viruses						
H1N1 MDCK	*Influenza A virus*		*Alphainfluenzavirus*	*Orthomyxoviridae*	A/Ned/378/05	Provided by R. Fouchier, Rotterdam, the Netherlands
					A/PR/8/34	ATCC VR-95
H3N2 MDCK					A/HK/7/87	Provided by R. Fouchier, Rotterdam, the Netherlands
B MDCK	*Influenza B virus*		*Betainfluenzavirus*		B/Ned/537/05	Provided by R. Fouchier, Rotterdam, the Netherlands
RSV HEL299	Respiratory syncytial virus	*Human orthopneumovirus*	*Orthopneumovirus*	*Pneumoviridae*	Long	ATCC VR-26

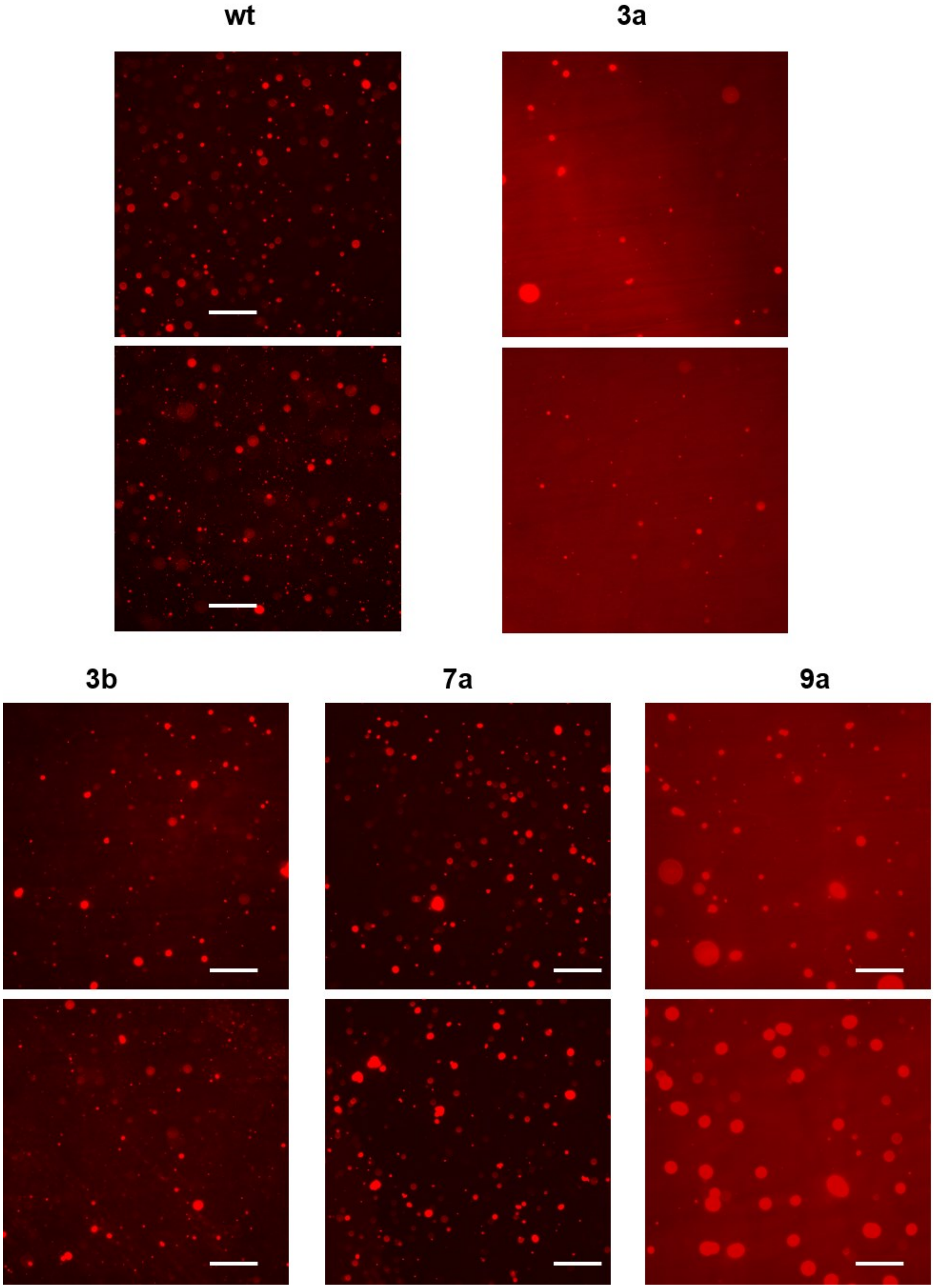

Figure S1. Representative examples of fluorescence microscopy images from LLPS assays. Conditions: 3 µM N-protein, 6 µM RNA, 20 mM sodium phosphate (pH 6) 150 mM NaCl. Scale bar: 10 µm.

NMR spectra

7-hydroxy-2-((2R,4S,5R)-4-hydroxy-5-(hydroxymethyl)tetrahydrofuran-2-yl)benzo[4,5]imidazo[1,2-c]pyrimidin-1(2H)-one **2a**

^{1}H NMR spectrum

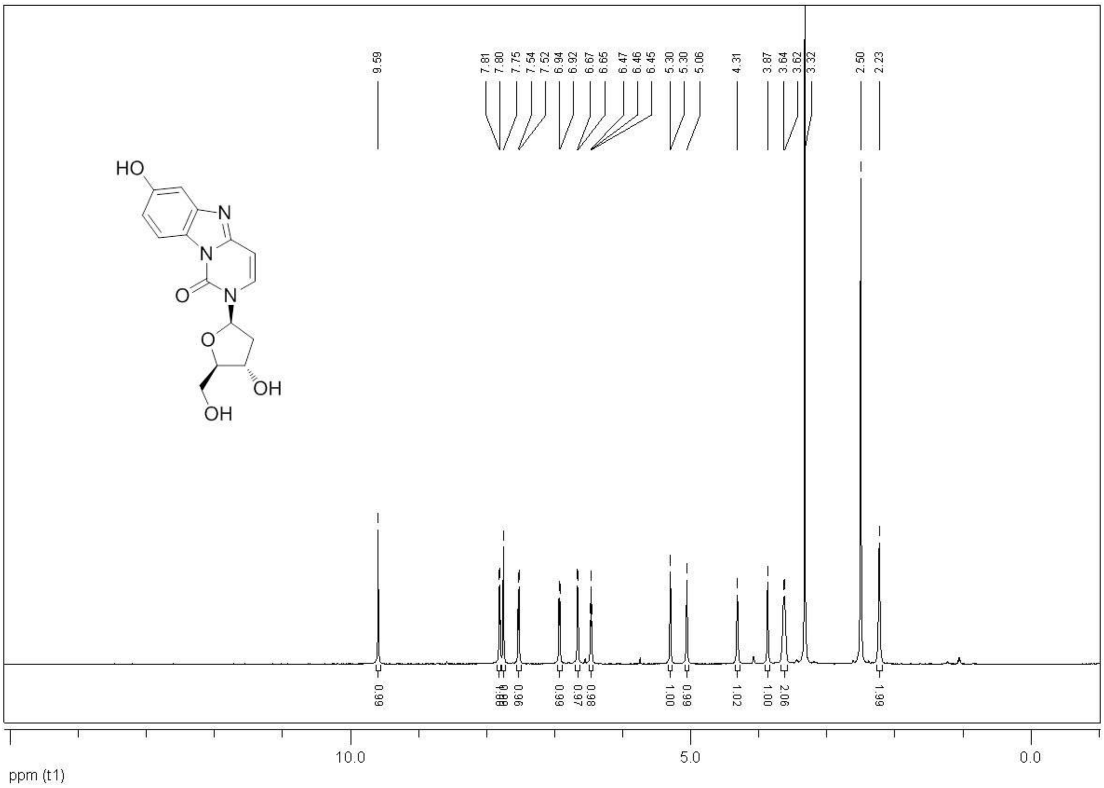

^{13}C NMR spectrum

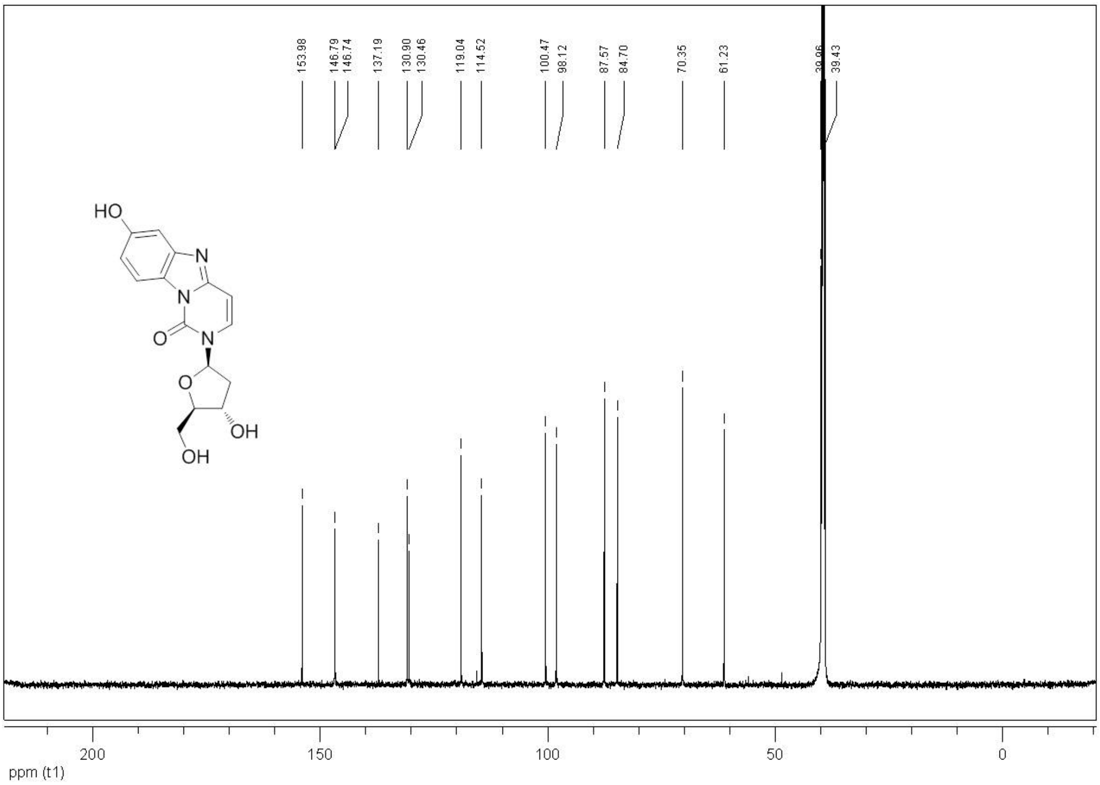

2-((2R,3R,4S,5R)-3,4-dihydroxy-5-(hydroxymethyl)tetrahydrofuran-2-yl)-7-hydroxybenzo[4,5]imidazo[1,2-c]pyrimidin-1(2H)-one **2b**

^{1}H NMR spectrum

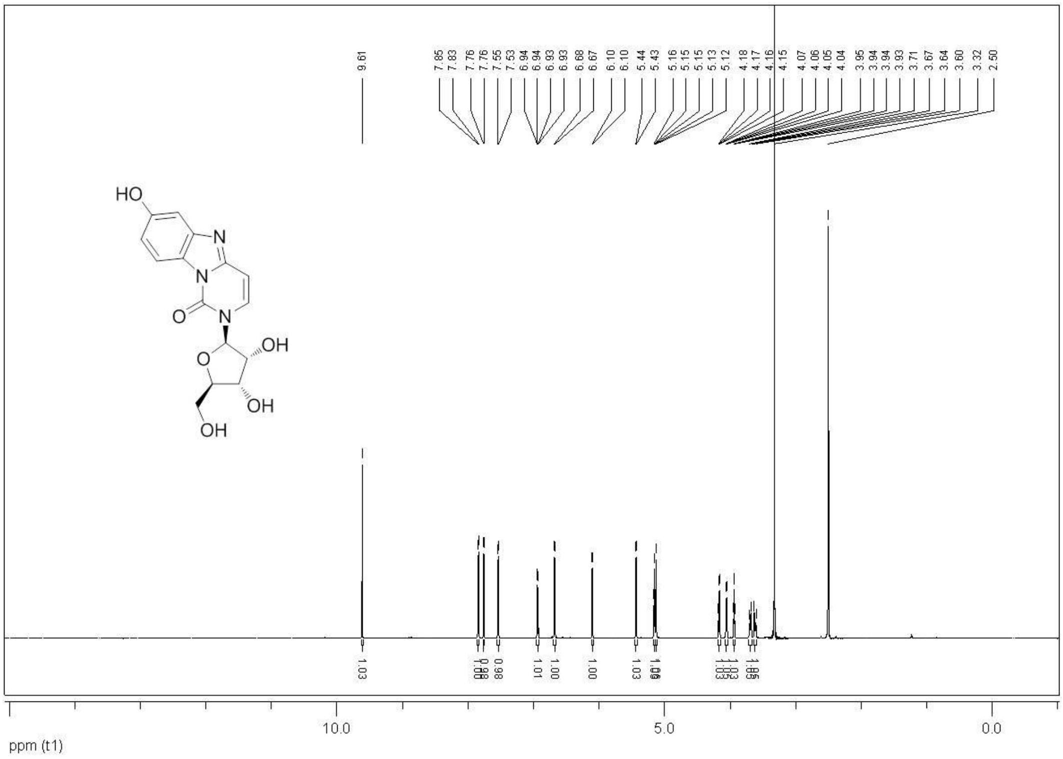

^{13}C NMR spectrum

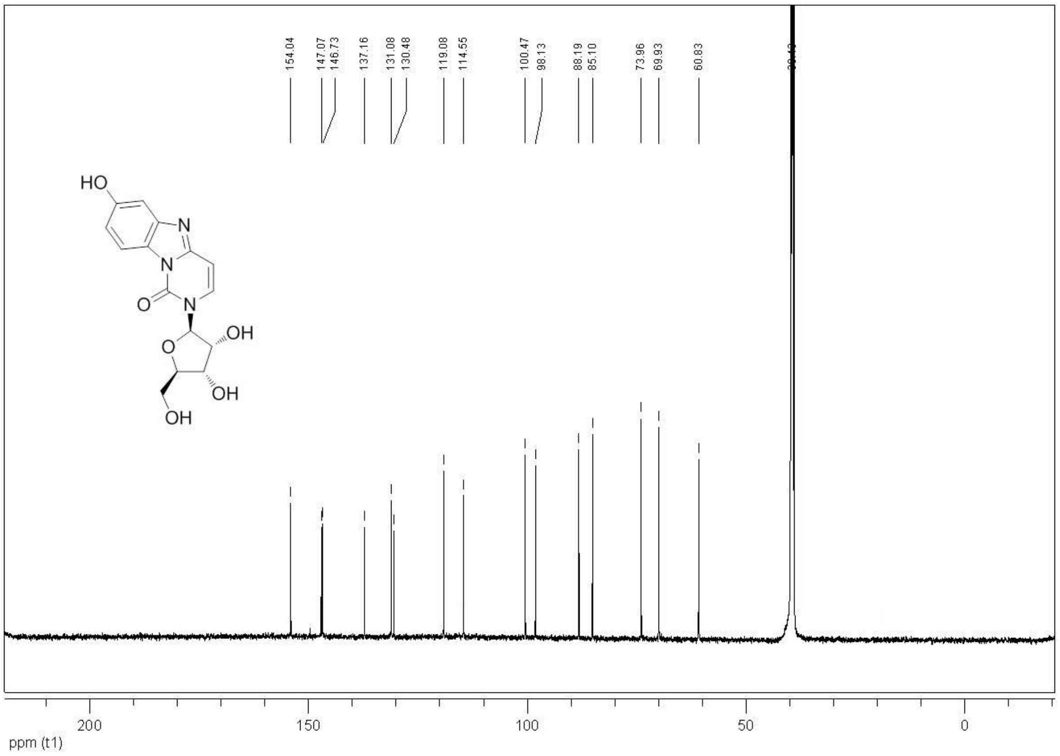

2-((2R,4S,5R)-5-((bis(4-methoxyphenyl)(phenyl)methoxy)methyl)-4-hydroxytetrahydrofuran-2-yl)-7-hydroxybenzo[4,5]imidazo[1,2-c]pyrimidin-1(2H)-one **3a**

^{1}H NMR spectrum

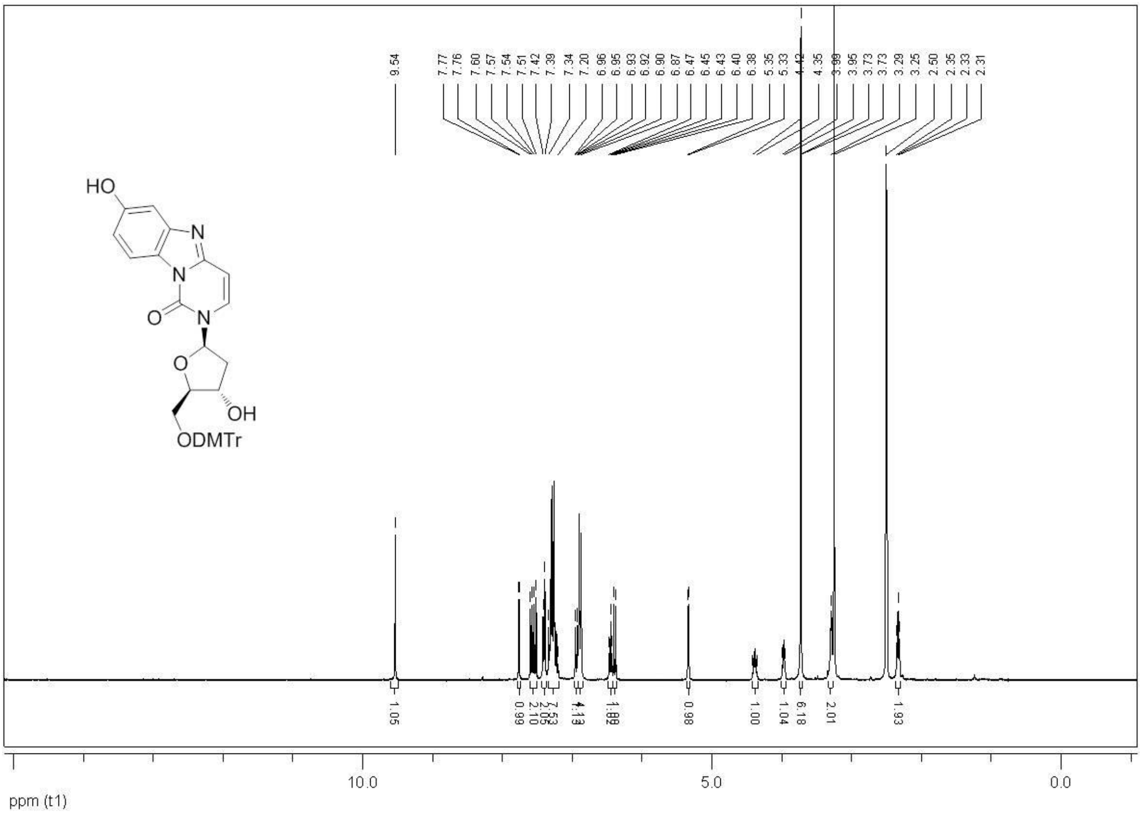

^{13}C NMR spectrum

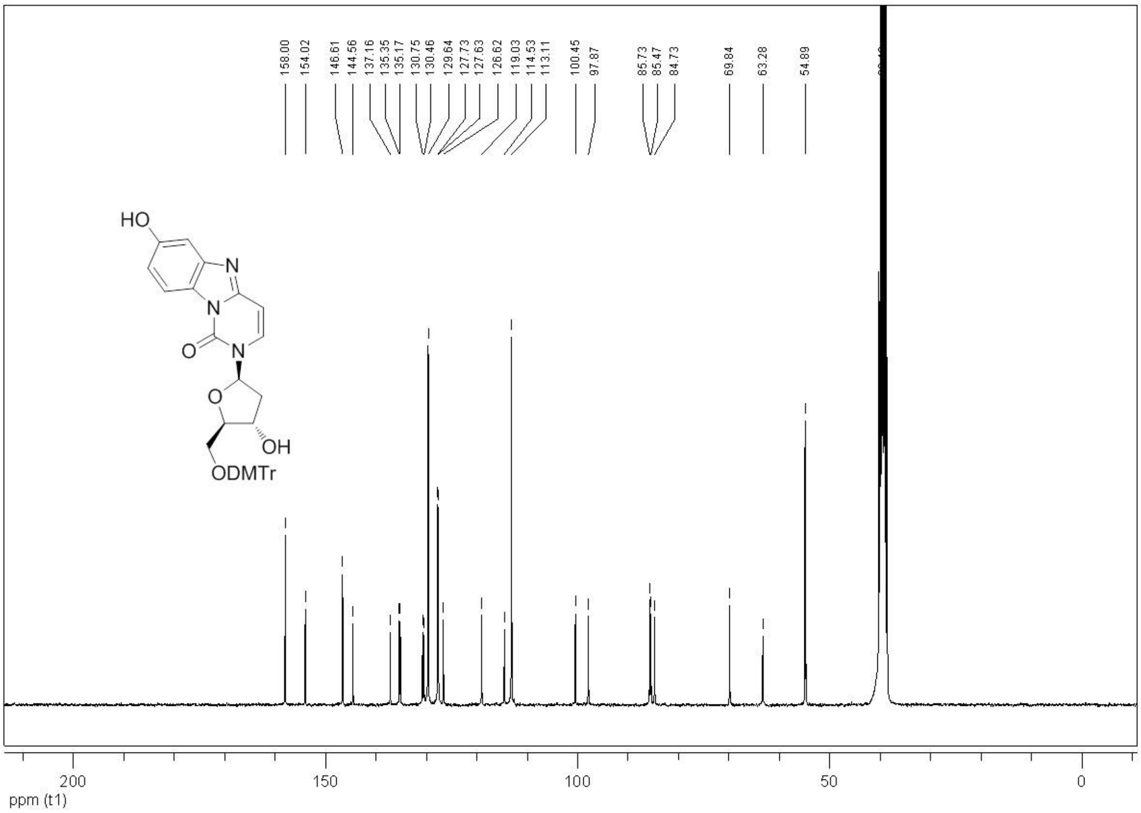

2-((2R,3R,4S,5R)-5-((bis(4-methoxyphenyl)(phenyl)methoxy)methyl)-3,4-dihydroxytetrahydrofuran-2-yl)-7-hydroxybenzo[4,5]imidazo[1,2-c]pyrimidin-1(2H)-one **3b**

^{1}H NMR spectrum

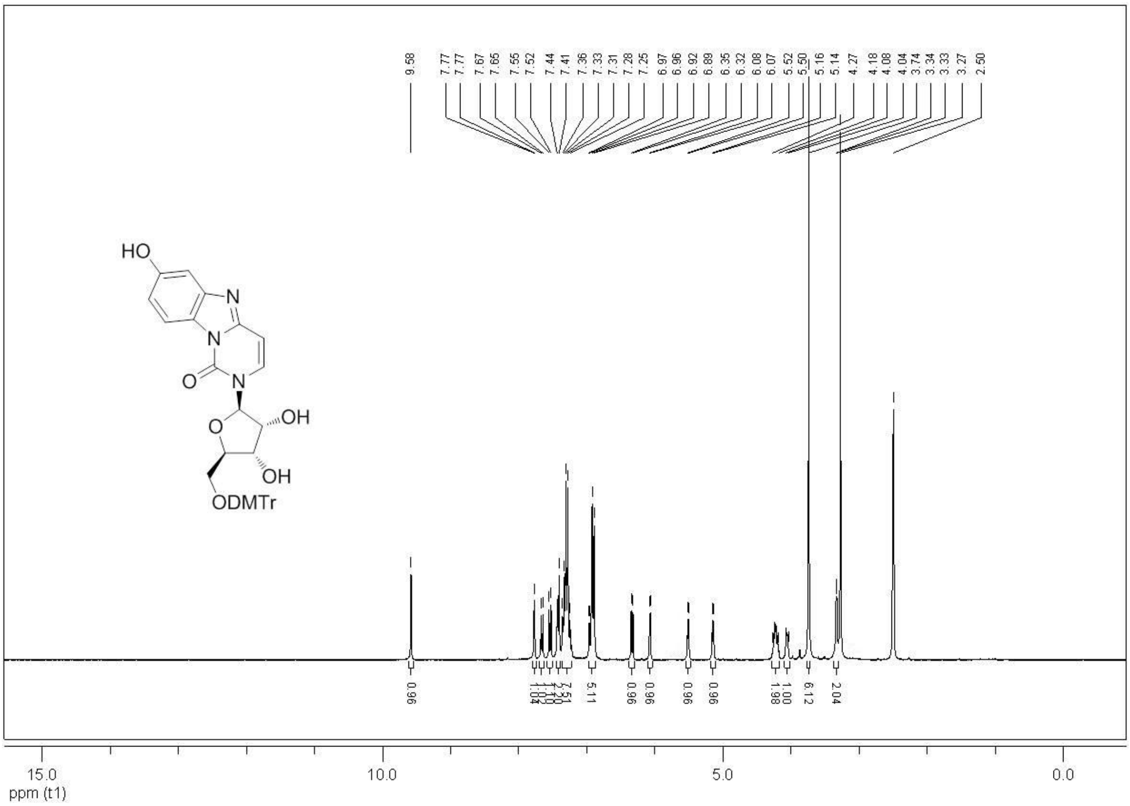

^{13}C NMR spectrum

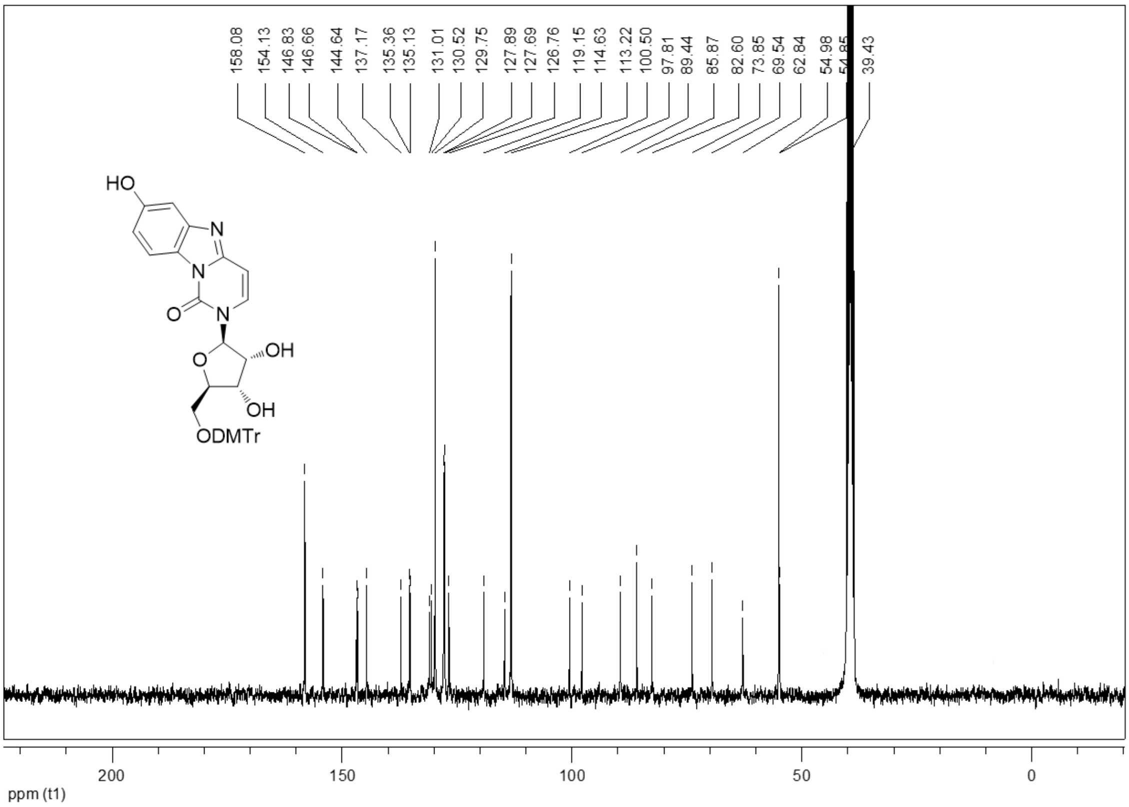

2-((2R,4S,5R)-5-((bis(4-methoxyphenyl)(phenyl)methoxy)methyl)-4-hydroxytetrahydrofuran-2-yl)-7-butoxybenzo[4,5]imidazo[1,2-c]pyrimidin-1(2H)-one **4a**

^{1}H NMR spectrum

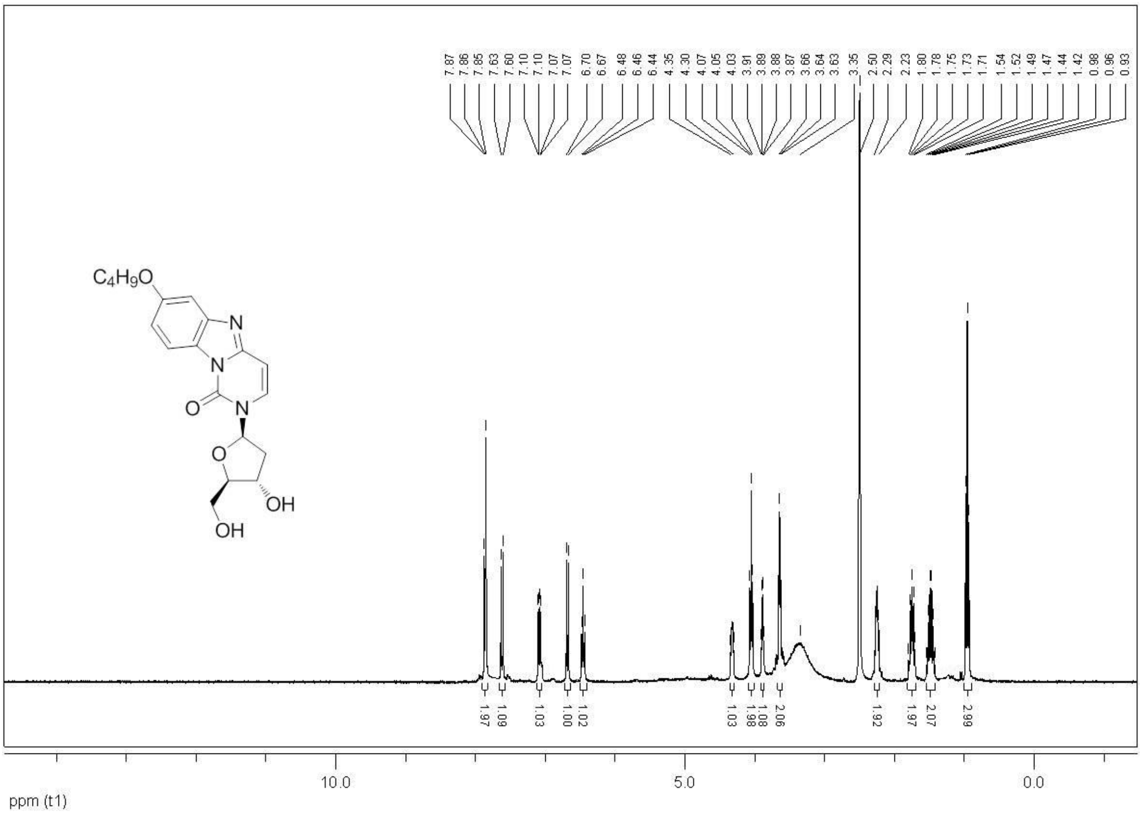

^{13}C NMR spectrum

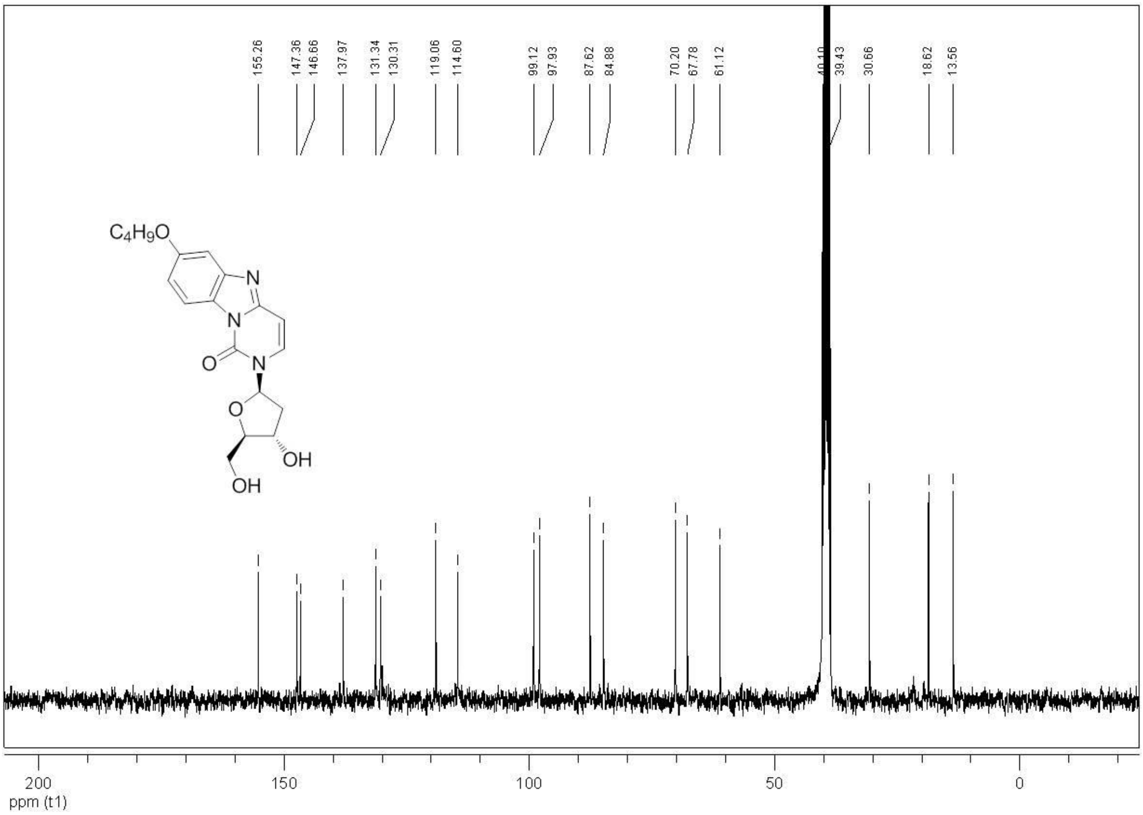

2-((2R,4S,5R)-5-((bis(4-methoxyphenyl)(phenyl)methoxy)methyl)-4-hydroxytetrahydrofuran-2-yl)-7-(hexyloxy)benzo[4,5]imidazo[1,2-c]pyrimidin-1(2H)-one **4b**

^{1}H NMR spectrum

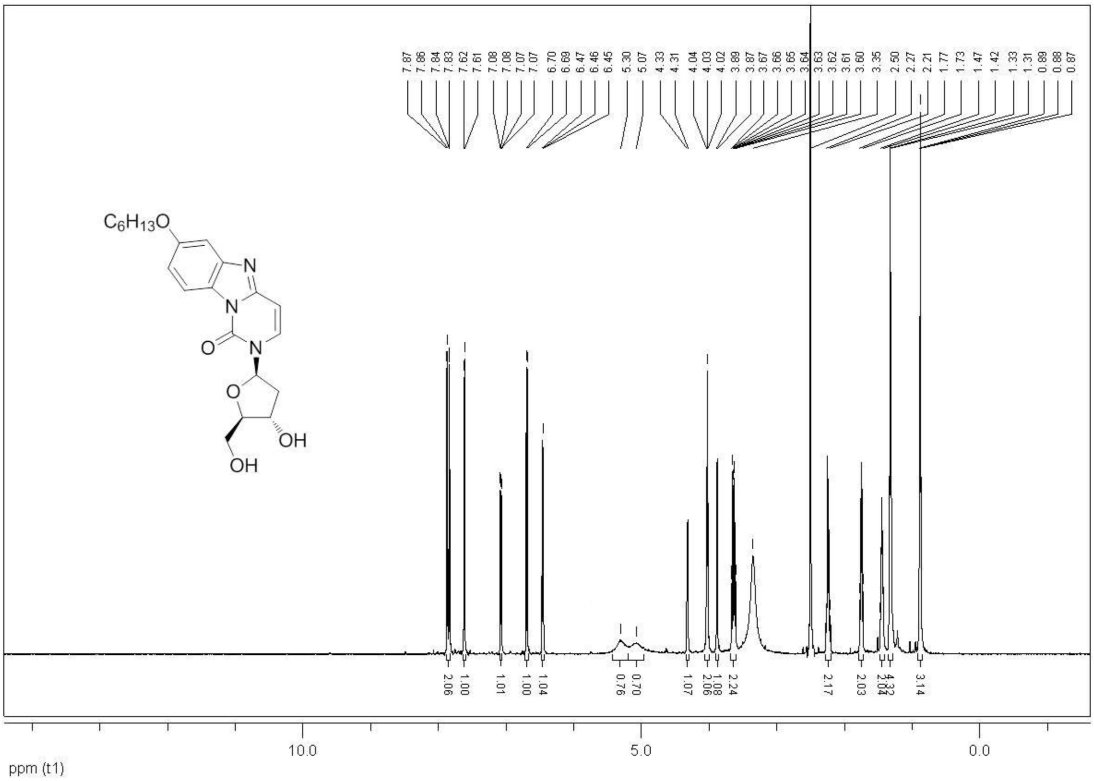

^{13}C NMR spectrum

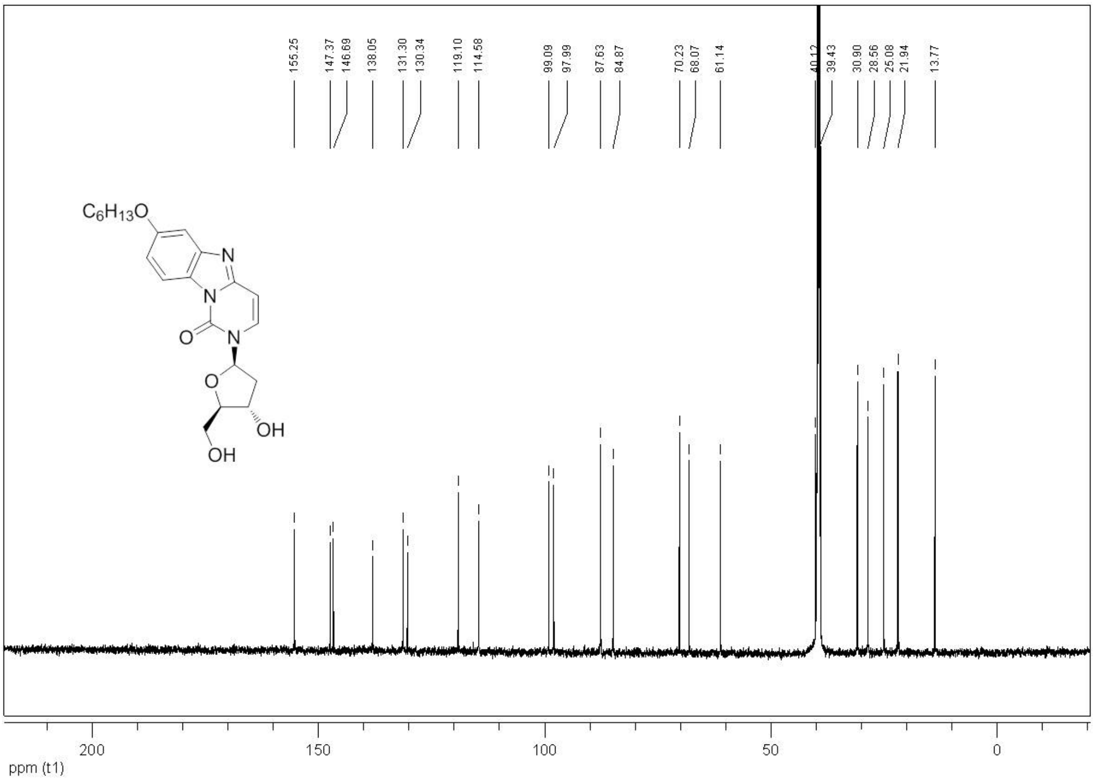

2-((2R,4S,5R)-5-((bis(4-methoxyphenyl)(phenyl)methoxy)methyl)-4-hydroxytetrahydrofuran-2-yl)-7-(octyloxy)benzo[4,5]imidazo[1,2-c]pyrimidin-1(2H)-one **4c**

^{1}H NMR spectrum

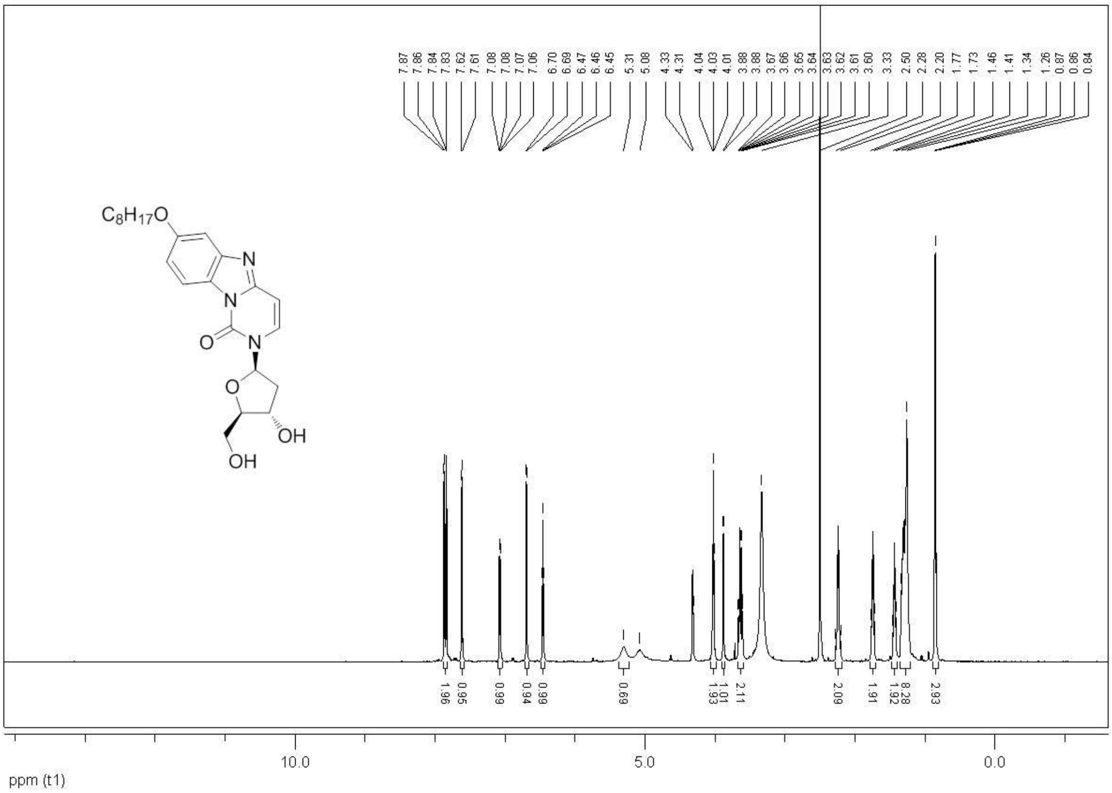

^{13}C NMR spectrum

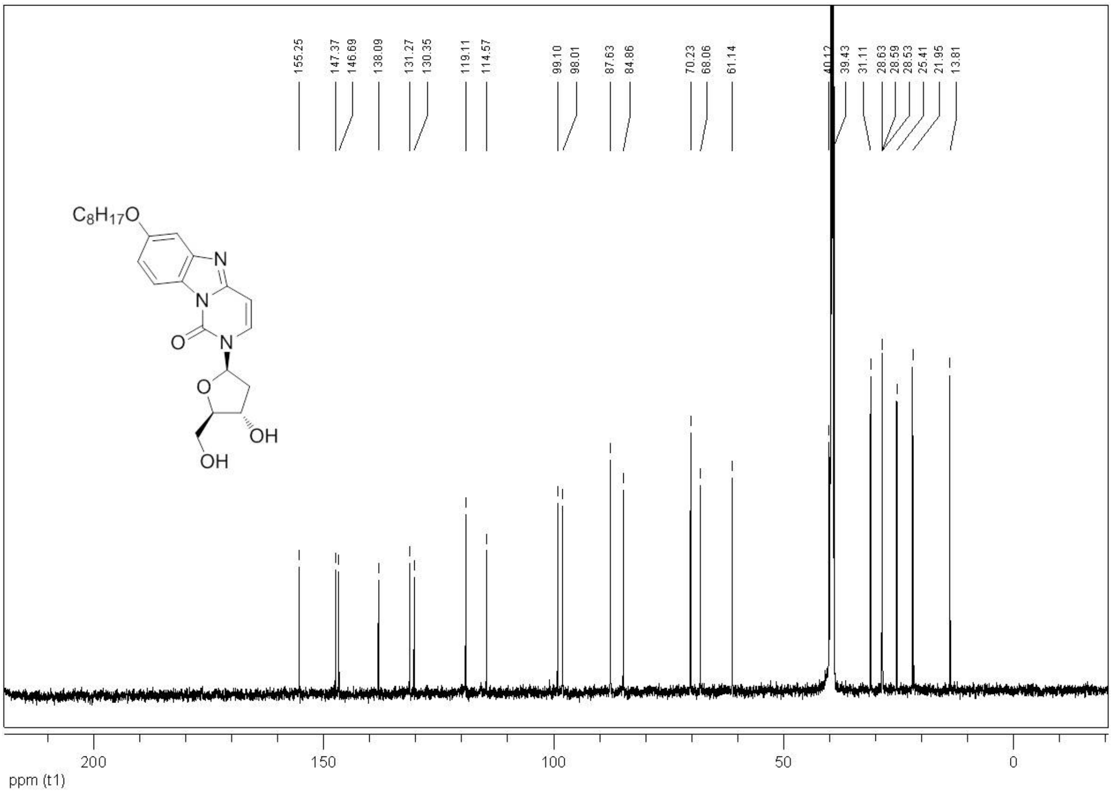

2-((2R,4S,5R)-5-((bis(4-methoxyphenyl)(phenyl)methoxy)methyl)-4-hydroxytetrahydrofuran-2-yl)-7-(decyloxy)benzo[4,5]imidazo[1,2-c]pyrimidin-1(2H)-one **4d**

^{1}H NMR spectrum

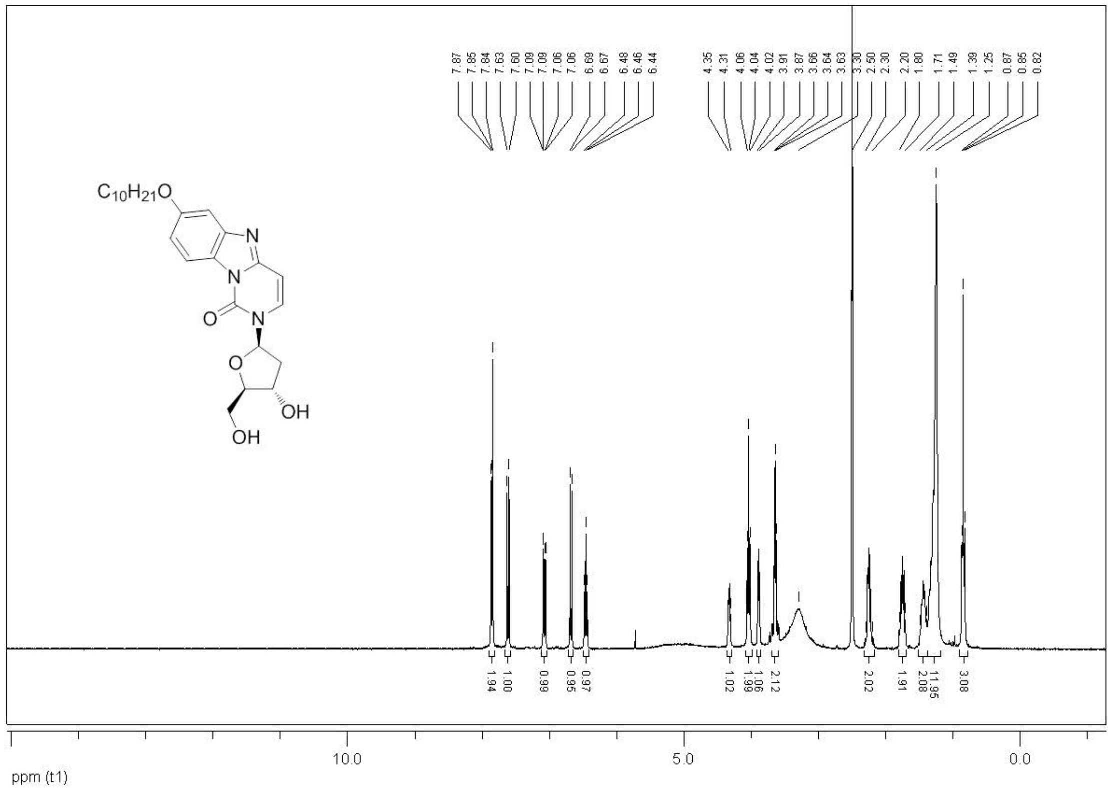

^{13}C NMR spectrum

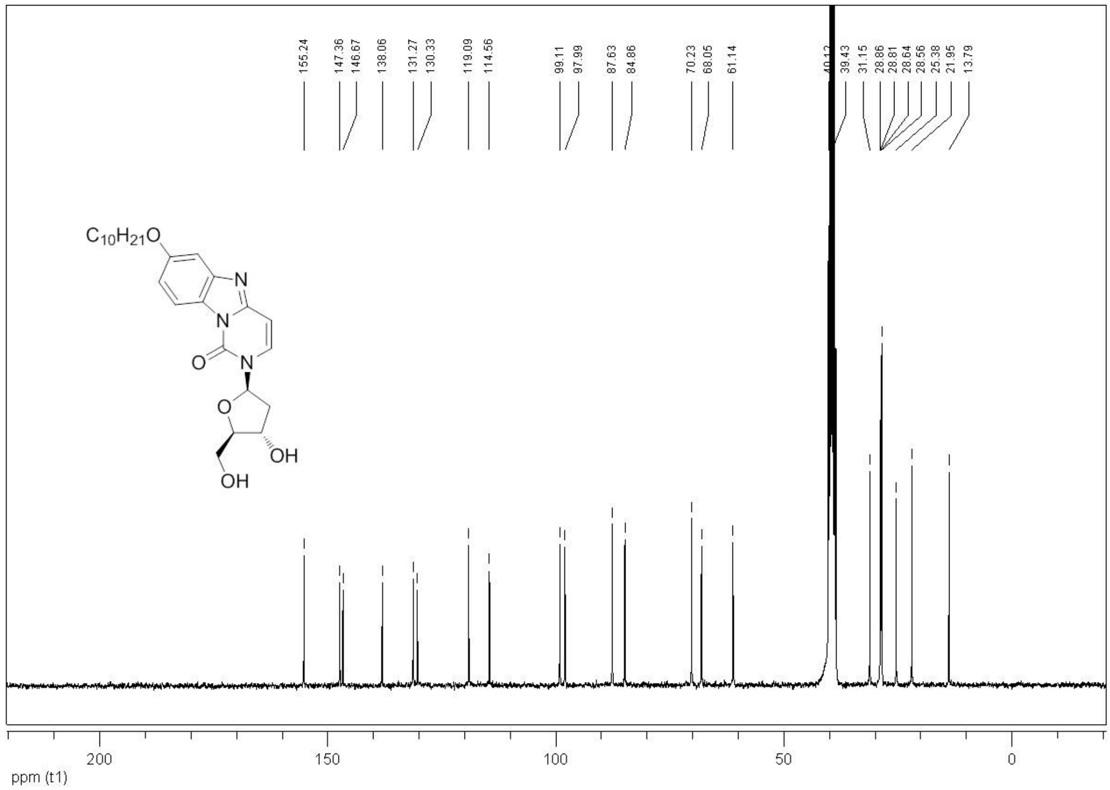

2-((2R,4S,5R)-5-((bis(4-methoxyphenyl)(phenyl)methoxy)methyl)-4-hydroxytetrahydrofuran-2-yl)-7-(dodecyloxy)benzo[4,5]imidazo[1,2-c]pyrimidin-1(2H)-one **4e**

^{1}H NMR spectrum

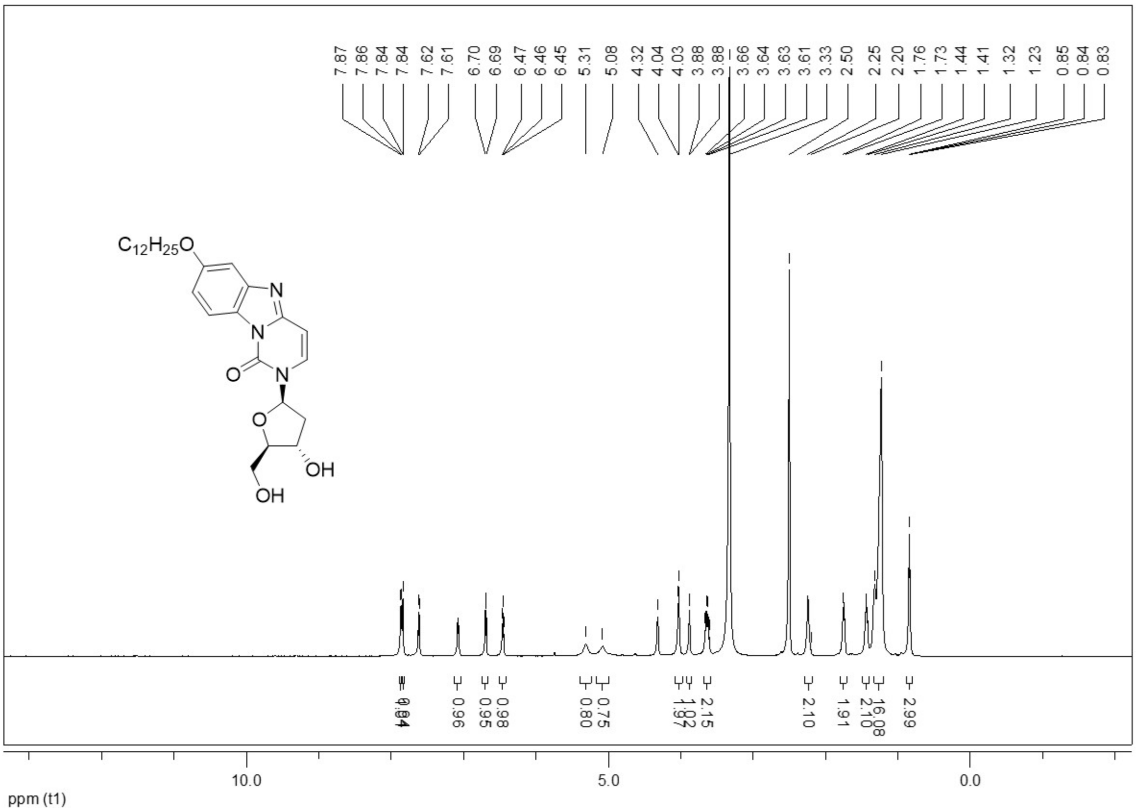

^{13}C NMR spectrum

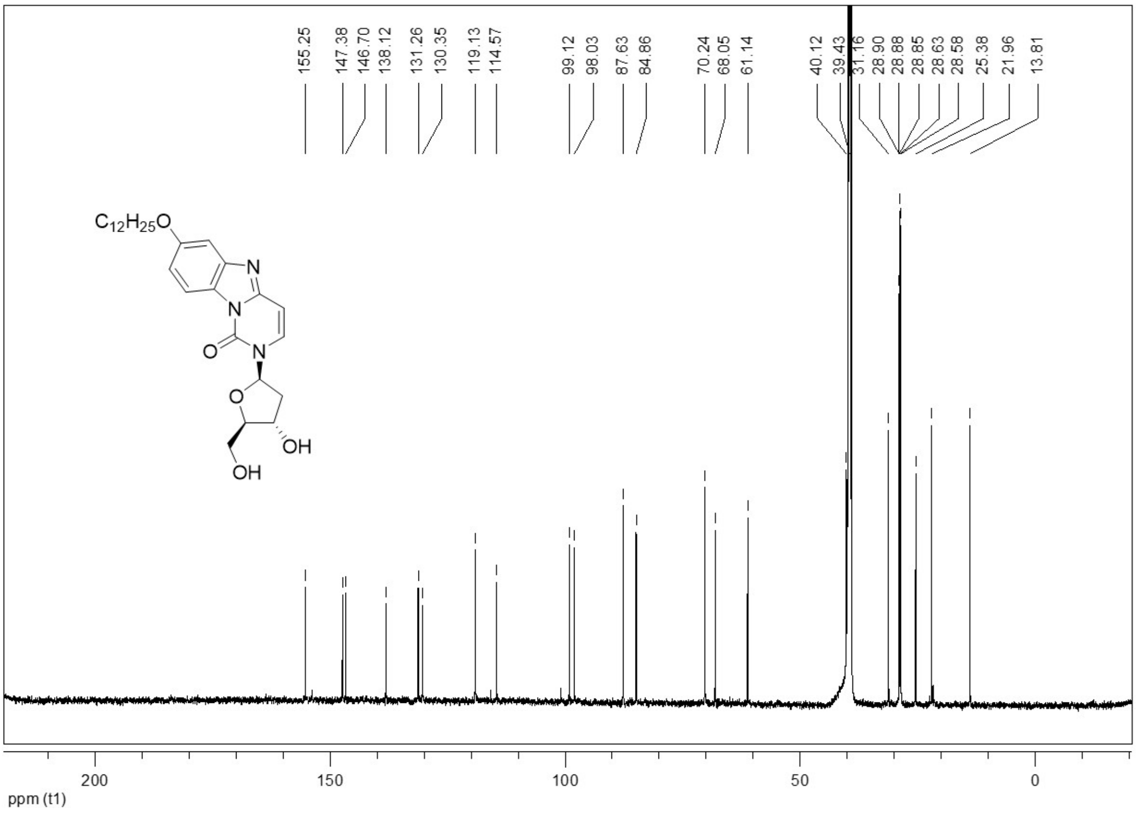

2-((2R,3R,4S,5R)-5-((bis(4-methoxyphenyl)(phenyl)methoxy)methyl)-3,4-dihydroxytetrahydrofuran-2-yl)-7-butoxybenzo[4,5]imidazo[1,2-c]pyrimidin-1(2H)-one **5a**

^{1}H NMR spectrum

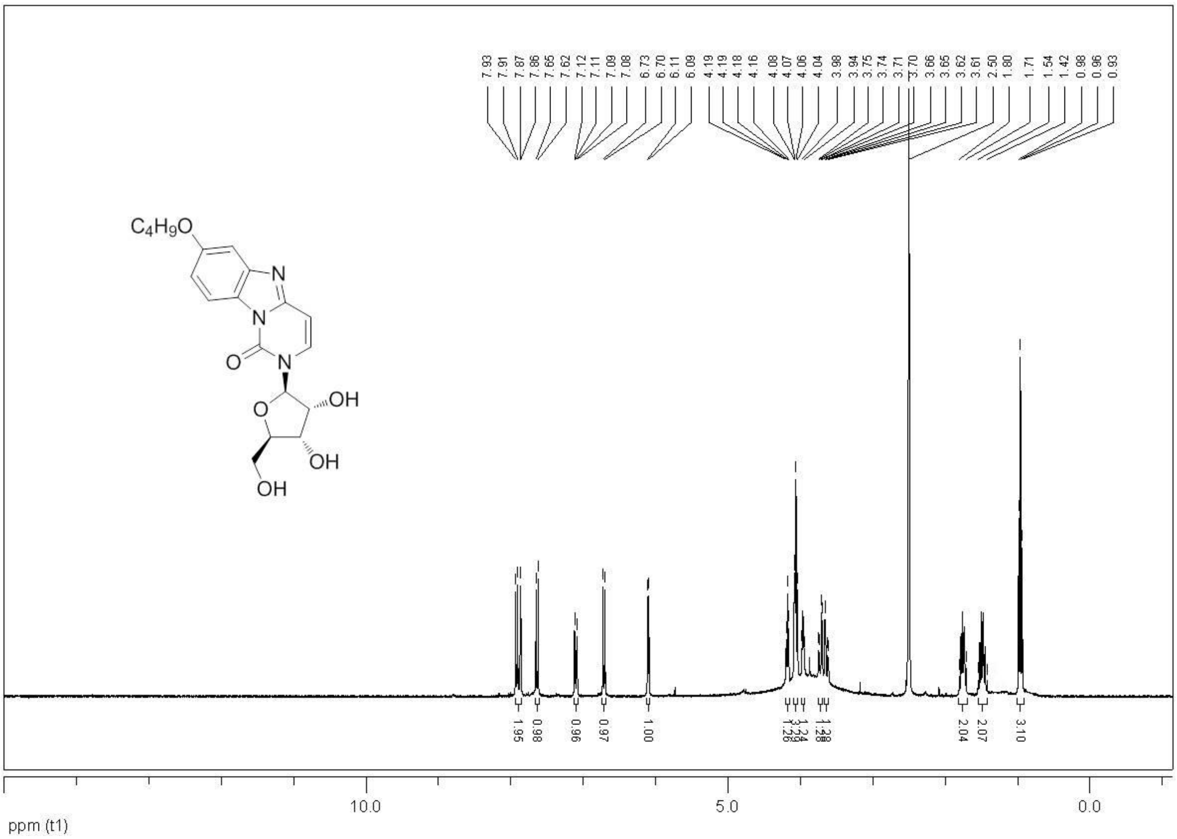

^{13}C NMR spectrum

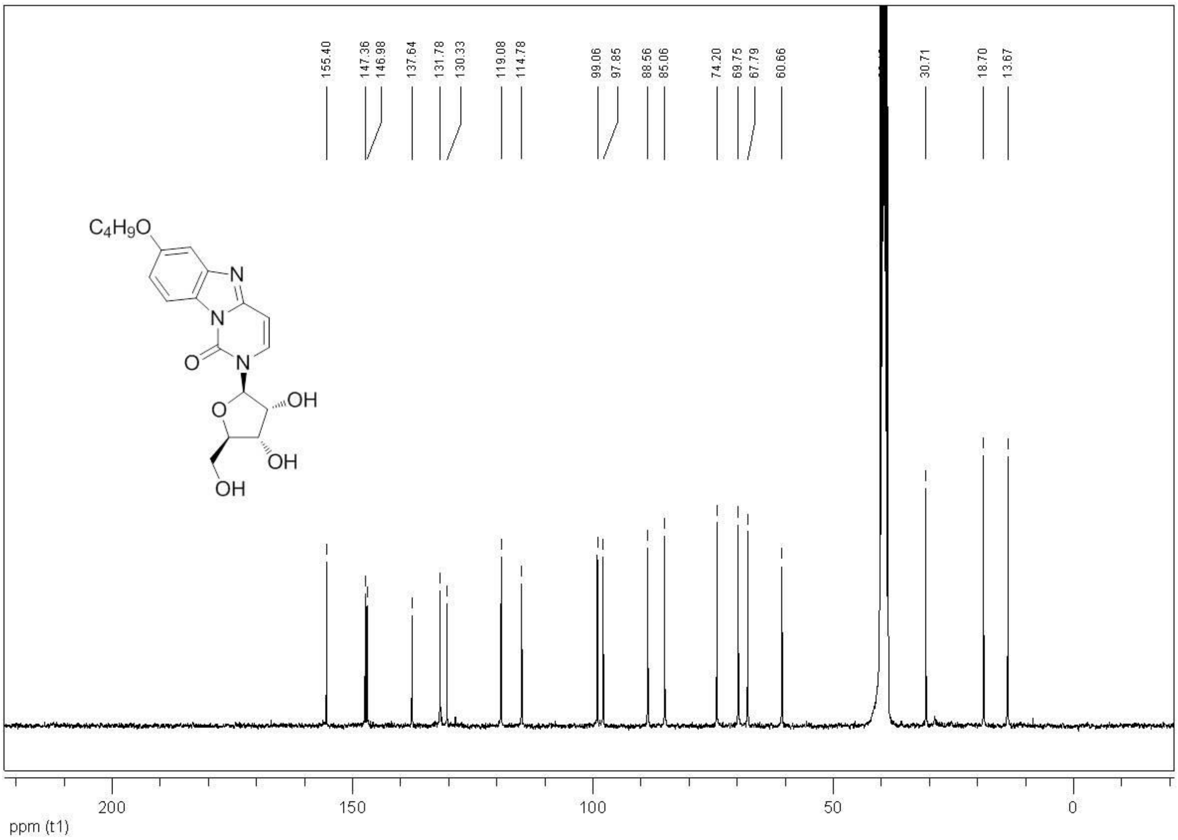

2-((2R,3R,4S,5R)-5-((bis(4-methoxyphenyl)(phenyl)methoxy)methyl)-3,4-dihydroxytetrahydrofuran-2-yl)-7-(hexyloxy)benzo[4,5]imidazo[1,2-c]pyrimidin-1(2H)-one **5b**

^{1}H NMR spectrum

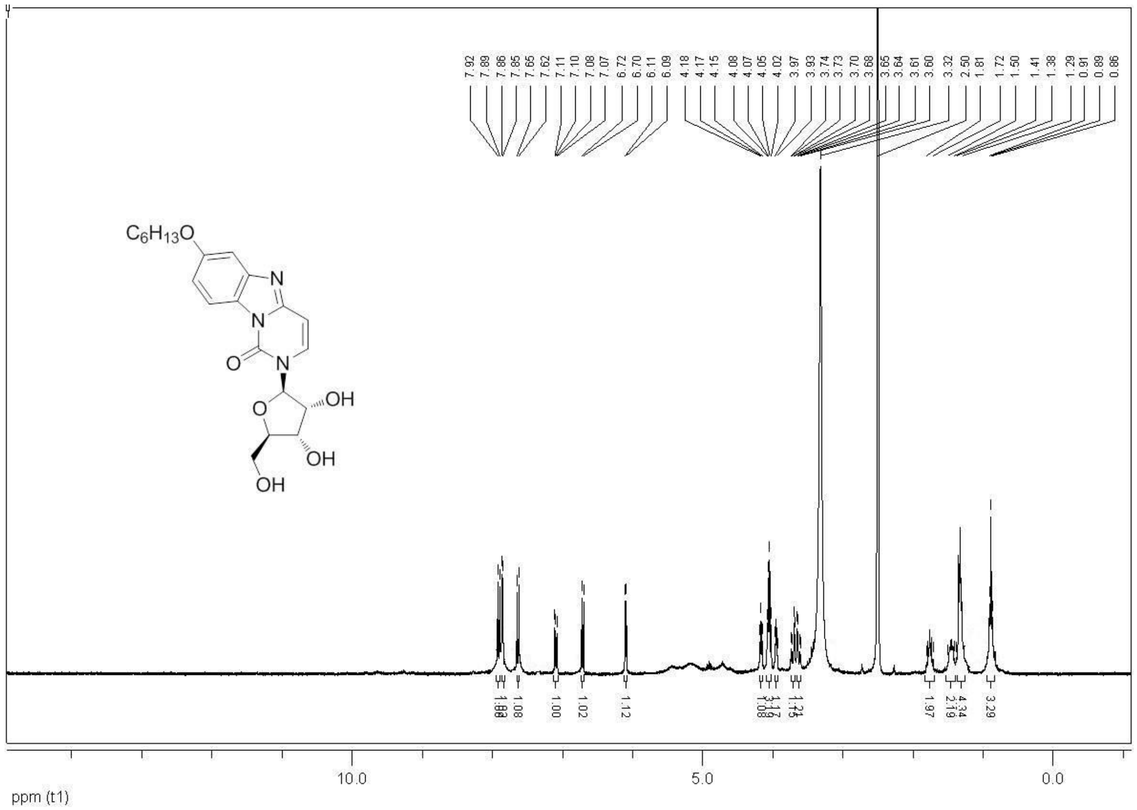

^{13}C NMR spectrum

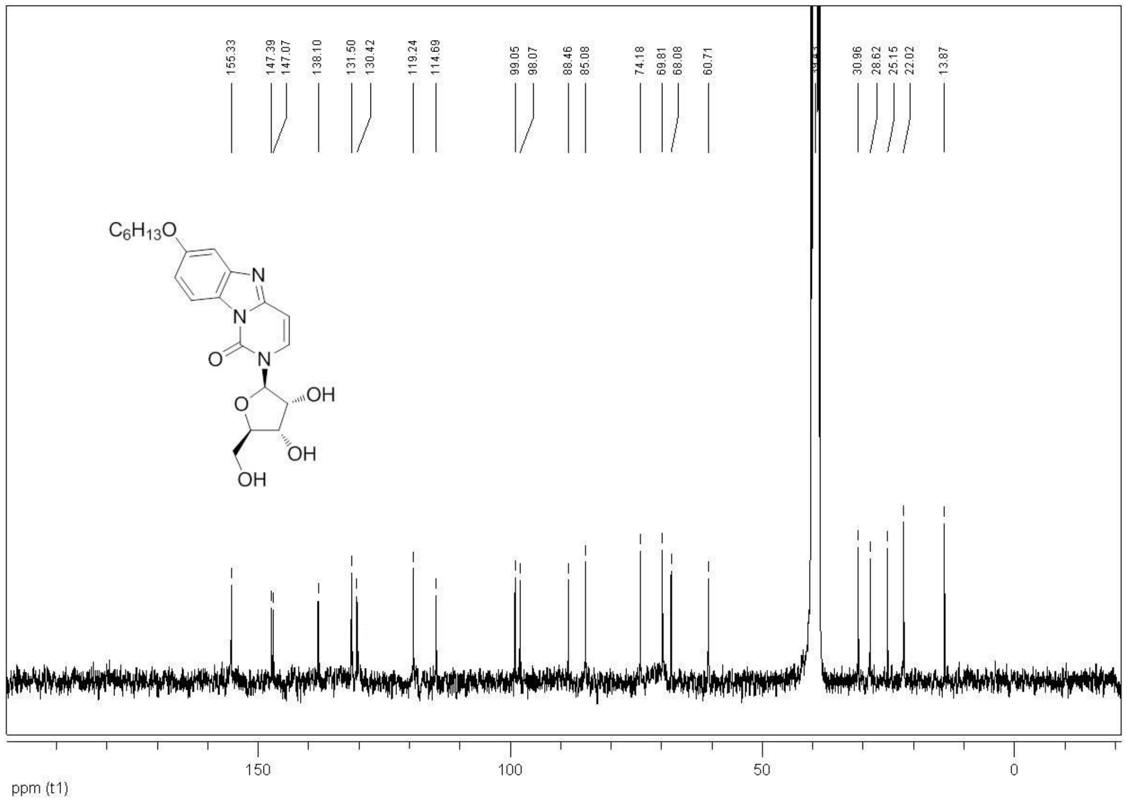

2-((2R,3R,4S,5R)-5-((bis(4-methoxyphenyl)(phenyl)methoxy)methyl)-3,4-dihydroxytetrahydrofuran-2-yl)-7-(octyloxy)benzo[4,5]imidazo[1,2-c]pyrimidin-1(2H)-one **5c**

^{1}H NMR spectrum

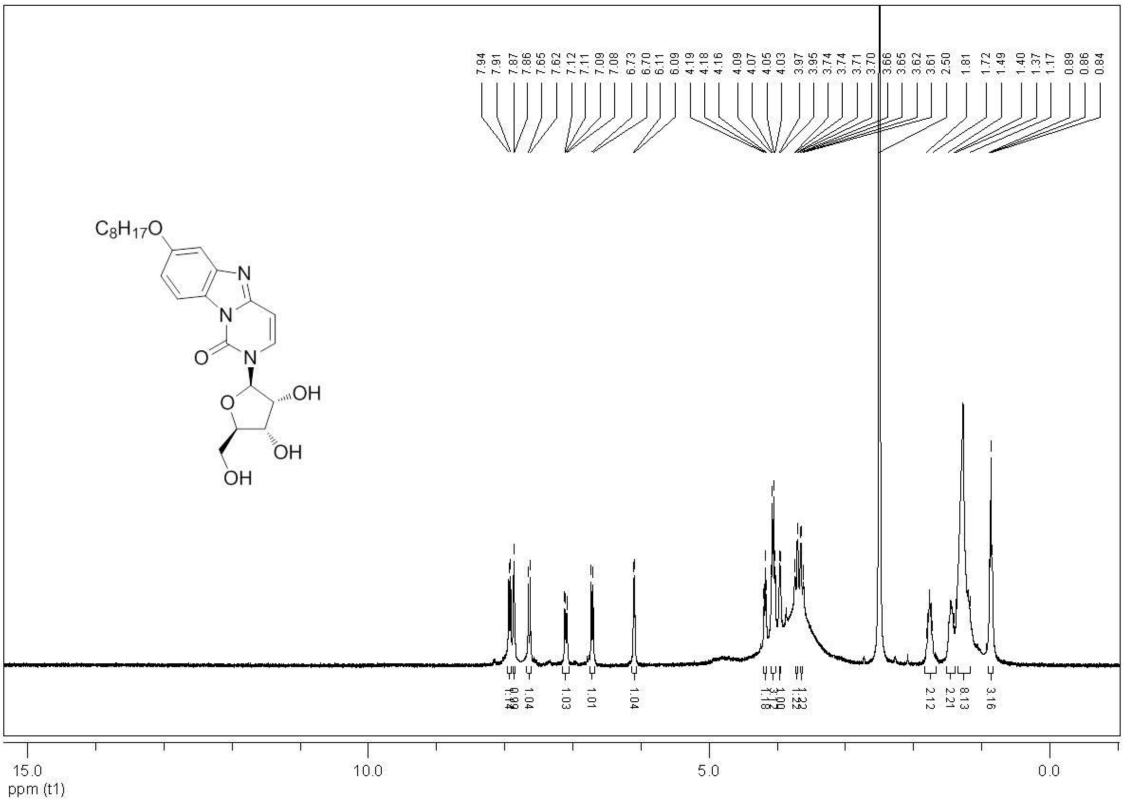

^{13}C NMR spectrum

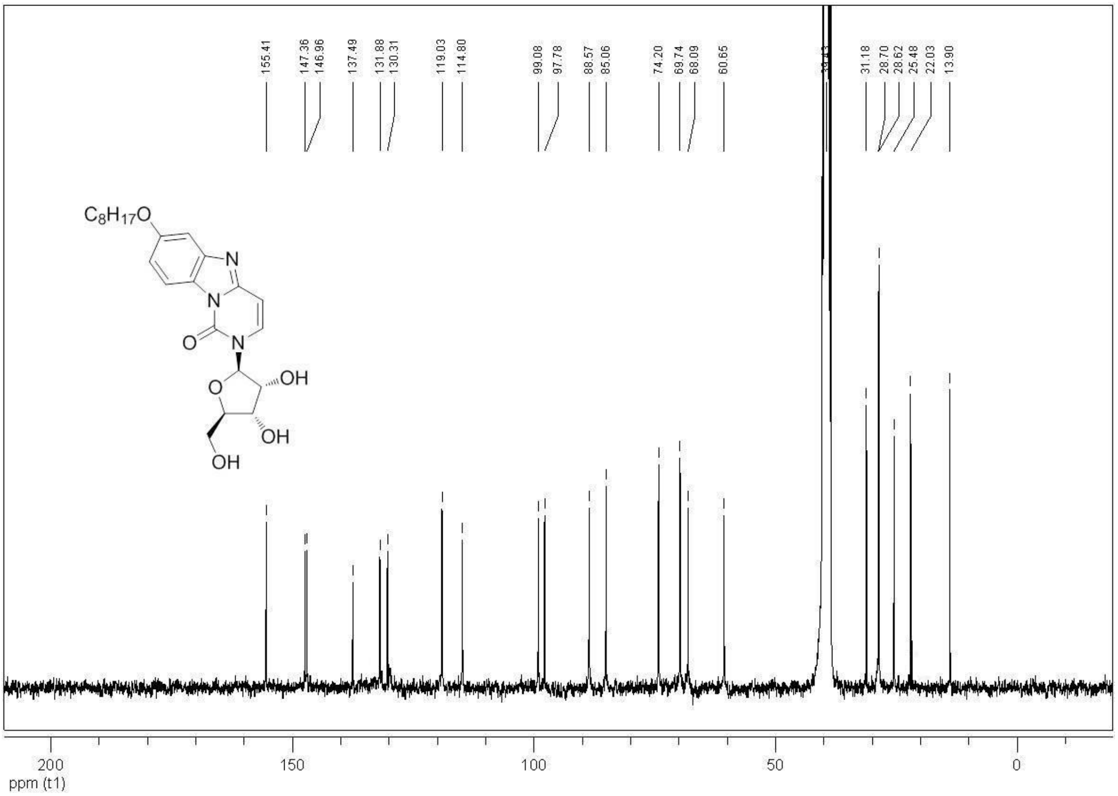

2-((2R,3R,4S,5R)-5-((bis(4-methoxyphenyl)(phenyl)methoxy)methyl)-3,4-dihydroxytetrahydrofuran-2-yl)-7-(decyloxy)benzo[4,5]imidazo[1,2-c]pyrimidin-1(2H)-one **5d**

^{1}H NMR spectrum

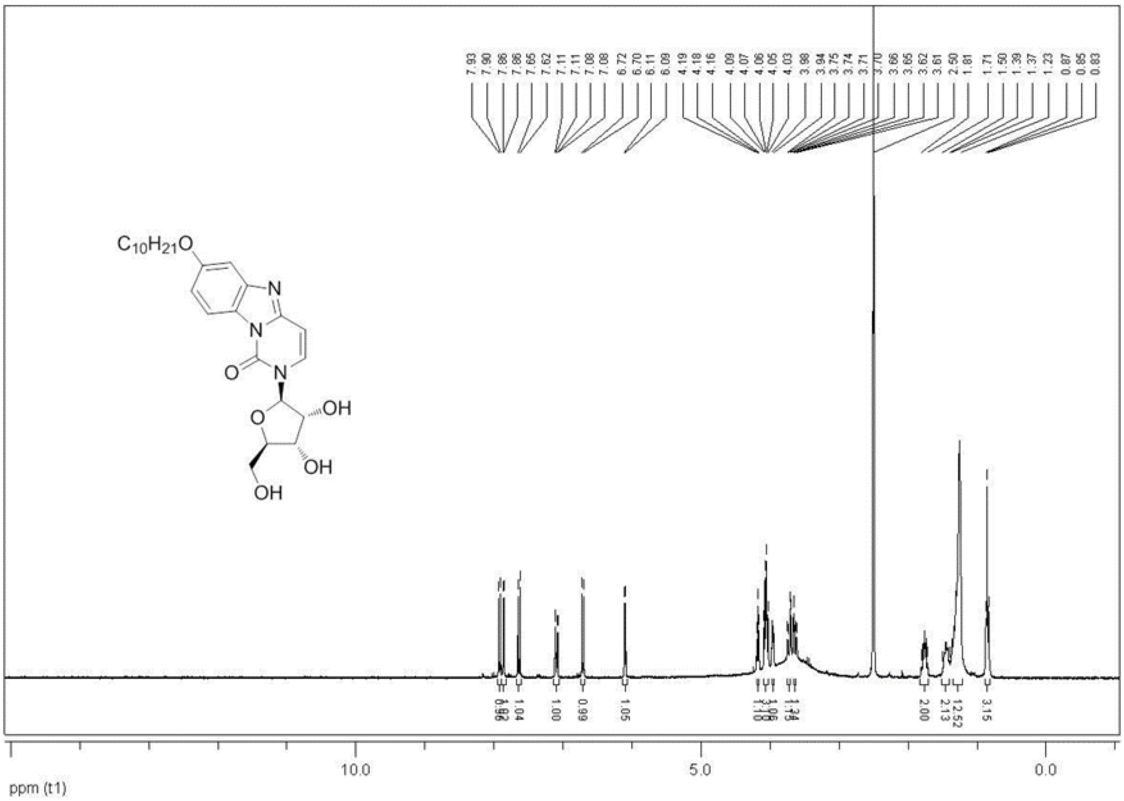

^{13}C NMR spectrum

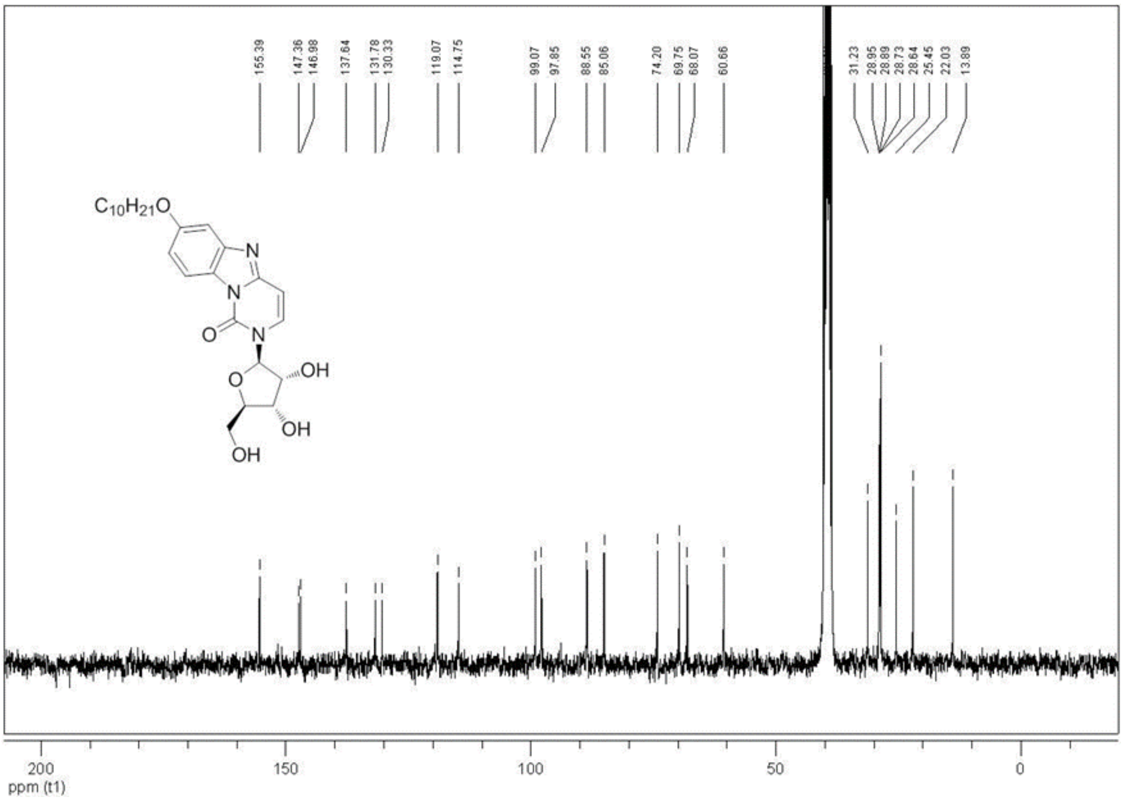

2-((2R,3R,4S,5R)-5-((bis(4-methoxyphenyl)(phenyl)methoxy)methyl)-3,4-dihydroxytetrahydrofuran-2-yl)-7-(dodecyloxy)benzo[4,5]imidazo[1,2-c]pyrimidin-1(2H)-one **5e**

^{1}H NMR spectrum

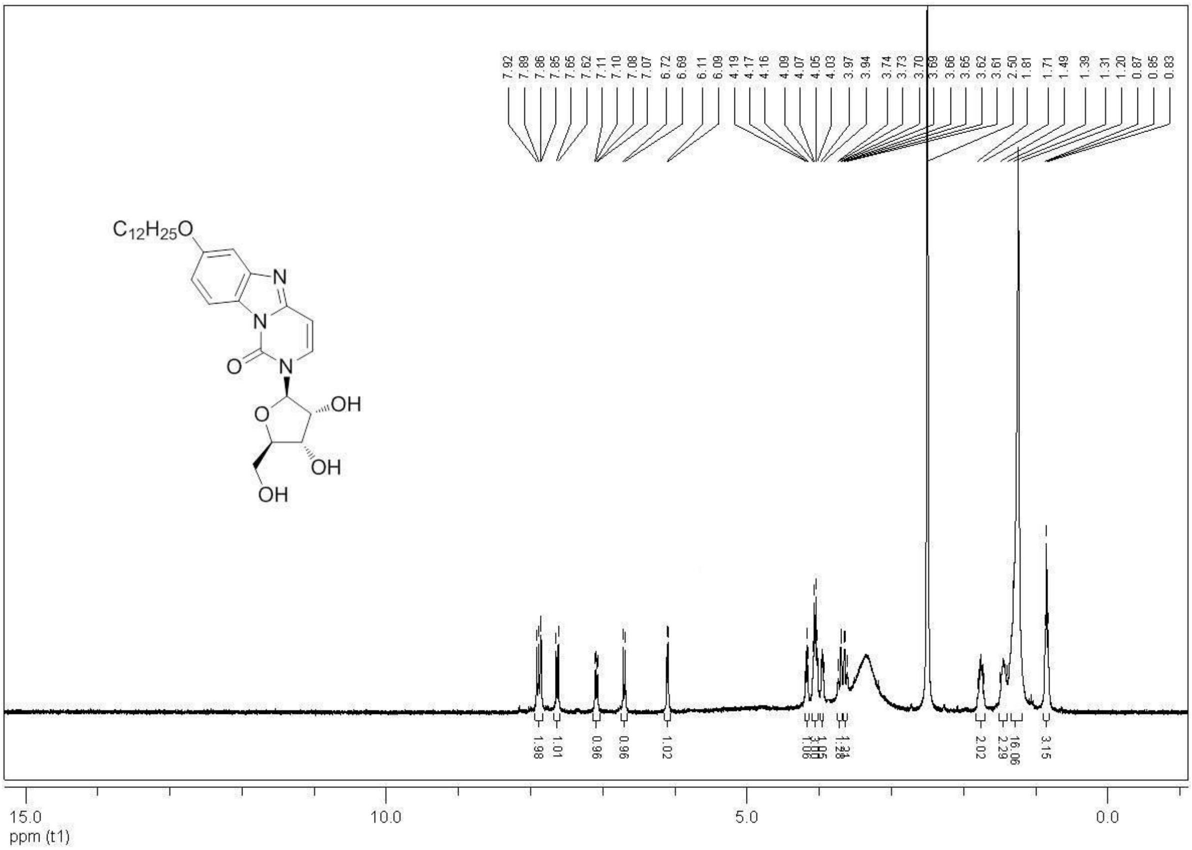

^{13}C NMR spectrum

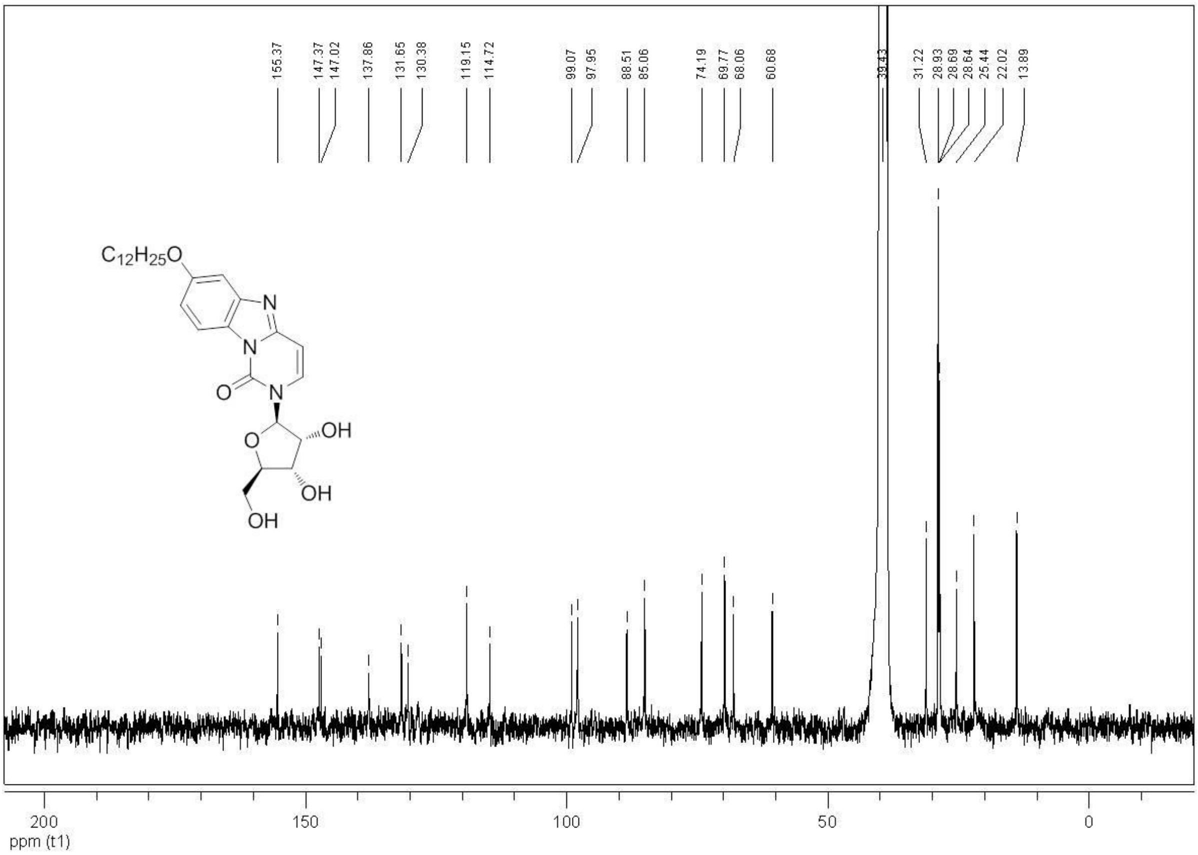

7-butoxybenzo[4,5]imidazo[1,2-c]pyrimidin-1(2H)-one **6a**

^{1}H NMR spectrum

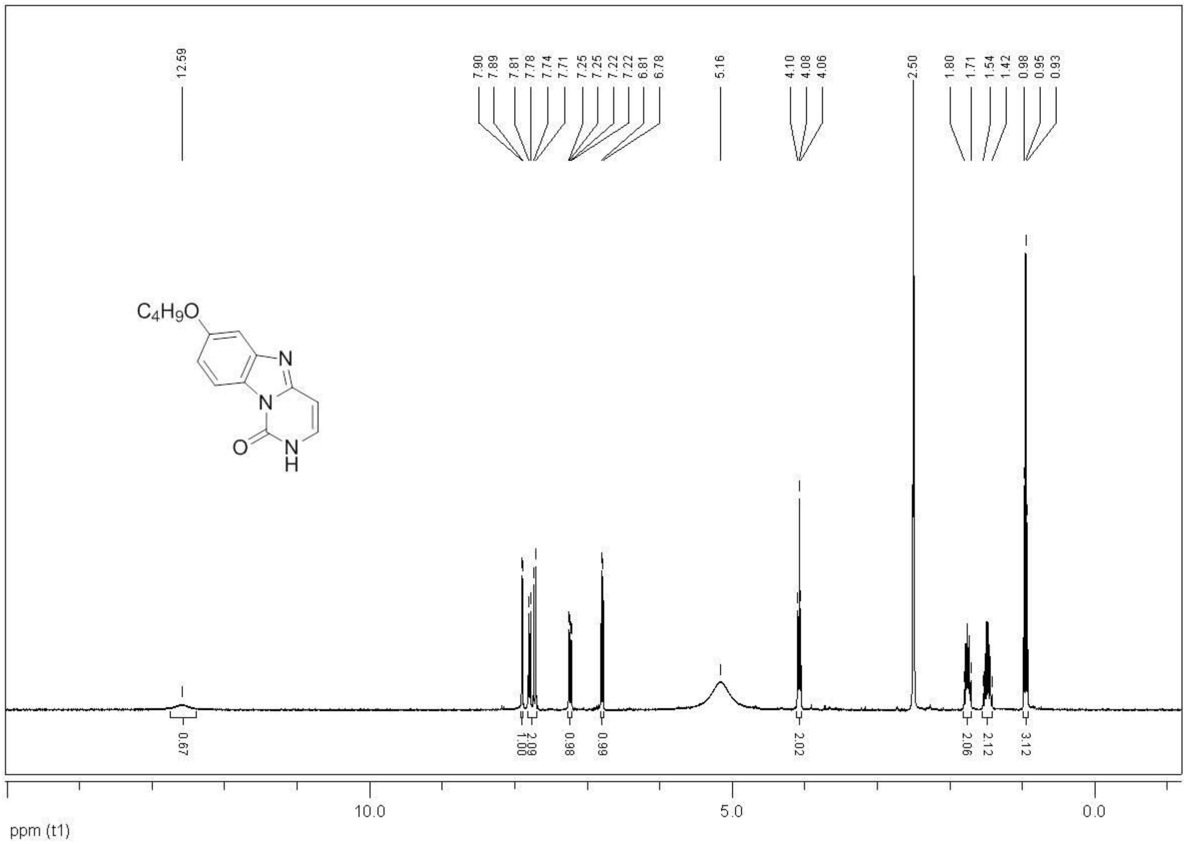

^{13}C NMR spectrum

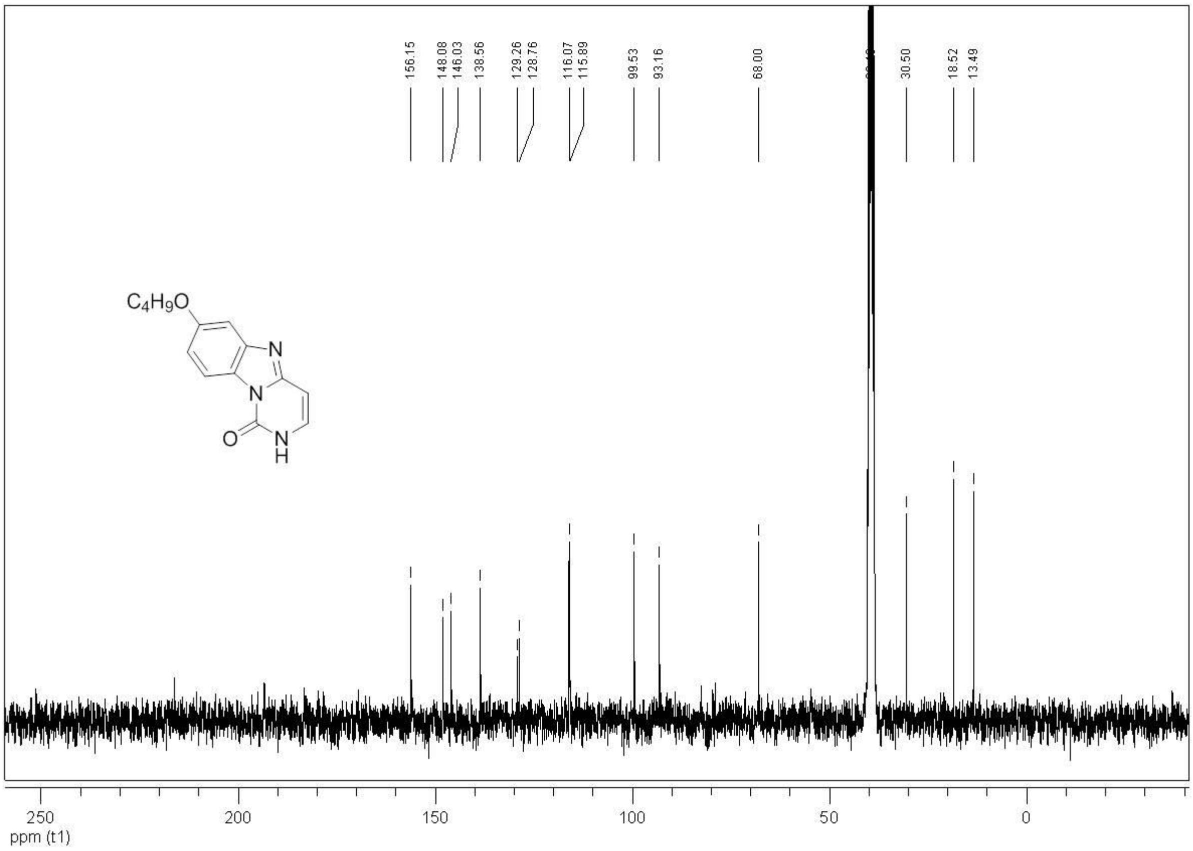

7-(hexyloxy)benzo[4,5]imidazo[1,2-c]pyrimidin-1(2H)-one **6b**

^{1}H NMR spectrum

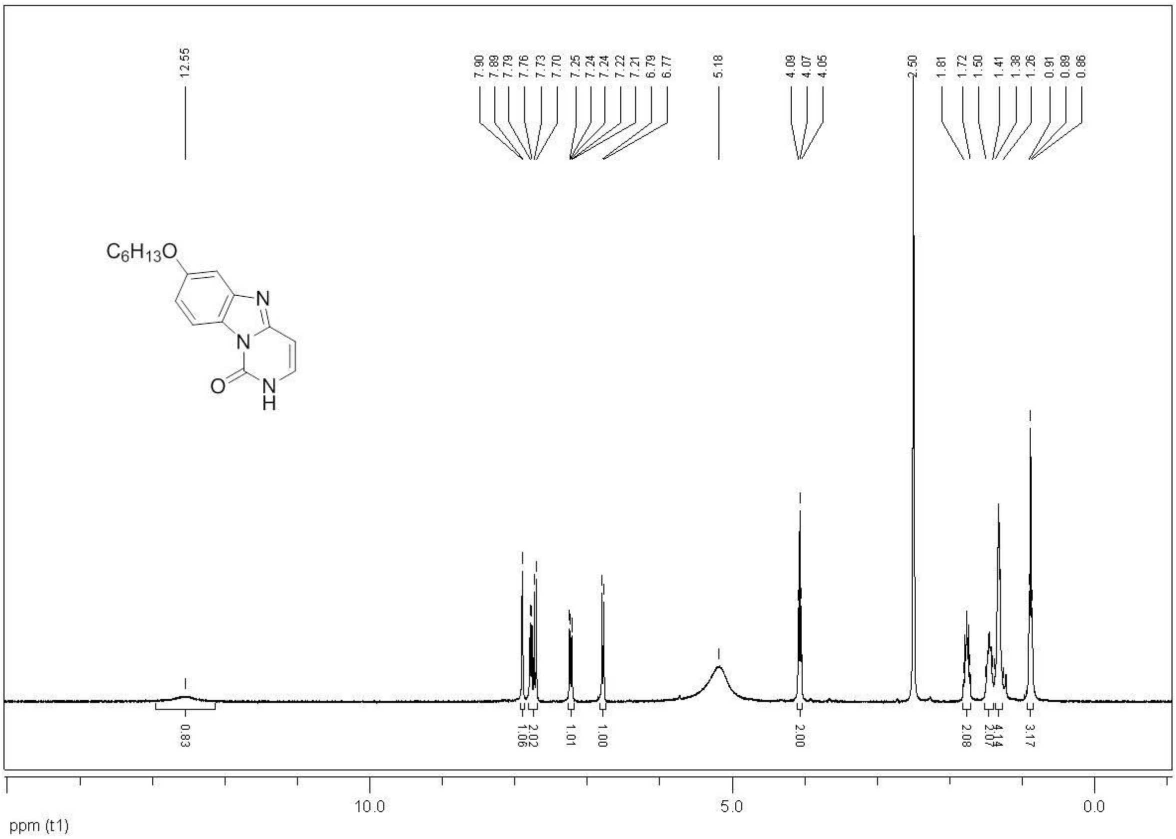

^{13}C NMR spectrum

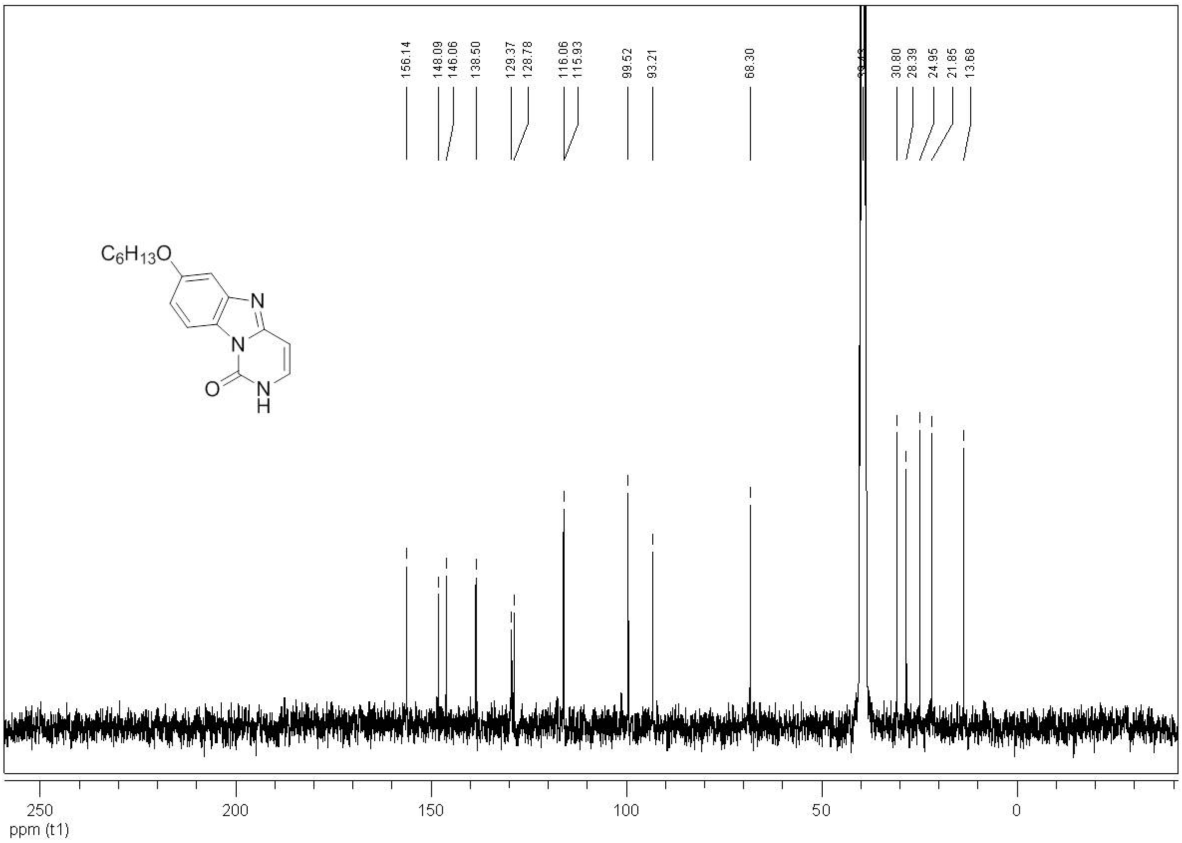

7-(octyloxy)benzo[4,5]imidazo[1,2-c]pyrimidin-1(2H)-one **6c**

^{1}H NMR spectrum

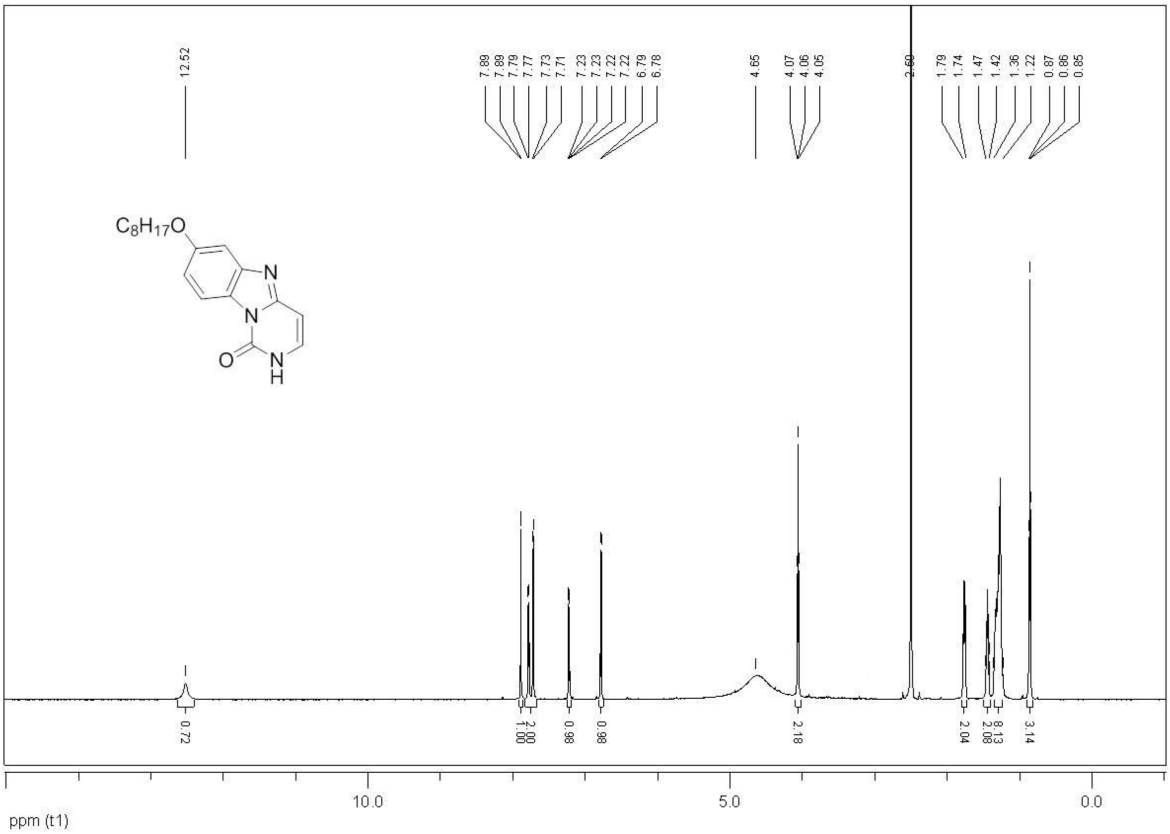

^{13}C NMR spectrum

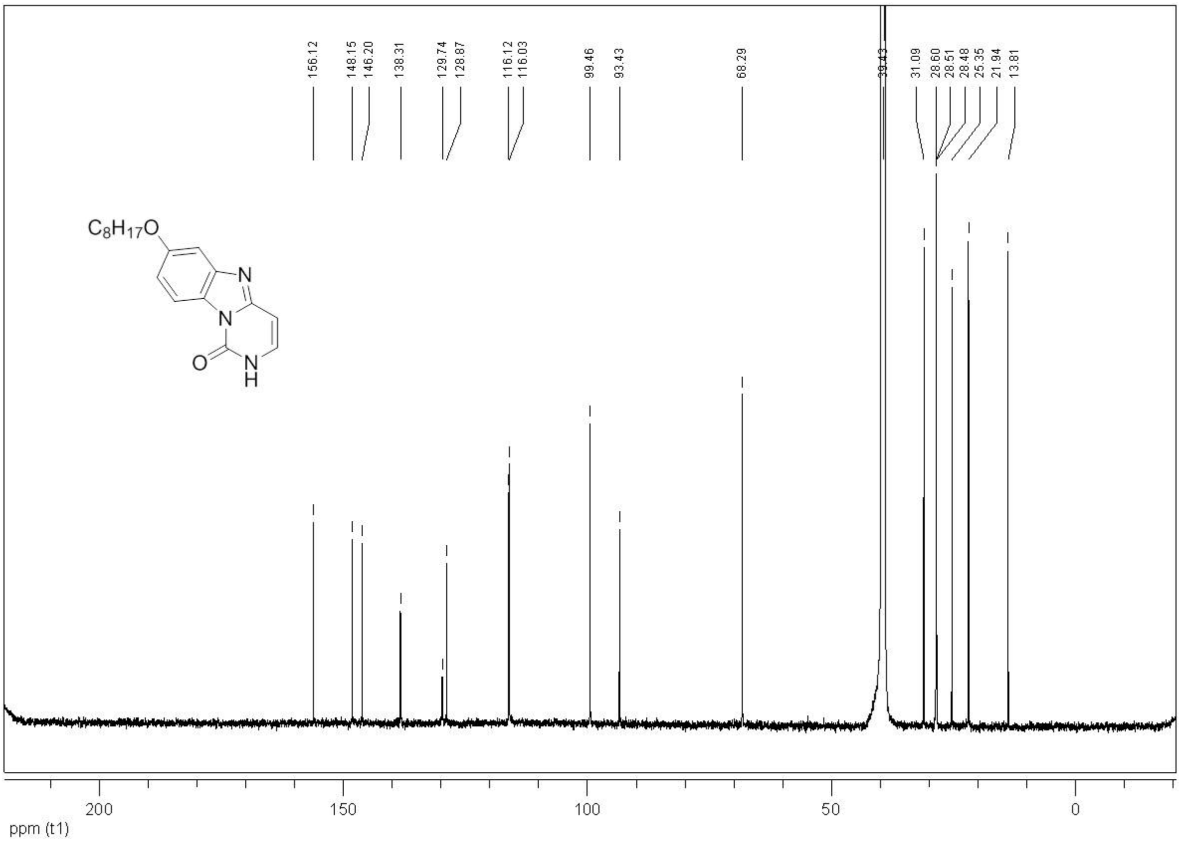

7-(decyloxy)benzo[4,5]imidazo[1,2-c]pyrimidin-1(2H)-one **6d**

^{1}H NMR spectrum

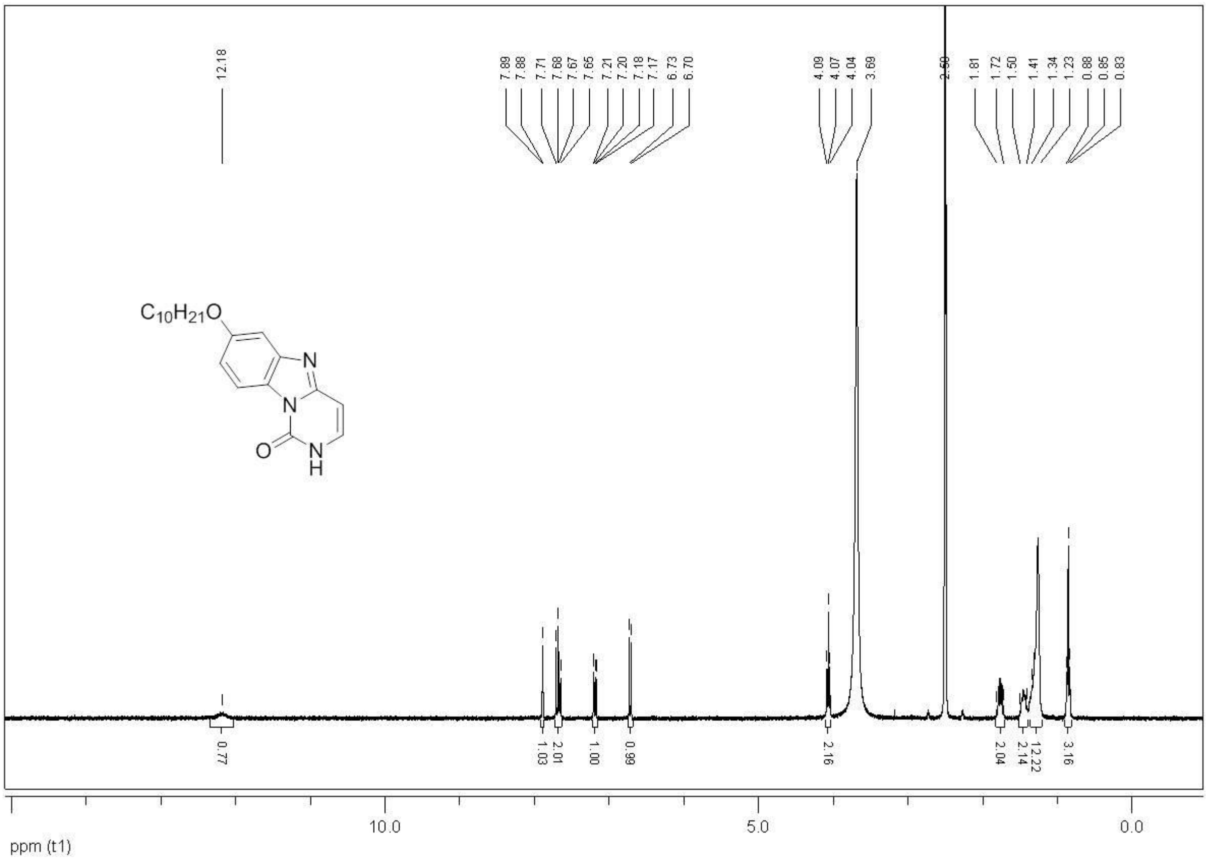

^{13}C NMR spectrum

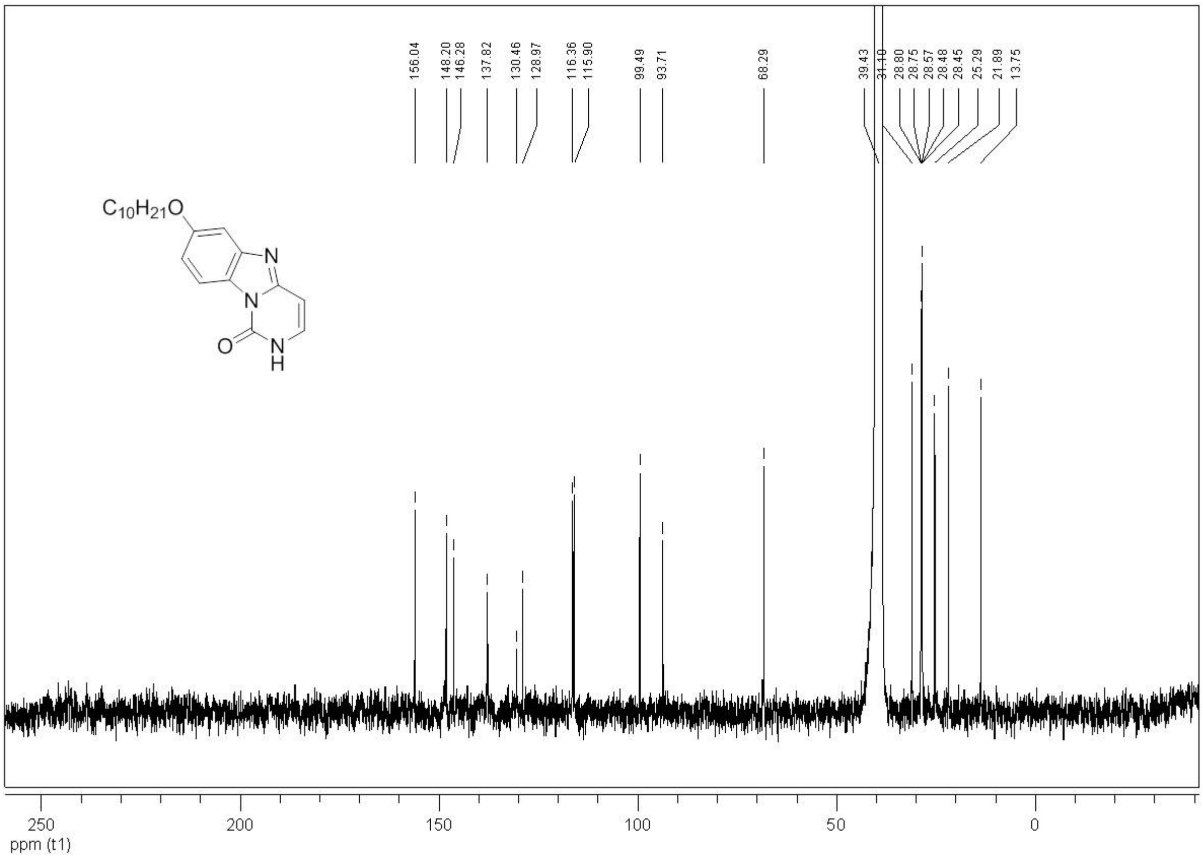

7-(dodecyloxy)benzo[4,5]imidazo[1,2-c]pyrimidin-1(2H)-one **6e**

^{1}H NMR spectrum

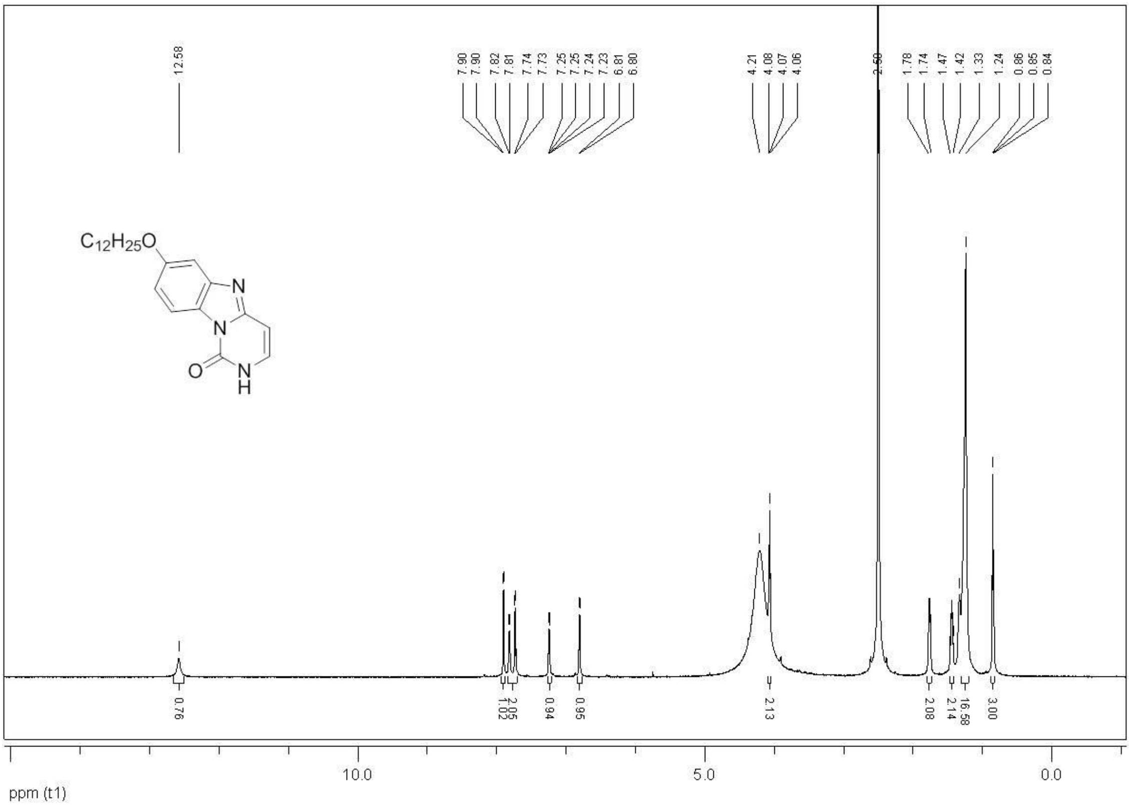

^{13}C NMR spectrum

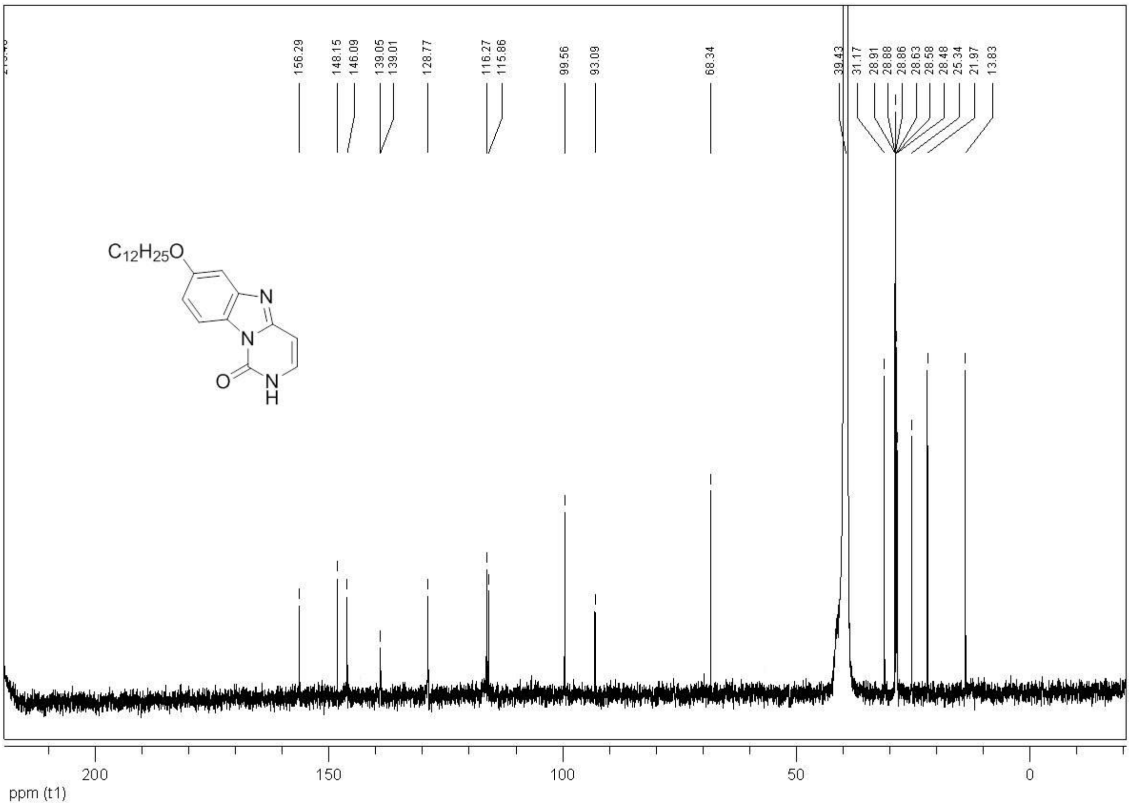

2-((2R,4S,5R)-5-((bis(4-methoxyphenyl)(phenyl)methoxy)methyl)-4-hydroxytetrahydrofuran-2-yl)-7-methoxybenzo[4,5]imidazo[1,2-c]pyrimidin-1(2H)-one **7a**

^{1}H NMR spectrum

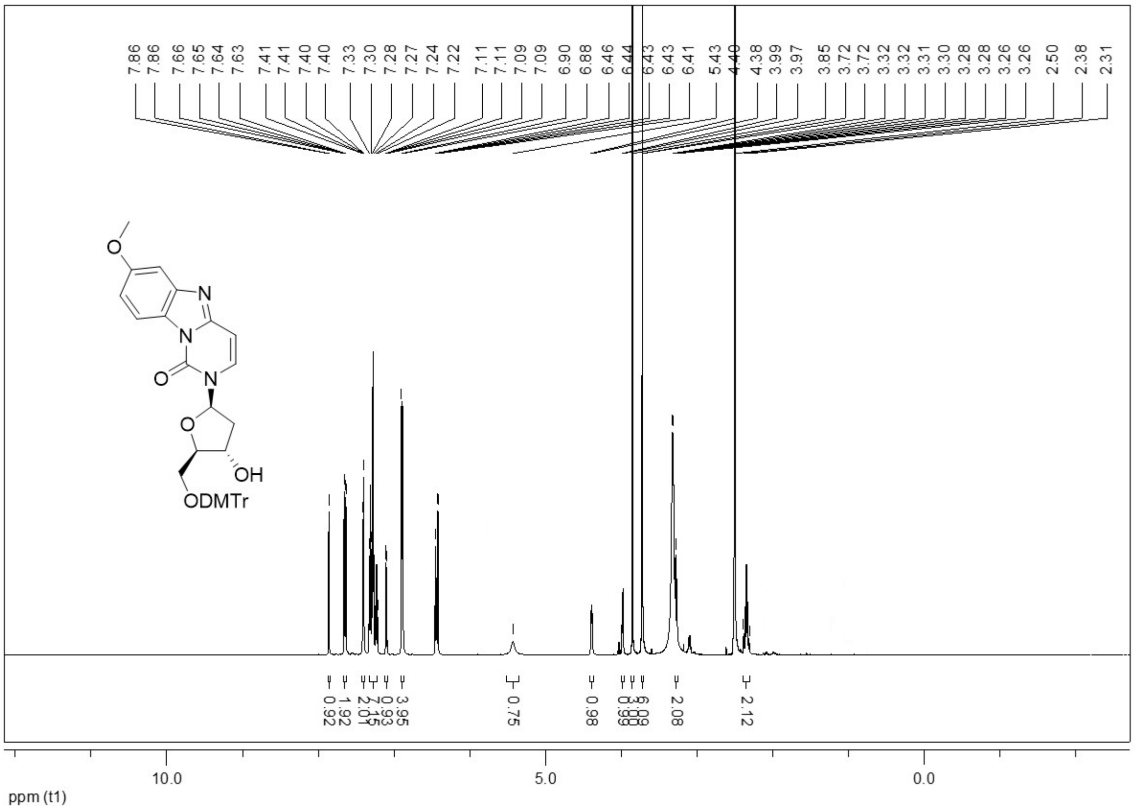

^{13}C NMR spectrum

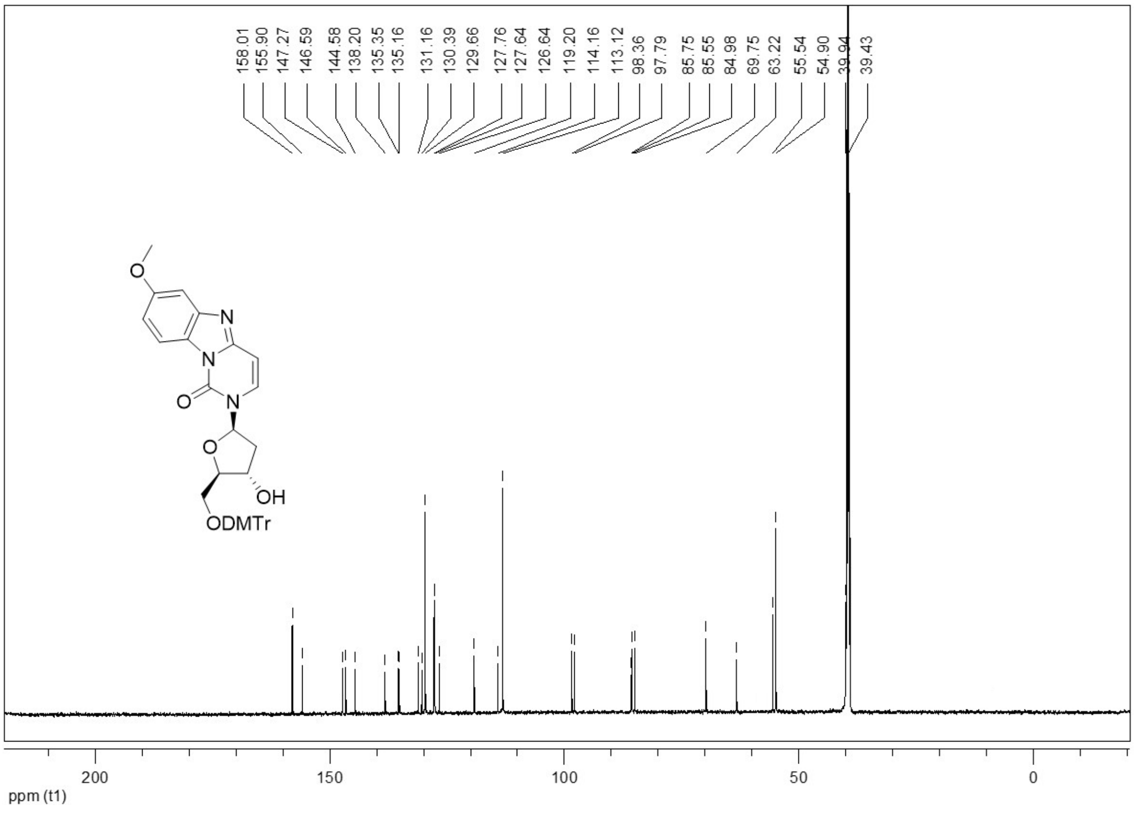

2-((2R,3R,4S,5R)-5-((bis(4-methoxyphenyl)(phenyl)methoxy)methyl)-3,4-dihydroxytetrahydrofuran-2-yl)-7-methoxybenzo[4,5]imidazo[1,2-c]pyrimidin-1(2H)-one **7b**

^{1}H NMR spectrum

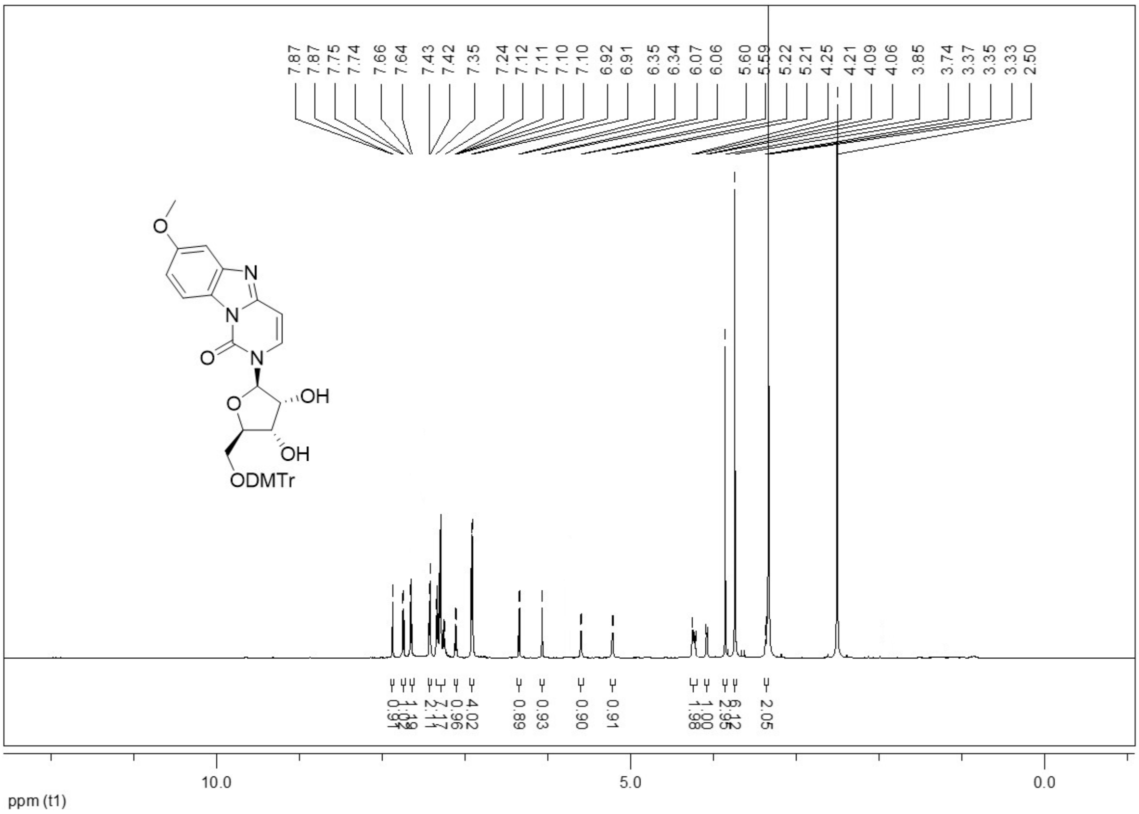

^{13}C NMR spectrum

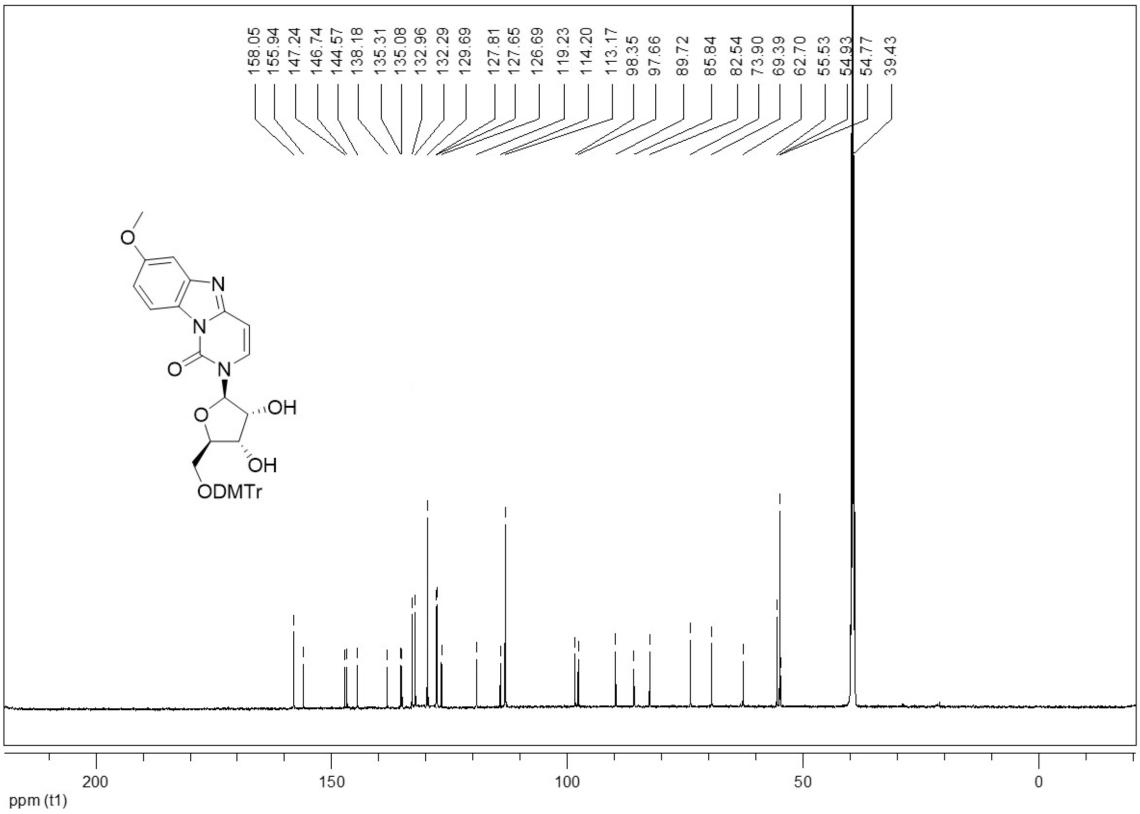

2-((2R,4S,5R)-4-hydroxy-5-(hydroxymethyl)tetrahydrofuran-2-yl)-7-methoxybenzo[4,5]imidazo[1,2-c]pyrimidin-1(2H)-one **8a**

^{1}H NMR spectrum

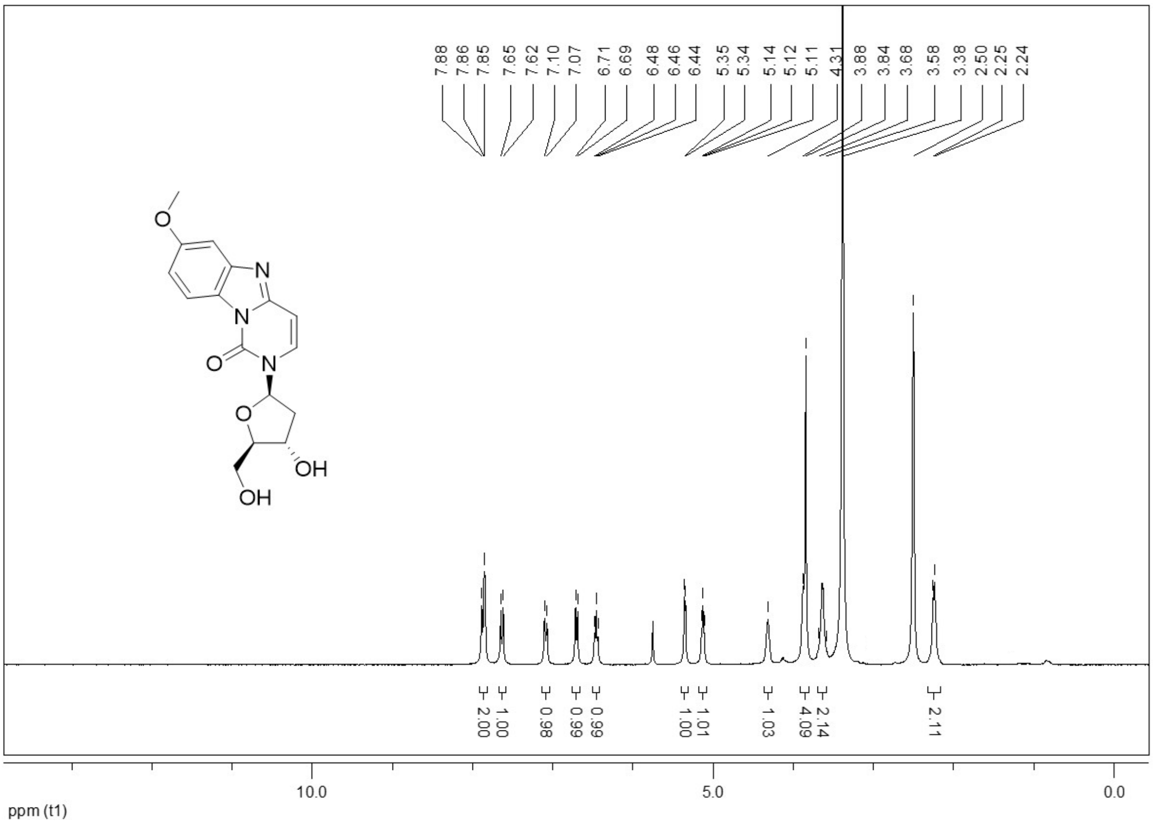

^{13}C NMR spectrum

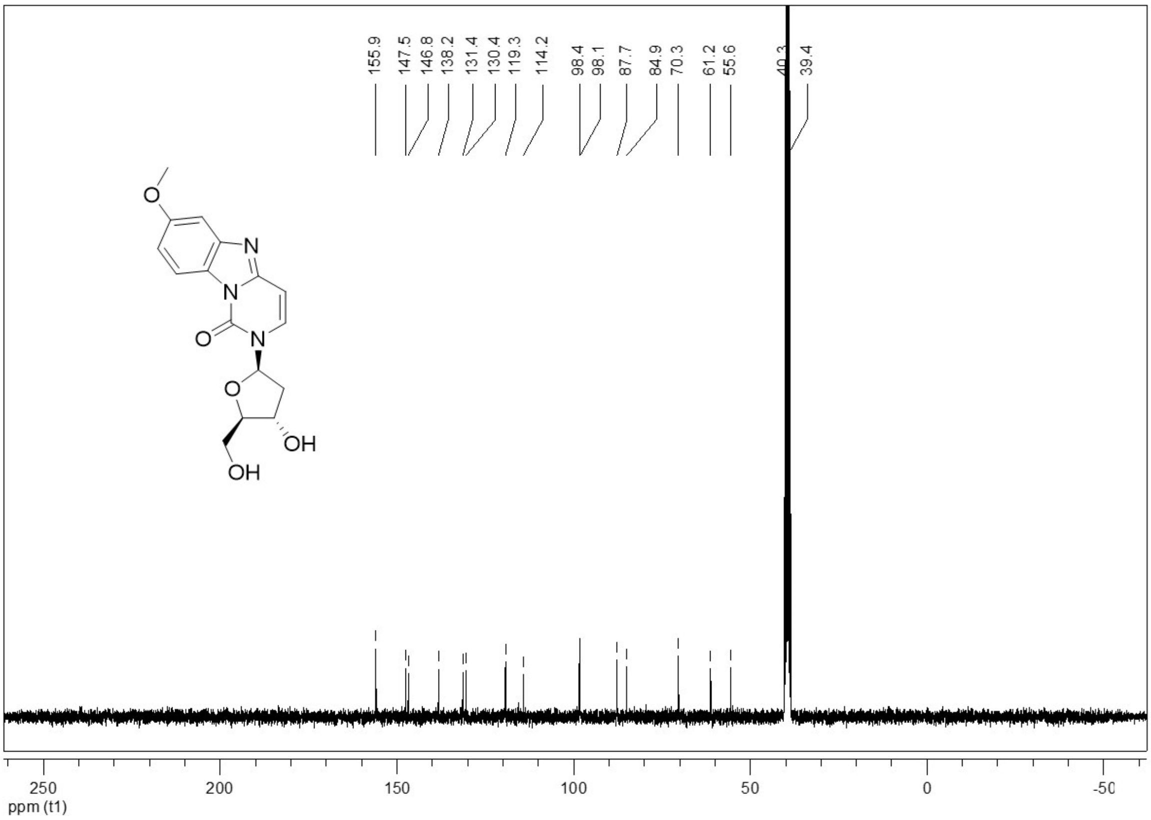

2-((2R,3R,4S,5R)-3,4-dihydroxy-5-(hydroxymethyl)tetrahydrofuran-2-yl)-7-methoxybenzo[4,5]imidazo[1,2-c]pyrimidin-1(2H)-one **8b**

^{1}H NMR spectrum

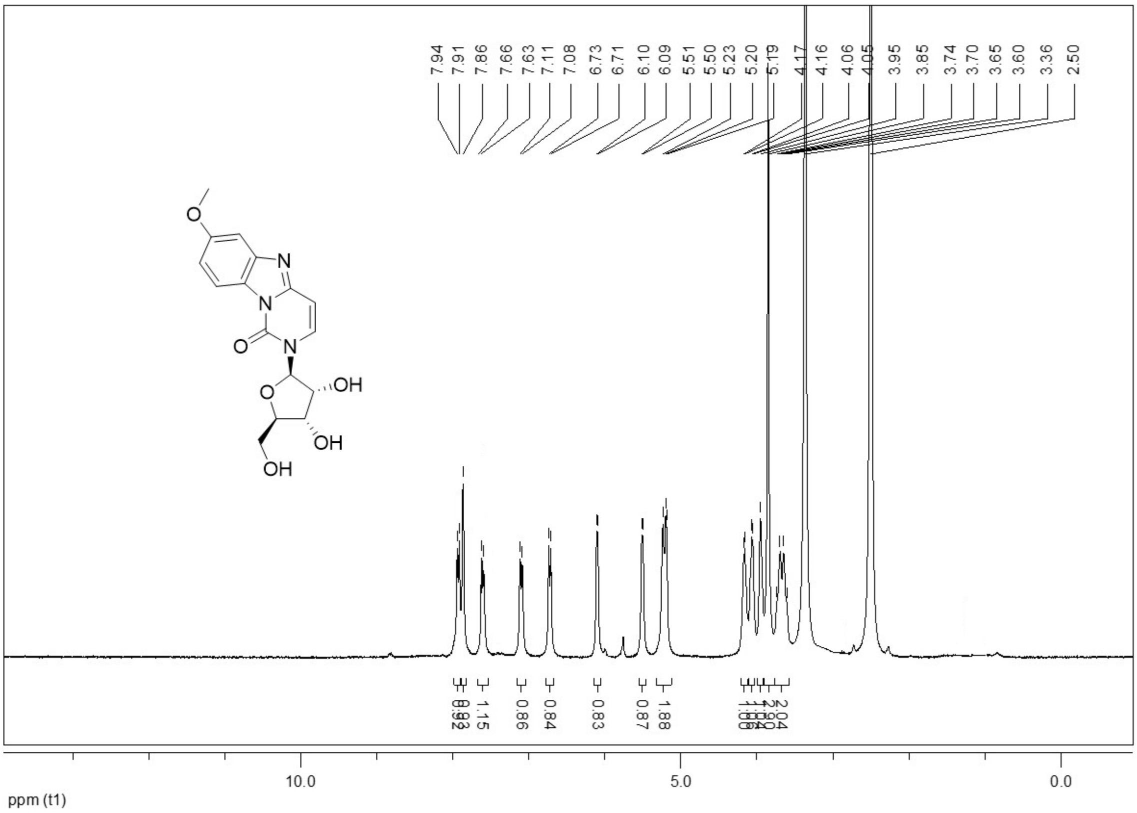

^{13}C NMR spectrum

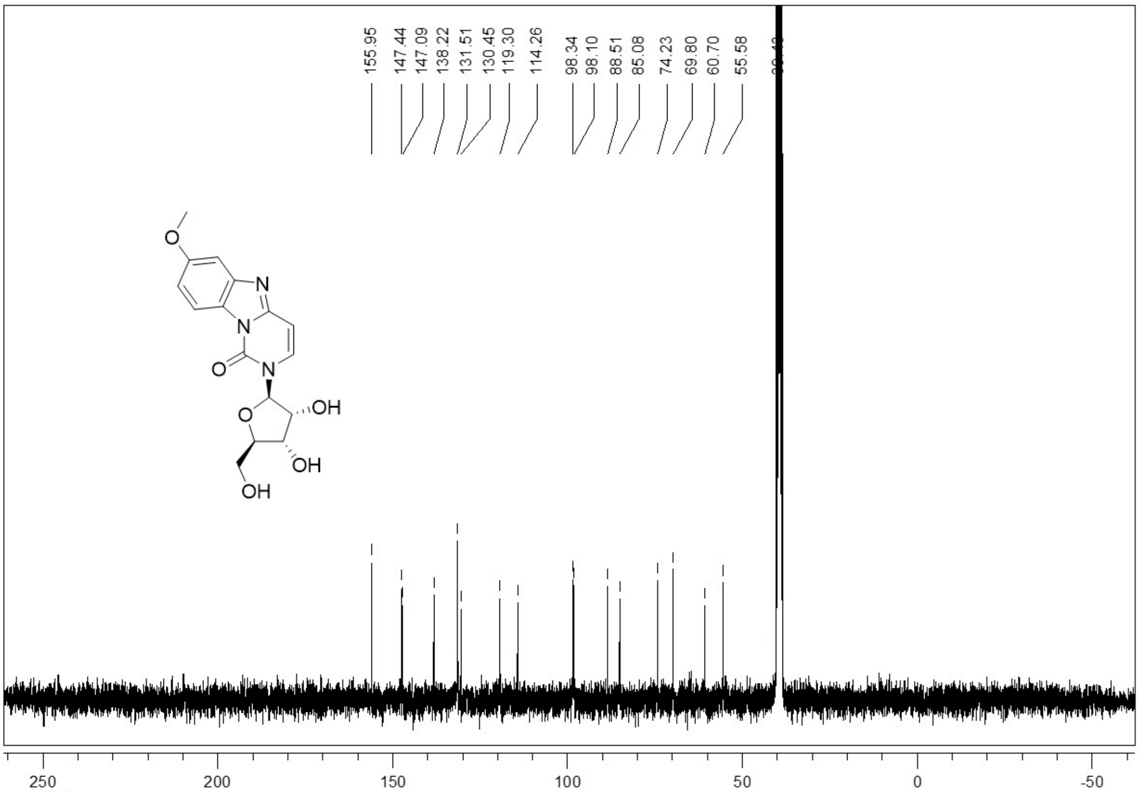

4-amino-1-((2R,4S,5R)-5-((bis(4-methoxyphenyl)(phenyl)methoxy)methyl)-4-hydroxytetrahydrofuran-2-yl)pyrimidin-2(1H)-one **9a**

^{1}H NMR spectrum

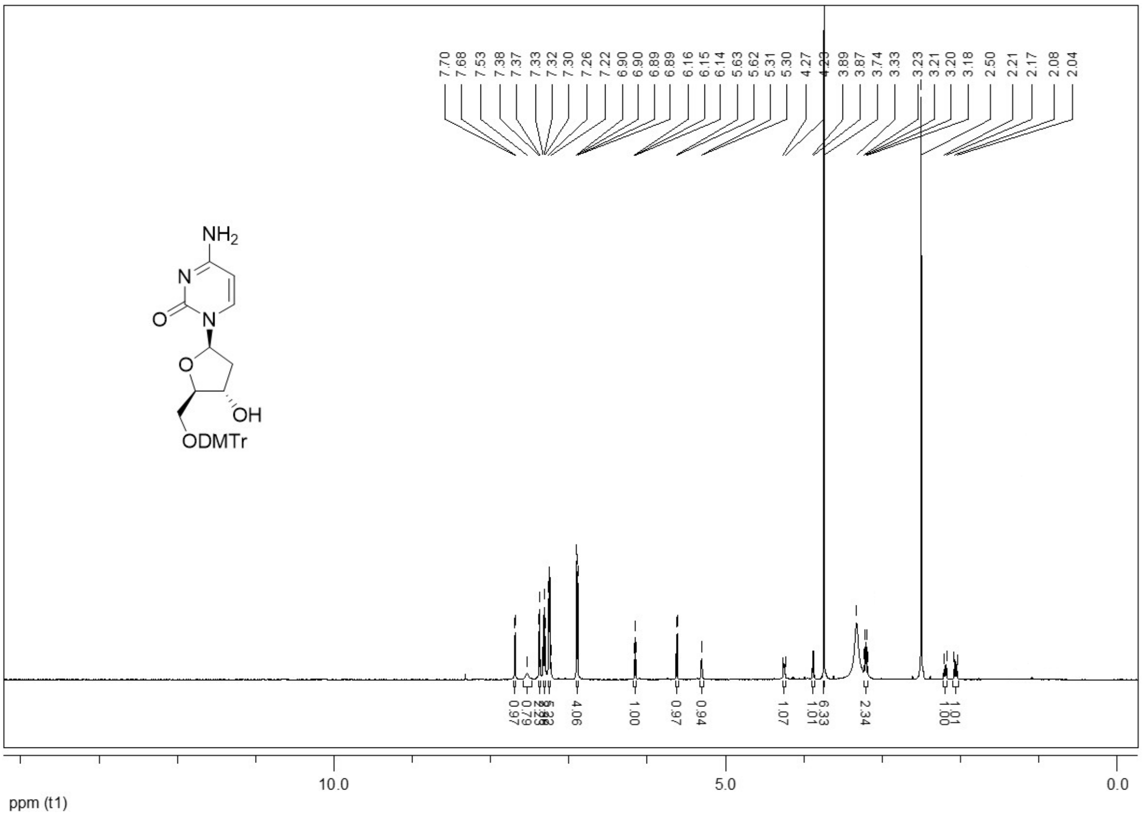

^{13}C NMR spectrum

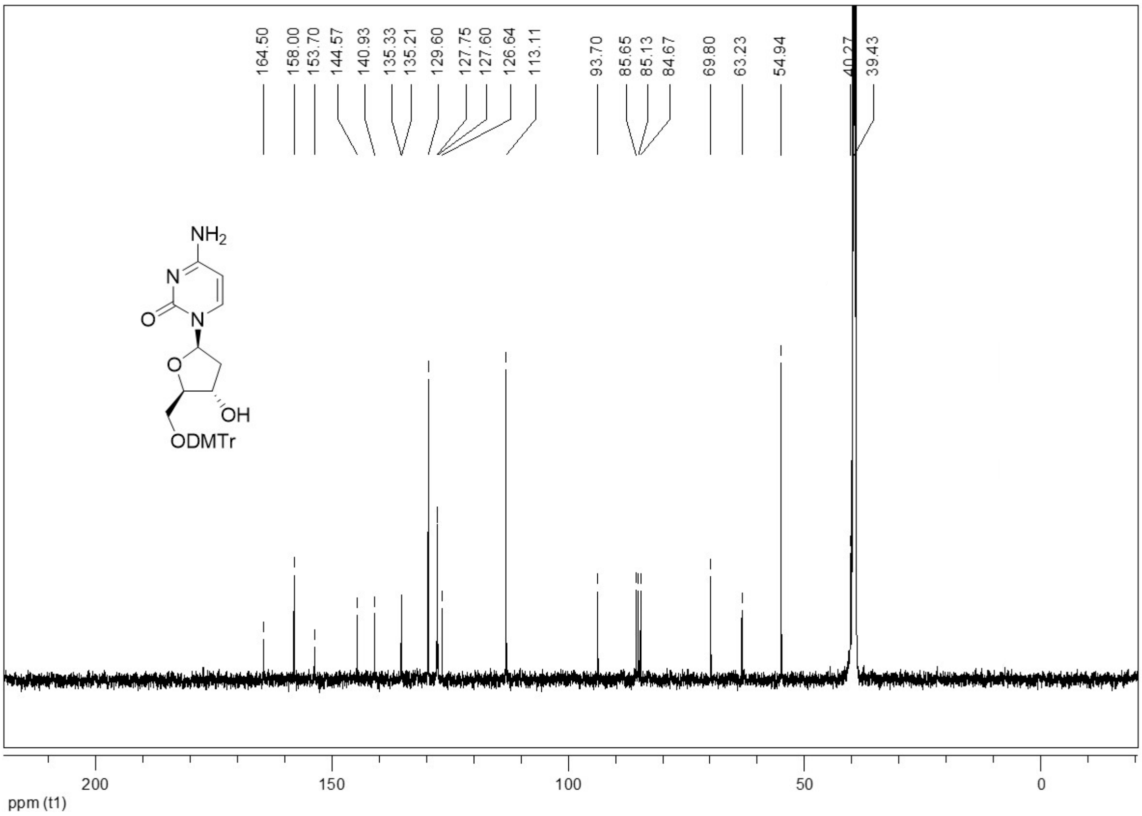

4-amino-1-((2R,3R,4S,5R)-5-((bis(4-methoxyphenyl)(phenyl)methoxy)methyl)-3,4-dihydroxytetrahydrofuran-2-yl)pyrimidin-2(1H)-one **9b**

^{1}H NMR spectrum

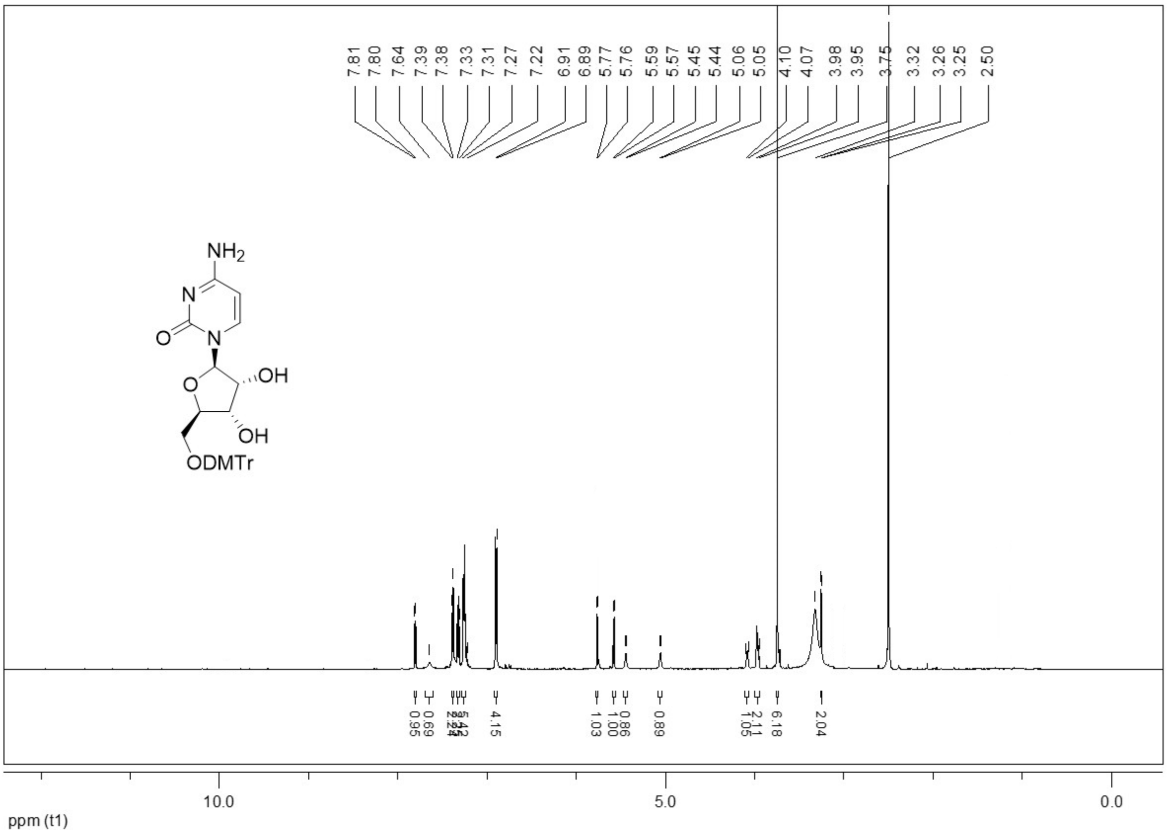

^{13}C NMR spectrum

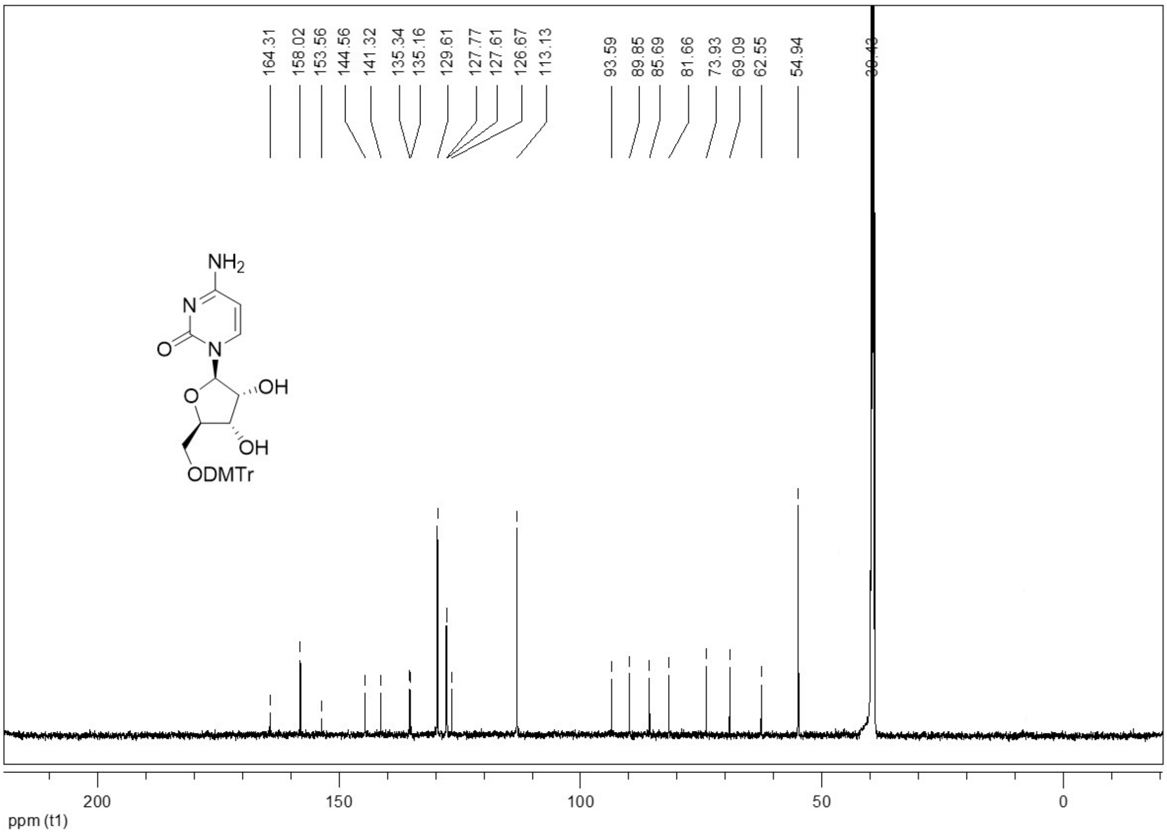

Supplement: Supplementary file 1 [file ijms-24-14540-s001.zip › ijms-2594540-supplementary.pdf]
